# A First Diversity-Oriented *N*-Maleopimarimido-Isocyanide for Multicomponent Reactions: Synthesis, Application, and In Silico Evaluation

**DOI:** 10.3390/ijms27083494

**Published:** 2026-04-14

**Authors:** Elena Tretyakova, Anna Smirnova, Oxana Kazakova

**Affiliations:** Ufa Institute of Chemistry, Ufa Federal Research Centre, Russian Academy of Sciences, 71 Prosp. Oktyabrya, Ufa 450054, Russia; bazunova03@yandex.ru (A.S.); obf@anrb.ru (O.K.)

**Keywords:** abietane diterpenoids, maleopimarimide, isocyanide, multicomponent reactions, α-acyloxycarboxamides, α-acylaminocarboxamides, tetrazoles, SwissADME, ProTox-III

## Abstract

Multicomponent reactions with isocyanides (IMCRs) enable the one-step assembly of complex molecules and remain a powerful strategy for accessing bioactive scaffolds. Here, we report the first synthesis of an abietane diterpene isocyanide derived from aminoimide methyl maleopimarate **1**, a levopimaric acid-maleic anhydride adduct. This isocyanide was further engaged in Passerini, Ugi, and azido-Ugi reactions to provide a series of α-acyloxy- and α-acylaminocarboxamides, as well as tetrazoles, in high yields under optimized conditions. The structures of all products were confirmed by comprehensive physicochemical analysis. In silico ADME, drug-likeness, target prediction, and toxicity studies (SwissADME, ProTox-III) revealed moderate lipophilicity with favorable membrane permeability and solubility, high gastrointestinal absorption, and selective CYP3A4 inhibition with no significant effects on other CYP450 isoforms. The compounds fulfill major drug-likeness criteria, lacking undesirable reactive fragments, with only acceptable deviations in molecular weight and flexibility typical for MCR-derived products. The modifications broaden the spectrum of predicted biological targets while maintaining low overall toxicity and absence of predicted hepato- or carcinogenicity. These results demonstrate that diterpene isocyanide is a valuable building block for chemical libraries of structurally diverse abietane derivatives with peptide-like termini and highlight its potential as a source of cytotoxic, antiviral, and anti-inflammatory candidates.

## 1. Introduction

**Figure 1:**
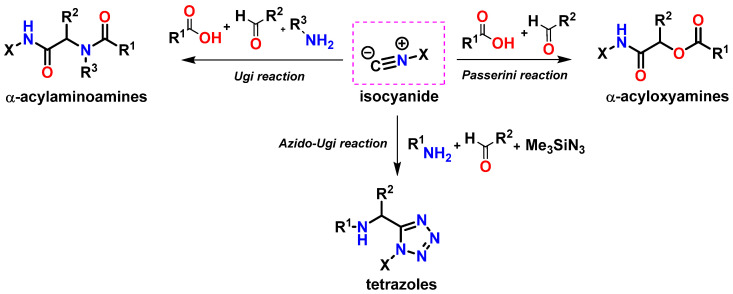
Passerini, Ugi and Azido-Ugi MCRs with isocynides.

In recent years, multicomponent reactions (MCRs) have emerged as effective tools for the rapid derivatization of various organic molecules for medical and chemical applications [1,2]. MCRs involving the addition of isocyanides to a polarized double bond (Passerini, Ugi, and its many variants) are among the most numerous representatives of these reactions (Figure 1) [3,4,5]. Combination of natural compounds and their derivatives via isocyanide-based MCRs (IMCRs) has attracted wide interest due to the rapid access to large libraries of diversity-oriented products with promising pharmacological potential [6,7,8,9,10,11,12,13]. Examples are known of the use of the carboxyl component of the labdane diterpenoid coronarin D [7], cholic [8] and triterpene acids [9,10,11] with the formation of corresponding α-acylamino- and α-acyloxy- derivatives capable of reducing nitric oxide production in RAW cells or inducing programmed tumor cell death by apoptosis and with significant cytotoxic properties in the range of low micromolar concentrations. New cytotoxic aminoamides with EC_50_ values in the nanomolar range [12] and bis-tetrazoles with a pronounced enzyme-inhibitory activity [13] have been synthesized from dehydroabietylamine as an amine component.

Previously, we developed the main approaches to obtain peptidomimetic and heterocyclic derivatives of abietane diterpene acids based on the Passerini, Ugi and azido-Ugi reactions [14,15,16,17,18]. Using the carboxyl group of dehydroabietic acid and levopimaric acid diene adducts in the Ugi and Passerini MCRs, new α-acyloxycarboxamides and α-acylaminocarboxamides were obtained, demonstrating a significant antiproliferative effect at submicromolar and micromolar concentrations (GI_50_ 0.42–3 µM) [17]. These compounds also effectively inhibited the influenza A (H1N1) virus (IC_50_ 2–32 µM, CC_50_ > 300 µM, SI 200) and SARS-CoV-2 pseudovirus [14]. Using the aminoimide methyl maleopimarate and the C4-hydrazone methyl 1β,13α-epoxy-2,3-dihydroquinopimarate as the amine component in the Ugi reaction or pseudo-seven-component azido-Ugi reaction, bis-substituted tetrazole derivatives with selective cytotoxicity against leukemia cell lines with a GI_50_ value in the range of 1.2–15.4 µM were successfully synthesized [15,18].

**Figure 2:**
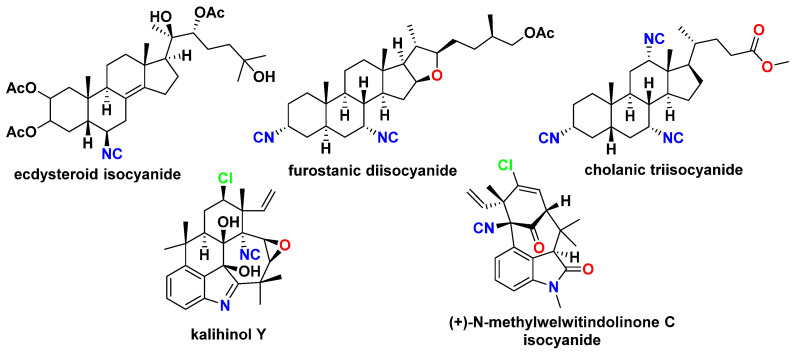
Synthesized steroid isocyanides and natural compounds containing the isocyanide functional group.

In order to avoid the formation of an additional stereocenter in the final Ugi products, formaldehyde or paraformaldehyde are most often used as the carbonyl component [19], and a wide range of commercially available compounds of the linear, cyclic and heterocyclic type are usually used as the isocyanide unit [20], while the literature data on the use of natural compounds as such an element is limited to a few reports. Natural sources of isocyanides include sea sponges and cyanobacteria belonging to a number of different genera [21]. These compounds exhibit a number of activities, such as antimalarial, antibacterial, antifungal, and antialgal activity [22]. In addition, attempts have been made to synthesize similar isocyanides, such as kalihinol Y [23] and (+)-N-methylwelwitindolinone C isocyanide [24]. Well-known examples are the production of isonitriles based on steroid frameworks, in particular the furostane, cholane and ecdysteroid series, and their subsequent conjugation with amino acids, peptides or their sequences on the cell surface under the conditions of the Ugi and Passerini reaction (Figure 2) [25,26,27,28,29]. We have not found any data on the use of diterpenoids, especially abietanes, as isonitrile components in IMCRs. However, the presence of a stable divalent carbon atom in the isocyanide group makes it an attractive building block for constructing chemical libraries of structurally diverse biomolecules [20]. Despite the fact that isocyanides are capable of entering into radical reactions and metallation reactions, nucleophilic and electrophilic addition reactions are most characteristic of them. It is this property that underlies such widely used MCRs as the Ugi, azido-Ugi, Passerini and some other reactions [1,2,3,4,5,6].

In this work, we report a method for the synthesis of a new abietane diterpene isocyanide based on the readily available aminoimide methyl maleopimarate and its use as a key component in the well-established IMCRs protocol for the synthesis of the corresponding α-acyloxycarboxamides, α-acylaminocarboxamides and 2*H*-tetrazoles. For the synthesized diterpene derivatives series, an in silico evaluation of the physicochemical properties (ADME, drug-likeness), prediction of biological targets and toxicological profile was performed using the SwissADME and ProTox-III web services.

## 2. Results and Discussion

The formation of an isonitrile group can be accomplished in a fairly large number of methods, including converting of alcohols, turning alkenes into isonitriles, opening of oxirane rings, under Hoffmann reaction conditions, etc. [30,31,32,33,34,35]; however, the main method to obtain isocyanides is the dehydration of the corresponding formamide derivative [36,37,38].

**Scheme 1:**
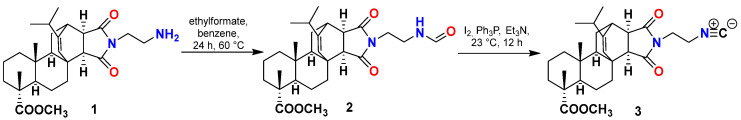
Synthesis of diterpene isocyanide **3**.

The search for optimal conditions and the development of the synthesis of the target isocyanide were conducted using a model compound, aminoimide methyl maleopimarate **1**, which has proven itself well when used as an amino component in the Ugi reaction and its modification so-called azido-Ugi [16,18]. When aminoimide **1** interacted with formic acid in benzene upon heating, the yield of the target product did not exceed 10%, whereas when formic acid was replaced by a 3 mmol excess of ethyl formiate for 24 h upon heating to 60 °C, the yield of compound **2** was quantitative (Scheme 1). In the ^1^H NMR spectra, a characteristic signal of the aldehyde group proton was observed at δ 8.10 ppm, which in the ^13^C NMR spectra correlated with the signal of the carbon atom at δ 161.5 ppm. IR bands at 1699 (C=O) and 2869, 2934 (C–H) cm^–1^ are characteristic of the formamide.

Next, we moved on to improve the conditions for diterpene isocyanide **3** synthesis. Table 1 shows a comparison of the reaction yields when using different dehydrating agents. Thus, in reactions of compound **2** with tosyl chloride and phosphorus oxychloride, the formation of the target compound either did not occur, or the reaction yields were insufficient. The simplest and most convenient method turned out to be the reaction of formamide **2** with iodine and triphenylphosphine, as a result of which isocyanide **3** was synthesized with a yield of 69% after purification by column chromatography. The ^1^H NMR spectra contains signals of the protons of the α-methylene unit at the isocyanide group in the region of δ 3.50–3.85 ppm, which in the ^13^C NMR spectra correlate with the carbon atom at δ 45.0 ppm. In addition, the ^13^C NMR spectra contain a carbon atom signal of the isonitrile group at δ 159.1 ppm. In addition, the IR spectra of compound **3** show an absorption at 2149 cm^−1^ that characteristic of the isocyanide [39].

Previous studies have described the synthesis of steroid isocyanides through the dehydration of the corresponding formamide derivative following reaction with toxic triphosgene or POCl_3_ at low temperatures; however, this leads to the formation of inseparable mixtures of epimers and is complicated by the dehydration of other hydroxyl groups within the steroid framework [26,27,28]. At the same time, our proposed simple and universal method for synthesis of diterpene isocyanides is characterized by mild conditions, high yields, availability of reagents and ease of scaling. In order to avoid losses that inevitably arise during chromatographic purification, in the subsequent Ugi condensation, we used isocyanide **3** without preliminary purification, which also increased the yield of the final reaction products.

**Scheme 2:**
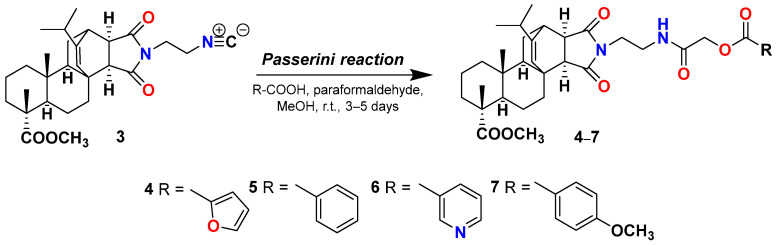
Synthesis of diterpene α-acyloxycarboxamides **4**–**7**.

Thus, the synthesized diterpene isocyanide **3** was immediately introduced into IMCRs. The three-component Passerini reaction was conducted at room temperature in methanol as a result of the interaction of paraformaldehyde, the corresponding carboxylic acids and isocyanide **3** (Scheme 2). The reaction mixture was kept at room temperature for 3–5 days. As a result, after chromatographic purification, α-acyloxycarboxamides **4**–**7** were obtained with a yield of 72–76%.

**Scheme 3:**
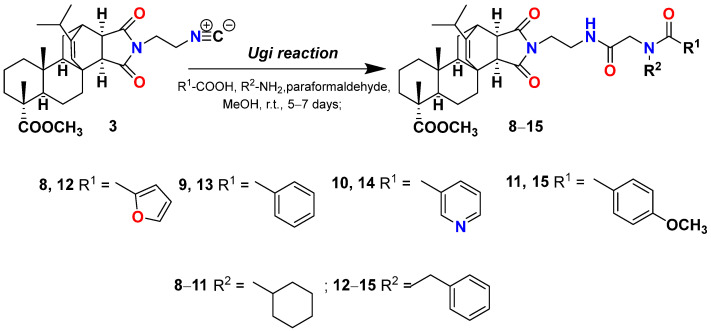
Synthesis of diterpene α-acylaminocarboxamides **8**–**15**.

The four-component Ugi reaction with equimolar amounts of paraformaldehyde, corresponding amine and carboxylic acids proceeds to form the products **8**–**15** (Scheme 3). The reaction proceeded smoothly in methanol at room temperature for 5–7 days (TLC control) with the complete conversion of the starting compound. The yields of the α-acylaminocarboxamides **8**–**15** after purification by column chromatography were up to 85%.

**Scheme 4:**
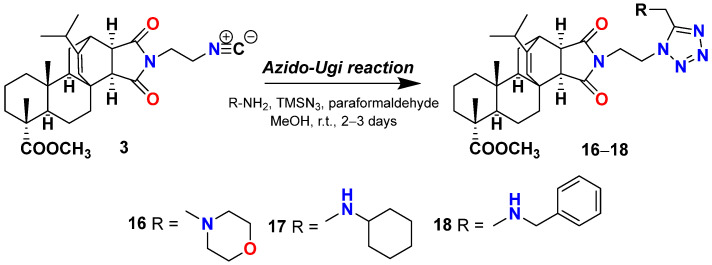
Synthesis of diterpene bis-substituted 1H-tetrazoles **16**–**18**.

The azido-Ugi reaction of isocyanide **3** with morpholine, cyclohexylamine or benzylamine and TMSN_3_ easily proceeds in methanol at room temperature for 2–3 days (TLC control) with the formation of target products **16**–**18** in high 80–89% yields (Scheme 4).

The complete identification of the structure of compounds **4**–**18** was made by the means of NMR spectroscopy and mass spectra, which exhibited molecular ions peaks corresponding to molecular masses of the compounds. The ^1^H NMR spectra of Passerini and Ugi products **4**–**15** NH-atom signals as broadened signals at δ 6.15–8.12 ppm were contained, and proton signals of carboxylic acid residues were observed at the region δ 6.55–8.90 ppm. In the ^13^C NMR spectra, carbon atom signals of keto groups appeared at the δ 160.5–177.8 ppm, the characteristic aromatic proton signals resonated at the δ 111.3–150.6 ppm, respectively. Mass spectra of tetrazoles **16**–**18** correspond to the molecular masses of mono-derivatives (quasi-molecule ion at *m*/*z* = 609.4, 622.4, 629.4 (100%, [M+H]^+^). ^1^H NMR and ^13^C NMR spectra demonstrate the expected structure skeleton of methyl maleopimarate amidoimide, signals from amine residues, and in ^13^C NMR spectra, signals typical of the tetrazole ring are detected at δ 151.7–153.9 ppm (Supplemental Materials, Figures S1–S115).

Thus, the developed synthetic approach provides an efficient and versatile route to a new class of diterpene isocyanides and their functional derivatives. The method based on the dehydration of formamide **2** using iodine and triphenylphosphine offers mild conditions, high yields, and easy scalability. The obtained isocyanide **3** demonstrates high reactivity in IMCRs such as Passerini, Ugi, and azido-Ugi, leading to a wide range of structurally diverse peptide-like abietane diterpene derivatives **4**–**18**. These results highlight the potential of diterpene isocyanides as valuable building blocks for the creation of compound libraries with prospective biological activity.

To evaluate the prospects for using the synthesized compounds as medicinal agents, the next stage of the work was preliminary in silico modeling of their physicochemical properties, pharmacokinetic profile (ADME), compliance with drug-likeness criteria, prediction of biological targets and toxicological safety using specialized web services SwissADME and ProTox-III [40,41]. The presented analysis is preliminary and theoretical in nature and serves as the basis for selecting the most promising compounds for further preclinical studies. Experimental validation of ADME properties, biological activity, and toxicity is planned for the next stage of the study.

### 2.1. Comprehensive ADME and Drug-likeness Evaluation of Synthesized Compounds Using SwissADME

#### 2.1.1. Evaluation of Physicochemical Characteristics

The physicochemical properties of the compounds **1**–**18** are given in Table S1 (Supplementary Materials). Polar surface area (PSA) is determined using the topological polar surface area (TPSA), which accounts for sulfur and phosphorus as polarizable atoms [42]. As shown in Table S1 the diterpene α-acyloxycarboxamides **4** and **6** have the highest TPSA values. The rotatable bond number (nRB) counts single bonds allowing free rotation, excluding those in rings, terminal bonds to heavy atoms, and amido CN bonds due to their high rotational energy barriers [43]. Molecules should contain no more than 9 rotatable bonds and all compounds possess at most 1 such bond.

#### 2.1.2. Lipophilicity and Water Solubility

The lipophilicity and water solubility of the diterpene compounds **1**–**18**, calculated using the SwissADME consensus models (ESOL, Ali, SILICOS-IT), are given in the Table S2 (Supplementary Materials). As noted in the literature, the lipophilicity of compounds plays a crucial role in molecular discovery efforts across diverse fields [44]. The preferred distribution of the synthesized diterpene derivatives in an aqueous medium is shown by their Log Po/w values, which ranged from 3.35 (tetrazole **16**) to 5.68 (Ugi product **9**). Low log Po/w values (3.35–3.56) for compounds **1**–**3** and **16** indicate an acceptable balance of permeability and solubility, while high ones (5.04–5.68) for compounds **8**–**15** suggest a potential for membrane transport, but with a risk of aggregation. As for the solubility in water, characterized by LogS values on three models (ESOL, Ali, SILICOS-IT), which in this series are predominantly negative (from −4.53 to −9.78), which classifies compounds **4–15** and **17**–**18** as low-soluble. Only compounds **1**–**3** and **16** show moderately soluble properties across all models, which correlates with lower Log Po/w and is typical for modified diterpenoids with potential bioactivity [45].

Thus, the calculated Log Po/w values in the range of 3.35–5.68 fit into the drug-like space (≤5 according to Lipinsky), but high values > 5 require optimization of the synthesized compounds, for example, the introduction of polar substituents, to improve solubility.

#### 2.1.3. Characteristics of Pharmacokinetics

Pharmacokinetics plays a pivotal role in achieving the desired therapeutic effect of a drug, as each compound’s pharmacokinetic profile can significantly shape its overall pharmacological performance [46]. Table 2 presents the pharmacokinetic parameters and bioavailability indices for derivatives **1**–**18** calculated using SwissADME, allowing for the evaluation of their suitability as orally active dosage forms in early-stage studies. These indicators include the degree of gastrointestinal absorption (GI abs.), bioavailability score (Bioavail. Score), blood–brain barrier permeability (BBB per), P-gp substrate activity, and potential inhibition of major CYP450 isoforms.

Most compounds in the studied series exhibit high or satisfactory GI absorption. High absorption is predicted for derivatives **1**–**3**, **5**, and **15**–**18**, in contrast to compounds **4** and **6**–**14**, reflecting the influence of increased molecular weight, polar surface area, and flexibility on intestinal barrier penetration. The bioavailability score for a significant portion of the structures is 0.55 (compounds **1**–**4**, **6**–**8**, **10**, **12**, **14**, **17**, and **18**), which corresponds to typical values for drug-like molecules, while compounds **5**, **9**, **11**, **13**, **15**, and **16** have a reduced score of 0.17, indicating more pronounced limitations in oral bioavailability. Of all the diterpene analogs, only isocyanide **3** is predicted to permeate the blood–brain barrier, while the other compounds are predicted to be impermeable, reducing the risk of central side effects with systemic administration.

All compounds **1**–**18** are identified as P-gp substrates, which may limit their effective absorption and tissue penetration via efflux transport, but simultaneously contribute to protection against accumulation in sensitive organs and a reduced risk of toxicity. The studied series will likely require consideration of interactions with P-gp when planning further pharmacokinetic studies and possible structural modifications to partially bypass efflux. In terms of CYP450 system metabolism, all derivatives **1**–**18** are predicted to be non-inhibitors of CYP1A2, CYP2C19, CYP2C9, CYP2D6 and CYP3A4, except for moderate inhibition of CYP3A4 noted for aminoimide **1**, formamide **2** and its products **4**–**15**.

In summary, the derivatives **1**–**18** generally exhibit a favorable pharmacokinetic profile with acceptable GI absorption, moderate to high predicted bioavailability, and a relatively low risk of significant drug interactions via the CYP system, despite potential limitations associated with P-gp-mediated efflux.

#### 2.1.4. Drug-Likeness and Medicinal Chemistry

Table S3 presents data summarizing drug-likeness and key medicinal chemistry parameters for compounds **1**–**18**, including compliance with the Lipinsky, Ghose, Veber, Egan, and Muegge rules, the presence of PAINS/Brenk alerts, lead-likeness criteria, and synthetic availability assessment [40].

Initial compounds **1**–**3** demonstrate the best accordance to classical drug-likeness rules, fully satisfying the Lipinsky, Veber, Egan, and Muegge criteria. Ghose filter violations are primarily due to elevated molecular weights and atomic numbers, rather than extreme lipophilicity or size. These compounds lack PAINS alerts, and the number of Brenk signals is limited to a few fragments, indicating an acceptable risk of reactivity and nonspecific binding. The lead-likeness criterion is violated due to a molecular weight above 350 and, in part, increased lipophilicity. For Passerini products **4**–**7**, a gradual deterioration in formal compliance with a number of filters is observed due to an increase in molecular weight and the number of rotatable bonds. Thus, most compounds exhibit one Lipinski violation, three Ghose violations, and one Veber violation. Egan and Muegge, however, most often yield either a full compliance or single violations, reflecting a balance between the polar surface and lipophilicity. PAINS alerts are completely absent, and Brenk signals are associated primarily with esters and phthalimide moieties. Ugi products **8**–**15** exhibit an even more pronounced shift beyond the strict lead-like criteria. Thus, α-acylaminoamides **8**, **9**, **11**, **13**, and **15** already have two Lipinski violations, as well as up to four Ghose violations due to a combination of high mass, volume, and a large number of atoms. Nevertheless, most of these compounds remain valid according to Veber and Egan, while Muegge violations are primarily related to high molecular weight and lipophilicity, while the absence of PAINS and a limited number of Brenk alerts indicate their acceptable profile. Tetrazole **16**–**18** partially retain profiles similar to their Ugi analogs but exhibit a somewhat more favorable balance of polarity and mass in terms of Egan and Muegge. For compound **16**, Lipinski violations are associated with molecular mass and the presence of more than 10 nitrogen and oxygen atoms; however, it satisfies the Veber and Egan rules and exhibits only one Muegge violation. Tetrazoles **17** and **18**, despite their higher mass and lipophilicity, fully comply with the Veber and Egan rules and retain only one or two Muegge violations, making them more promising in terms of oral bioavailability compared to their Ugi derivatives. In all three cases, PAINS alerts are absent, and Brenk signals are limited to fragments typical for a number of compounds.

Synthetic accessibility evaluation shows values ranging from 5.78 to 7.26 for the entire series of compounds, corresponding to moderately complex medicinal chemistry compounds typical of polyfunctional terpene hybrids. Lower values (5.8–6.0) for initial compounds **1**–**3** reflect their relatively less crowded structures, while higher values (>7.0) for compounds **8**–**12** and **15**–**17** indicate increased complexity with MCR modifications.

Overall, despite violations of strict lead-like criteria, primarily in terms of mass and lipophilicity, all compounds retain an acceptable drug-likeness profile without structures typical of false-positive results, making them viable candidates for further biological studies and targeted structure optimization.

**Figure 3:**
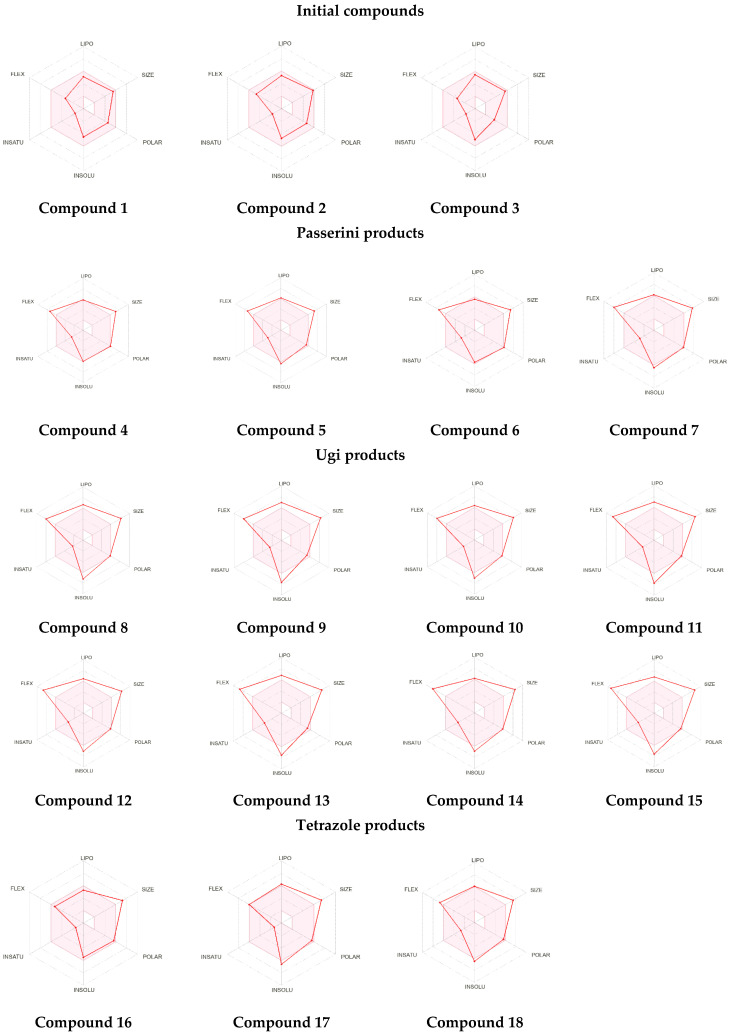
Analysis of drug-likeness properties of compounds **1**–**18** depending on the physicochemical properties. The radar charts, generated using http://www.swissadme.ch (accessed on 15 January 2026), represent the drug-likeness properties of **1**–**18**. Each radar chart evaluates the compounds based on six key parameters: lipophilicity (LIPO), size, polarity (POLAR), insolubility (INSOLU), insaturation (INSATU), and flexibility (FLEX). The shaded red areas illustrate how well each compound aligns with the optimal drug-likeness space according to these parameters.

Figure 3 presents a comparative evaluation of the drug-likeness profiles of compounds **1**–**18** using radar plots that visualize their key physicochemical parameters. These include lipophilicity (LIPO), molecular size (SIZE), polarity (POLAR), solubility (INSOLU), degree of saturation (INSATU) and molecular flexibility (FLEX), which together shape the likelihood of the compounds to function as orally available drugs. It is clearly demonstrated that most diterpene derivatives **1**–**18** generally fit satisfactorily within the optimal drug property space defined by these parameters, indicating their potential suitability for oral administration. However, for different groups of derivatives (parent compounds, Passerini and Ugi products, and tetrazole analogs), specific profile shifts are observed, reflecting the influence of structure on the balance of lipophilicity, size, polarity, and flexibility. Thus, starting diterpenes **1**–**3** exhibit balanced size and lipophilicity, forming compact radar profiles that fall well within the optimal range, indicating a favorable combination of membrane permeability and solubility, which are important for bioavailability. Minor deviations in polarity and flexibility remain within acceptable limits, suggesting no significant limitations for further optimization. Passerini products **4**–**7** are characterized by expansion along the SIZE and LIPO axes, reflecting an increase in molecular weight and lipophilicity compared to the parent structures, which should enhance their ability to penetrate lipid barriers. At the same time, some compounds exhibit an increase in INSOLU and a moderate shift in POLAR, which may limit their dissolution in the aqueous environment of the gastrointestinal tract and should be taken into account during dosage form development. Ugi products **8**–**15** form radar patterns with a marked increase in lipophilicity and size, as well as variability in flexibility, which is typical of MCR products. Increased flexibility of individual structures can favor adaptive interactions with targets but simultaneously increase entropic losses during binding. Furthermore, high INSOLU values indicate a risk of low solubility, making solubilization approaches and excipient selection critical. Finally, tetrazole derivatives **16**–**18** exhibit a more consistent POLAR and INSATU profile, reflecting the contribution of the tetrazole moiety to increased polarity and the controlled degree of saturation of the skeleton. These compounds are characterized by a more balanced compromise between lipophilicity, polarity, and flexibility, which should theoretically maintain an acceptable level of oral absorption, although localized increases in INSOLU in some compounds indicate possible solubility limitations.

Thus, all compounds have significant potential as drug candidates, but for certain structures, solubility and molecular flexibility are key limiting factors. These properties should be considered during further structural refinement and dosage form development, especially when considering applications where the delicate balance between lipophilicity, polarity, and flexibility will determine the optimal pharmacokinetic and pharmacodynamic profile.

#### 2.1.5. Biological Target Prediction

**Figure 4:**
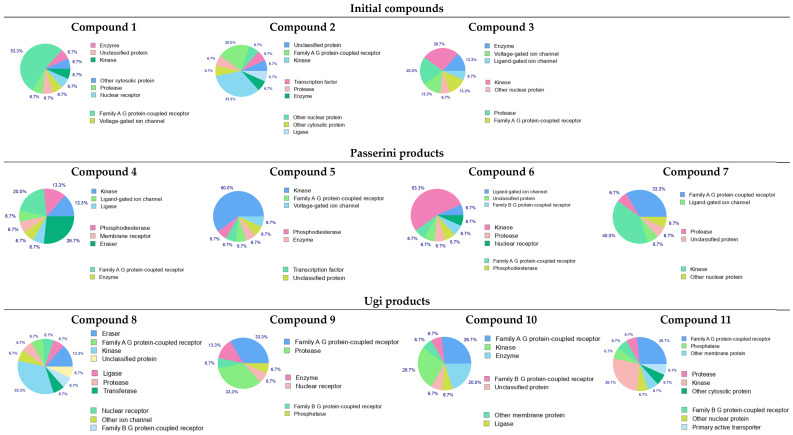

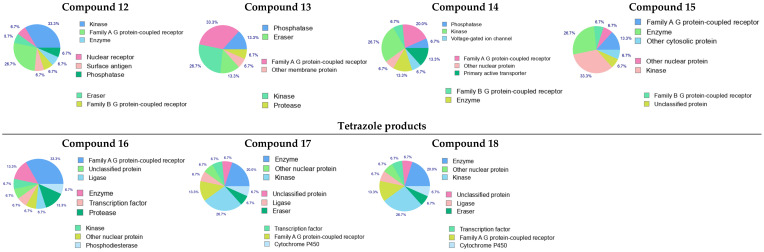
Biological target predictions for compounds **1**–**18**. (Pie charts display probabilities for top-15 target classes. Minor deviations from 100% (±0.1–0.3%, sum <100% or >100%) result from rounding errors in probability calculations, selective visualization of highest-confidence predictions, and potential overlaps in target classification).

All the synthesized diterpene compounds **1**–**18** were subjected for biological target prediction. Figure 4 presents the results of a predictive analysis of biological targets for compounds, allowing us to assess the diversity and selectivity of their potential pharmacological actions. Visualization in the form of pie charts for each structure demonstrates the distribution of putative targets across major protein classes, facilitating comparison of the profiles of the parent compounds, Passerini products, Ugi products, and tetrazole derivatives. The starting diterpenes **1**–**3** are characterized by a relatively moderate diversity of putative targets, with a significant proportion of predictions relating to enzymes and receptors traditionally involved in the regulation of cell proliferation and inflammatory processes. This distribution is consistent with their original natural origin and previously described biological activities of similar scaffolds [14,15,16,17], making them a convenient starting point for further targeted modification. Passerini analogs **4**–**7** exhibit a significant expansion of the spectrum of putative biological targets compared to the parent compounds, reflected in the increased proportion of various protein classes in the diagrams. In some cases, an increased contribution of kinases, oxidoreductases, and membrane receptors is observed, which may be due to increased structural complexity and the appearance of additional functional groups capable of forming new types of interactions with proteins [47]. Ugi products **8**–**15** demonstrate an even more pronounced increase in target diversity, with many representatives showing a more even distribution of predictions across multiple target classes. This profile indicates potential for pleiotropic action and the ability to influence multiple signaling pathways simultaneously. However, it may also pose a risk of decreased selectivity, which is important to consider when further selecting candidates and planning biological trials. Tetrazole derivatives **16**–**18** are distinguished by a somewhat more uniform and, in some cases, more focused target profile, as evidenced by the dominance of one or two target classes in the diagrams. The contribution of the tetrazole moiety, which enhances polar and ionic interactions [48], likely causes a bias toward certain enzyme and receptor families, which may be beneficial in the search for more selective candidates.

Overall, the diterpene products of IMCRs exhibit a richer and more structure-dependent spectrum of predicted targets compared to the parent compounds. This demonstrates the high plasticity of the backbone and the potential for fine-tuning the biological profile by varying substituents. Key parameters for further selection will be a combination of target diversity and sufficient selectivity for the most promising protein classes.

### 2.2. Toxicity

Using the ProTox-III server [41], LD_50_ values, acute toxicity classes, and probabilities of organ-specific toxic effects (hepatotoxicity, carcinogenicity, immunotoxicity, mutagenicity, and cytotoxicity) were predicted for the synthesized compounds **1**–**18** (Table 3). According to calculations, most compounds belong to acute toxicity classes 4–5 (LD_50_ = 1000–4000 mg/kg), which correspond to a moderate or low level of toxicity, while compounds **16** and **17**, which have the highest LD_50_ values (9500 and 10,000 mg/kg, respectively), are assigned to class 6 and demonstrate the best prediction for acute toxicity. The average similarity to the training set and prediction accuracy for these models are in the ranges of 44.64–73.73% and 54.26–69.26%, respectively, indicating acceptable reliability of the computational estimates.

Organotoxicity analysis showed that significant hepatotoxicity is not expected for most compounds (negative predictions with high probabilities of 0.60–0.88), and even for structures with higher general toxicity according to LD_50_, the risk of liver damage remains low. At the same time, potential immunotoxicity is predicted for a number of compounds (positive values with probabilities up to 0.99) with a remaining low or moderate risk of carcinogenicity and mutagenicity (in most cases, negative or moderately positive probabilities). For cytotoxicity, a predominantly low or moderate risk is noted—most compounds are characterized by a negative prediction with probabilities of approximately 0.51–0.72, while for tetrazoles **16**–**18**, modeling indicates possible cytotoxic activity with probabilities of about 0.53–0.57.

Thus, the data presented in Table 3 indicate a predominantly moderate or low level of acute toxicity of the studied compounds in the absence of pronounced hepatotoxicity and carcinogenicity, which, in combination with controlled risks of immuno- and cytotoxicity, supports their acceptable predicted safety profile.

## 3. Materials and Methods

The spectra were recorded at the Centre for the Collective Use “Chemistry” of the UIC UFRC RAS and RCCU “Agidel” of the UFRC RAS. ^1^H and ^13^C-NMR spectra were recorded on a “Bruker AM-500” (Bruker BioSpin, Billerica, MA, USA, 500 and 125.5 MHz respectively, ppm, Hz) in CDCl_3_, internal standard tetramethylsilane. Mass spectra were obtained on a liquid chromatograph–mass spectrometer LCMS-2010 EV (Shimadzu, Kyoto, Japan). Melting points were detected on a micro table “Rapido PHMK 05”(Nagema, Dresden, Germany). Optical rotations were measured on a polarimeter “Perkin-Elmer 241 MC” (Perkin Elmer, Waltham, MA, USA) in a tube length of 1 dm. Elemental analysis was performed on a Euro EA-3000 CHNS analyzer (Eurovector, Milan, Italy); the main standard is acetanilide. IR spectra were recorded on a spectrometer IR Prestige-21 (Shimadzu, Kyoto, Japan), the sample was in the form of a film of solution (solvent CHCl_3_). HPLS was obtained on Nucleosil C18 HPLC column (Macherey-Nagel GmbH & Co. KG, Düren, Germany; 150 × 4.6 mm), acquired by acetonitrile/water (85:15). Thin-layer chromatography analyses were performed on Sorbfil plates (Sorbpolimer, Krasnodar, Russia), using the solvent system chloroform–ethyl acetate, 40:1. Substances were detected by 10% H_2_SO_4_ with subsequent heating to 100–120 °C for 2–3 min. All the reagents and solvents (Sigma-Aldrich, St. Louis, MO, USA) were purchased from standard commercial vendors and were used without any further purification. Aminoimide methyl maleopimarate **1** was synthesized from commercially available maleopimaric acid (CAS 510-39-4, Sigma-Aldrich, St. Louis, MO, USA) according to the described procedure [49].

Methyl 2-(2-formamidoethyl)-12-isopropyl-6,9a-dimethyl-1,3-dioxo-1,2,3,3a,4,5,5a,6,7,8,9,9a,9b,10,11,11a-hexadecahydro-3b,11-ethenonaphtho[2,1-e]isoindole-6-carboxylate **2**. Ethylformate (0.24 mL, 3 mmol) was added to a stirred solution of compound **1** (456 mg, 1 mmol) in dry benzene (30 mL). After stirring for 24 h at 60 °C, the reaction mixture was washed with aq. 10% Na_2_CO_3_ (2 × 10 mL). The organic phase was evaporated under vacuum to give **2**. Yield 474 mg (98%), mp 51 °C, [α]_20_^D^ −58 (c 0.05, CHCl_3_). ^1^H NMR (CDCl_3_, 500 MHz) δ: 0.47 (s, 3H, CH_3_-20); 0.79 (m, 1H, CH_2_-1_ax_); 0.84 (d, J 6.8, 3H, CH_3_-16); 0.92 (d, J 7.0, 3H, CH_3_-17); 1.03 (s, 3H, CH_3_-19); 1.09-1.69 (m, 12H, CH_2_-1_eq_, 2, 3, 6, 7_ax_, 11, CH-5, 9); 2.05–2.07 (m, 1H, CH-15); 2.32 (d, J 8.5, 1H, CH-21); 2.37 (dt, _1_J 3, _2_J 14.0, 1H, CH_2_-7_eq_); 2.65 (dd, _1_J 3.0, _2_J 8.5, 1H, CH-22); 2.94 (s, 1H, CH-12); 3.32–3.35 (m, 2H, CH_2_); 3.51–3.54 (m, 2H, CH_2_-1′, 2′); 3.65 (s, 3H, CH_3_-25); 5.39 (s, 1H, CH-14); 6.23 (br.s., 1H, NH); 8.10 (s, 1H, CHO). ^13^C NMR (CDCl_3_, 125.5 MHz) δ: 15.6 (C-20); 16.8 (C-19); 17.0 (C-2); 20.0 (C-16); 20.7 (C-17); 21.8 (C-6); 27.6 (C-11); 32.7 (C-15); 35.3 (C-12); 35.7 (C-7); 36.7 (C-3); 37.6 (C-1); 37.7 (C-10); 37.9 (C-8); 38.2 (C-1′); 40.8 (C-21); 45.0 (C-2′); 47.1 (C-4); 49.5 (C-5); 52.0 (C-25); 52.4 (C-22); 54.2 (C-9); 124.4 (C-14); 147.2 (C-13); 161.2 (CHO), 177.9 (C-24); 179.0 (C-23); 179.1 (C-18). Analysis calculated for C_28_H_40_N_2_O_5_: C, 69.39; H, 8.32; N, 5.78. Found: C, 69.42; H, 8.28; N, 5.86. MS (APCI) *m*/*z* (I_rel_, %): 485.4 [M+H]^+^ (100) (calculated for C_28_H_41_N_2_O_5_, 485.64). IR (ν max, cm^−1^): 1699 (C=O), 2869 (C-H), 2934 (C-H), 3384 (NH).

Methyl 2-(2-isocyanoethyl)-12-isopropyl-6,9a-dimethyl-1,3-dioxo-1,2,3,3a,4,5,5a,6,7,8,9,9a,9b,10,11,11a-hexadecahydro-3b,11-ethenonaphtho[2,1-e]isoindole-6-carboxylate **3**. Ph_3_P (393 mg, 1.5 mmol), I_2_ (380 mg, 1.5 mmol) and Et_3_N (0.42 mL, 3.0 mmol) were added to a stirred solution of compound **2** (485 mg, 1 mmol) in CH_2_Cl_2_ (30 mL). The reaction mixture was stirred for 48 h at 24 °C, concentrated, and isolated by column chromatography on silica gel. For the Ugi reaction, compound **3** was used without further purification. Yield: 322 mg (69%), mp 45 °C, [α]_20_^D^ −47 (c 0.05, CHCl_3_). ^1^H NMR (CDCl_3_, 500 MHz) δ: 0.49 (m, 1H, CH_2_-1_ax_); 0.51 (s, 3H, CH_3_-20); 0.84 (d, J 6.8, 3H, CH_3_-16); 0.92 (d, J 7.0, 3H, CH_3_-17); 1.07 (s, 3H, CH_3_-19); 1.09–1.85 (m, 12H, CH_2_-1_eq_, 2, 3, 6, 7_ax_, 11, CH-5, 9); 2.10–2.15 (m, 1H, CH-15); 2.50 (d, J 8.5, 1H, CH-21; 2.51 (dt, _1_J 3.0, ^2^J 14.0, 1H, CH_2_-7_eq_); 2.85 (dd, _1_J 3.0, _2_J 8.5, 1H, CH-22); 3.04 (s, 1H, CH-12); 3.50–3.85 (m, 4H, CH_2_-1′,2′); 3.65 (s, 3H, CH_3_-25); 5.42 (s, 1H, CH-14). ^13^C NMR (CDCl_3_, 125.5 MHz) δ: 15.6 (C-20); 16.7 (C-19); 17.0 (C-2); 20.0 (C-16); 20.7 (C-17); 21.7 (C-6); 27.5 (C-11); 32.6 (C-15); 32.7 (C-12); 35.1 (C-7); 35.5 (C-3); 36.3 (C-1); 36.7 (C-10); 36.9 (C-8); 38.1 (C-1′)); 40.5 (C-21); 45.1 (C-2′); 47.1 (C-4); 49.5 (C-5); 52.0 (C-25); 52.4 (C-22); 54.2 (C-9); 124.6 (C-14); 146.3 (C-13); 159.1 (NC), 176.6 (C-24); 178.0 (C-23); 179.1 (C-18). Analysis calculated for C_28_H_38_N_2_O_4_: C, 72.07; H, 8.21; N, 6.00. Found: C, 72.10; H, 8.25; N, 5.98. MS (APCI) m/z (I_rel_, %): 467.4 [M+H] ^+^ (100) (calculated for C_28_H_39_N_2_O_4_, 467.62). IR (ν max, cm^−1^): 1710 (C=O), 2149 (C≡N), 2865 (CH).

Synthesis of compounds **4**–**7**. General Procedure. Paraformaldehyde (1 equiv.) was suspended in MeOH (25 mL). The resulting mixture was stirred at r.t. for 1 h, and then the carboxylic acid ((0.20 mL, 1 mmol) 2-furoic acid for compound **4** or (122 mg, 1 mmol) benzoic acid for compound **5** or (123 mg, 1 mmol) nicotinic acid for compound **6** or (152 mg, 1 mmol) *p*-methoxy-benzoic acid for compound **7**) and the diterpene isocyanide **3** (466 mg, 1 mmol) were added. The reaction mixture was kept at room temperature for 3–5 days (TLC control). After completion of the reaction, the mixture was poured into acidified water, and the precipitate was filtered off, dried and purified by column chromatography.

Methyl 2-(2-(2-((furan-2-carbonyl)oxy)acetamido)ethyl)-12-isopropyl-6,9a-dimethyl-1,3-dioxo-1,2,3,3a,4,5,5a,6,7,8,9,9a,9b,10,11,11a-hexadecahydro-3b,11-ethenonaphtho[2,1-e]isoindole-6-carboxylate **4**. Yield: 463 mg (76%), mp 86 °C, [α]_20_^D^ −31 (c 0.05, CHCl_3_). ^1^H NMR (CDCl_3_, 500 MHz) δ: 0.50 (s, 3H, CH_3_-20); 0.82 (d, J 6.8, 3H, CH_3_-16); 0.73–0.99 (m, 1H, CH_2_-1_ax_); 0.84 (d, J 7.0, 3H, CH_3_-17); 1.07 (s, 3H, CH_3_-19); 1.00–1.85 (m, 12H, CH_2_-1_eq_, 2, 3, 6, 7_ax_, 11, CH-5, 9); 2.10–2.15 (m, 1H, CH-15); 2.50 (d, J 8.5, 1H, CH-21), 2.51 (dt, _1_J 3.1, _2_J 14.1, 1H, CH_2_-7_eq_); 2.85 (dd, _1_J 3.0, _2_J 8.5, 1H, CH-22); 3.04 (s, 1H, CH-12); 3.62–3.65 (m, 2H, CH_2_-2′); 3.65 (s, 3H, CH_3_-25); 3.92–4.10 (m, 2H, CH_2_-1′); 5.30–5.45 (m, 3H, CH-14, CH_2_-4′); 6.55 (br.s., 1H, CH-8′); 7.35 (br.s., 1H, CH-7′); 7.95 (br.s., 1H, CH-9′); 8.12 (br.s., 1H, NH). ^13^C NMR (CDCl_3_, 125.5 MHz) δ: 15.6 (C-20); 16.8 (C-19); 17.0 (C-2); 20.0 (C-16); 20.1 (C-17); 20.2 (C-6); 21.8 (C-11); 27.6 (C-12); 32.7 (C-15); 35.2 (C-7); 35.6 (C-3); 36.7 (C-1); 38.2 (C-10); 40.7 (C-8); 40.8 (C-21); 45.0 (C-1′); 45.1 (C-4); 47.2 (C-5); 49.5 (C-25); 52.0 (C-22); 52.5 (C-9); 54.1 (C-2′), 76.8 (C-4′), 111.6 (C-8′); 117.8 (C-7′); 124.4 (C-14); 144.7 (C-9′); 147.0 (C-6′); 147.1 (C-13); 161.4 (C-5′); 177.9 (C-3′); 178.8 (C-24); 179.0 (C-23); 179.2 (C-18). Analysis calculated for C_34_H_44_N_2_O_8_: C, 67.09; H, 7.29; N, 4.60. Found: C, 67.10; H, 7.28; N, 4.65. MS (APCI) *m*/*z* (I_rel_, %): 609.5 [M+H]^+^ (100) (calculated for C_34_H_45_N_2_O_8_, 609.31). IR (ν max, cm^−1^): 1696 (C=O), 2865 (CH), 2865 (CH), 3380 (NH).

Methyl 2-(2-(2-(benzoyloxy)acetamido)ethyl)-12-isopropyl-6,9a-dimethyl-1,3-dioxo-1,2,3,3a,4,5,5a,6,7,8,9,9a,9b,10,11,11a-hexadecahydro-3b,11-ethenonaphtho[2,1-e]isoindole-6-carboxylate **5.** Yield: 464 mg (75%), mp 65 °C, [α]_20_^D^ −11 (c 0.05, CHCl_3_). ^1^H NMR (CDCl_3_, 500 MHz) δ: 0.50 (s, 3H, CH_3_-20); 0.73–0.99 (m, 1H, CH_2_-1_ax_); 0.82 (d, J 6.8, 3H, CH_3_-16); 0.84 (d, J 7.0, 3H, CH_3_-17); 1.07 (s, 3H, CH_3_-19); 1.00–1.85 (m, 12H, CH_2_-1_eq_, 2, 3, 6, 7_ax_, 11, CH-5, 9), 2.10–2.15 (m, 1H, CH-15); 2.50 (d, J 8.5, 1H, CH-21), 2.51 (dt, _1_J 3.0, _2_J 14.0, 1H, CH_2_-7_eq_); 2.85 (dd, _1_J 3.0, _2_J 8.5, 1H, CH-22); 3.04 (s, 1H, CH-12); 3.30–3.49 (m, 2H, CH_2_-2′); 3.65 (s, 3H, CH_3_-25); 4.12–4.20 (m, 2H, CH_2_-1′); 5.35 (s, 1H, CH-14); 6.10 (br.s., 2H, CH_2_-4′); 7.22–7.51 (m, 5H, CH-Ar); 7.95 (br.s., 1H, NH). ^13^C NMR (CDCl_3_, 125.5 MHz) δ: 15.6 (C-20); 16.7 (C-19); 17.0 (C-2); 20.1 (C-16); 20.7 (C-17); 21.8 (C-6); 27.6 (C-11); 31.0 (C-12); 35.3 (C-15); 35.6 (C-7); 36.6 (C-3); 37.6 (C-1); 38.1 (C-10); 38.7 (C-8); 40.6 (C-21); 45.0 (C-1′); 46.2 (C-4); 47.1 (C-4′), 49. (C-5); 51.9 (C-25); 52.3 (C-22); 54.0 (C-9); 59.2 (C-2′), 123.3 (C-14); 127.4 (C-Ar); 127.8 (C-Ar); 129.0 (C-Ar); 129.9 (C-Ar); 131.4 (C-Ar); 132.3 (C-Ar); 146.2 (C-13); 160.5 (C-5′); 166.8 (C-3′); 176.9 (C-24); 178.0 (C-23); 178.2 (C-18). Analysis calculated for C_36_H_46_N_2_O_7_: C, 69.88; H, 7.49; N, 4.53. Found: C, 69.90; H, 7.48; N, 4.65. MS (APCI) *m*/*z* (I_rel_, %): 619.5 [M+H]^+^ (100) (calculated for C_36_H_47_N_2_O_7_, 619.33). IR (ν max, cm^−1^): 755 (Ar), 1740, 1771 (C=O), 2865 (CH), 3380 (NH).

Methyl 2-(2-(2-(nicotinoyloxy)acetamido)ethyl)-12-isopropyl-6,9a-dimethyl-1,3-dioxo-1,2,3,3a,4,5,5a,6,7,8,9,9a,9b,10,11,11a-hexadecahydro-3b,11-ethenonaphtho[2,1-e]isoindole-6-carboxylate **6.** Yield: 446 mg (72%), mp 82 °C, [α]_20_^D^ −18 (c 0.05, CHCl_3_). ^1^H NMR (CDCl_3_, 500 MHz) δ: 0.50 (s, 3H, CH_3_-20); 0.82 (d, J 6.8, 3H, CH_3_-16); 0.84 (d, J 7.0, 3H, CH_3_-17); 0.73–0.99 (m, 1H, CH_2_-1_ax_); 1.07 (s, 3H, CH_3_-19); 1.00–1.85 (m, 12H, CH_2_-1_eq_, 2, 3, 6, 7_ax_, 11, CH-5, 9), 2.10–2.15 (m, 1H, CH-15); 2.50 (d, J 8.5, 1H, CH-21); 2.51 (dt, _1_J 3.0, _2_J 14.0, 1H, CH_2_-7_eq_); 2.85 (dd, _1_J 3.0, _2_J 8.5, 1H, CH-22); 3.02 (s, 1H, CH-12); 3.30–3.42 (m, 2H, CH_2_-4′); 3.49–3.50 (br.s., 2H, CH_2_-2′); 3.65 (s, 3H, CH_3_-25); 3.92–4.10 (m, 2H, CH_2_-1′); 5.35 (s, 1H, CH-14); 6.15 (br.s., 1H, NH); 7.30 (br.s., 1H, CH-8′); 7.79–7.81 (m, 1H, CH-7″); 8.80 (br.s., 1H, CH-7′); 8.90 (br.s., 1H, CH-9′). ^13^C NMR (CDCl_3_, 125.5 MHz) δ: 15.6 (C-20); 16.7 (C-19); 17.0 (C-2); 20.1 (C-16); 20.7 (C-17); 21.7 (C-6); 27.6 (C-11); 32.7 (C-15), 35.2 (C-7); 35.6 (C-12); 36.7 (C-3); 37.6 (C-10); 38.1 (C-1); 39.1 (C-4′); 40.6 (C-8); 45.0 (C-4); 46.1 (C-1′); 47.1 (C-5), 49.4 (C-22); 51.9 (C-25); 52.4 (C-21); 54.0 (C-9); 59.4 (C-2′), 123.3 (C-Ar); 124.4 (C-14); 132.1 (C-Ar); 134.6 (C-Ar); 147.1 (C-13); 147.5 (C-Ar); 150.6 (C-Ar); 169.5 (C-5′); 170.1 (C-3′); 178.0 (C-24); 178.8 (C-23); 179.1 (C-18). Analysis calculated for C_35_H_45_N_3_O_7_: C, 67.83; H, 7.32; N, 6.78. Found: C, 67.80; H, 7.38; N, 6.75. MS (APCI) *m*/*z* (I_rel_, %): 620.5 [M+H]^+^ (100) (calculated for C_35_H_46_N_3_O_7_, 620.33). IR (ν max, cm^−1^): 755 (Ar), 1696 (C=O), 2865 (CH), 3380 (NH).

Methyl 12-isopropyl-2-(2-(2-((4-methoxybenzoyl)oxy)acetamido)ethyl)-6,9a-dimethyl-1,3-dioxo-1,2,3,3a,4,5,5a,6,7,8,9,9a,9b,10,11,11a-hexadecahydro-3b,11-ethenonaphtho[2,1-e]isoindole-6-carboxylate **7**. Yield: 513 mg (79%), mp 77 °C, [α]_20_^D^ −22 (c 0.05, CHCl_3_). ^1^H NMR (CDCl_3_, 500 MHz) δ: 0.50 (s, 3H, CH_3_-20); 0.73–0.99 (m, 1H, CH_2_-1_ax_); 0.82 (d, J 6.8, 3H, CH_3_-16); 0.84 (d, J 7.0, 3H, CH_3_-17); 1.07 (s, 3H, CH_3_-19); 1.00–1.85 (m, 12H, CH_2_-1_eq_, 2, 3, 6, 7_ax_, 11, CH-5, 9); 2.10–2.15 (m, 1H, CH-15); 2.50 (d, J 8.5, 1H, CH-21); 2.51 (dt, _1_J 3.0, _2_J 14.0, 1H, CH_2_-7_eq_); 2.85 (dd, _1_J 3.0, _2_J 8.5, 1H, CH-22); 3.04 (s, 1H, CH-12); 3.30–3.49 (m, 2H, CH_2_-4′); 3.65 (s, 3H, CH_3_-25); 3.72 (d, J 6.7, 2H, CH_2_-2′); 3.82 (s, 3H, CH_3_-10′); 3.92–4.10 (m, 2H, CH_2_-1′); 5.35 (s, 1H, CH-14); 6.95–7.00 (m, 2H, CH-8′,8″); 7.15 (br.s., 1H, NH); 7.40–7.47 (m, 2H, CH-7′,7″). ^13^C NMR (CDCl_3_, 125.5 MHz) δ: 15.6 (C-20); 16.8 (C-19); 17.1 (C-2); 20.8 (C-16); 21.8 (C-17); 25.0 (C-6); 27.6 (C-11); 31.1 (C-12); 35.3 (C-15); 35.7 (C-7); 36.7 (C-3); 37.6 (C-1); 37.7 (C-10); 38.1 (C-8); 38.7 (C-21); 38.7 (C-1′); 40.7 (C-4); 47.1 (C-5); 49.5 (C-2′); 51.9 (C-22); 52.4 (C-9); 54.0 (C-25), 55.3 (C-10′); 55.4 (C-4′), 113.7 (C-8′,C-8″); 124.5 (C-14); 128.6 (C-7′, C-7″); 128.8 (C-6′); 147.1 (C-13); 160.7 (C-9′); 170.6 (C-5′); 172.8 (C-3′); 177.9 (C-24); 178.7 (C-23); 179.2 (C-18). Analysis calculated for C_37_H_48_N_2_O_8_: C, 68.50; H, 7.46; N, 4.32. Found: C, 68.60; H, 7.48; N, 4.35. MS (APCI) *m*/*z* (I_rel_, %): 648.3 [M+H]^+^ (100) (calculated for C_37_H_49_N_2_O_8_, 649.4). IR (ν max, cm^−1^): 750 (Ar), 1710 (C=O), 2865 (CH), 3385 (NH).

Synthesis of compounds **8**–**15**. General Procedure. Paraformaldehyde (1 equiv.) was added to a stirred solution of cyclohexylamine (0.12 mL, 1 mmol) or benzylamine (0.11 mL, 1 mmol) in MeOH (25 mL). The resulting mixture was stirred at r.t. for 1 h, and then the carboxylic acid ((0.20 mL, 1 mmol) 2-furoic acid for compounds **8**, **12** or (122 mg, 1 mmol) benzoic acid for compounds **9**, **13** or (123 mg, 1 mmol) nicotinic acid for compounds **10**, **14** or (152 mg, 1 mmol) *p*-methoxy-benzoic acid for compounds **11**, **15**) and the diterpene isocyanide **3** (466 mg, 1 mmol) were added. The reaction mixture was kept at room temperature for 3–5 days (TLC control). After completion of the reaction, the mixture was poured into acidified water, and the precipitate was filtered off, dried and purified by column chromatography.

Methyl 2-(2-(2-(N-cyclohexylfuran-2-carboxamido)acetamido)ethyl)-12-isopropyl-6,9a-dimethyl-1,3-dioxo-1,2,3,3a,4,5,5a,6,7,8,9,9a,9b,10,11,11a-hexadecahydro-3b,11-ethenonaphtho[2,1-e]isoindole-6-carboxylate **8**. Yield: 600 mg (87%), mp 47 °C, [α]_20_^D^ −39 (c 0.05, CHCl_3_). ^1^H NMR (CDCl_3_, 500 MHz) δ: 0.50 (s, 3H, CH_3_-20); 0.73–0.99 (m, 6H, CH_2_-1_ax_, 7′_ax_, 8′_ax_, 7″_ax_, 8″_ax_, 9′_ax_); 0.82 (d, J 6.8, 3H, CH_3_-16); 0.84 (d, J 7.0, 3H, CH_3_-17); 1.07 (s, 3H, CH_3_-19); 1.09–1.85 (m, 12H, CH_2_-1_eq_, 2, 3, 6, 7_ax_, 11, CH-5, 9); 2.00 (s, 5H, CH_2_-7′_eq_, 8′_eq_, 7″_eq_, 8″_eq_, 9′_eq_); 2.10–2.15 (m, 1H, CH-15); 2.50 (d, J 8.5, 1H, CH-21); 2.51 (dt, _1_J 3.1, _2_J 14.0, 1H, CH_2_-7_eq_); 2.85 (dd, _1_J 3.0, _2_J 8.5, 1H, CH-22); 3.04 (s, 1H, CH-12); 3.30–3.49 (m, 3H, CH-6′, CH_2_-4′); 3.65 (s, 3H, CH_3_-25); 3.72 (d, J 6.7, 2H, CH_2_-2′); 3.92 (br.s., 2H, CH_2_-1′); 5.35 (s, 1H, CH-14); 6.49 (br.s., 1H, CH-12′); 6.98 (s, 1H, CH-11′); 7.05 (br.s., 1H, NH); 7.49 (br.s., 1H, CH-13′). ^13^C NMR (CDCl_3_, 125.5 MHz) δ: 15.5 (C-20); 16.7 (C-19); 16.9 (C-2); 18.8 (C-7′, C-7″, C-8′, C-8″), 20.0 (C-16); 20.7 (C-17); 21.7 (C-6); 25.6 (C-9′), 27.5 (C-11); 32.6 (C-12); 35.1 (C-15); 35.5 (C-7); 36.6 (C-3); 37.5 (C-1); 37.6 (C-10); 38.1 (C-8); 40.5 (C-21); 44.9 (C-1′); 47.0 (C-4); 47.4 (C-6′), 49.4 (C-5); 51.8 (C-25); 52.2 (C-22); 54.0 (C-9); 57.9 (C-2′), 70.5 (C-4′), 111.3 (C-12′); 116.9 (C-11′); 124.4 (C-14); 144.1 (C-13′); 147.0 (C-10′); 147.5 (C-13); 170.4 (C-5’); 171.0 (C-3′); 177.6 (C-24); 178.5 (C-23); 179.0 (C-18). Analysis calculated for C_40_H_55_N_3_O_7_: C, 69.64; H, 8.04; N, 6.09. Found: C, 69.60; H, 8.08; N, 6.12. MS (APCI) *m*/*z* (I_rel_, %): 690.5 [M+H]^+^ (100) (calculated for C_40_H_56_N_3_O_7_, 690.89). IR (ν max, cm^−1^): 755 (Ar), 1696, 1771 (C=O), 2960 (CH), 3320–3450 (NH).

Methyl 2-(2-(2-(N-cyclohexylbenzamido)acetamido)ethyl)-12-isopropyl-6,9a-dimethyl-1,3-dioxo-1,2,3,3a,4,5,5a,6,7,8,9,9a,9b,10,11,11a-hexadecahydro-3b,11-ethenonaphtho[2,1-e]isoindole-6-carboxylate **9**. Yield: 574 mg (82%), mp 55 °C, [α]_20_^D^ −19 (c 0.05, CHCl_3_). ^1^H NMR (CDCl_3_, 500 MHz) δ: 0.50 (s, 3H, CH_3_-20); 0.73–0.99 (m, 6H, CH_2_-1_ax_, 7′_ax_, 8′_ax_, 7″_ax_, 8″_ax_, 9′_ax_); 0.82 (d, J 6.8, 3H, CH_3_-16); 0.84 (d, J 7.0, 3H, CH_3_-17); 1.07 (s, 3H, CH_3_-19); 1.00–1.85 (m, 12H, CH_2_-1_eq_, 2, 3, 6, 7_ax_, 11, CH-5, 9); 2.00 (s, 5H, CH_2_-7′_eq_, 8′_eq_, 7″_eq_, 8″_eq_, 9′_eq_); 2.10–2.15 (m, 1H, CH-15); 2.50 (d, J 8.5, 1H, CH-21); 2.51 (dt, _1_J 3.0, _2_J 14.0, 1H, CH_2_-7_eq_); 2.85 (dd, _1_J 3.0, _2_J 8.5, 1H, CH-22); 3.04 (s, 1H, CH-12); 3.30–3.49 (m, 3H, CH-6′, CH_2_-4′); 3.65 (s, 3H, CH_3_-25); 3.72 (d, J 6.7, 2H, CH_2_-2′); 3.92–4.10 (m, 2H, CH_2_-1′); 5.41 (s, 1H, CH-14); 7.05 (br.s., 1H, NH); 7.30–7.51 (m, 5H, CH-11′,11″, 12′, 12″,13′). ^13^C NMR (CDCl_3_, 125.5 MHz) δ: 15.6 (C-20); 16.7 (C-19); 17.0 (C-2); 19.0 (C-7′, C-7″), 20.1 (C-16); 20.7 (C-17); 21.8 (C-6); 24.9 (C-9′), 25.5 (C-8′), 27.6 (C-11); 31.0 (C-12); 32.7 (C-8′’), 35.3 (C-15); 35.6 (C-7); 36.6 (C-3); 37.6 (C-1); 38.1 (C-10); 38.7 (C-8); 40.6 (C-21); 45.0 (C-1′); 46.2 (C-4); 47. (C-6′), 49.43 (C-5); 51.9 (C-25); 52.3 (C-22); 54.0 (C-9); 59.2 (C-2′), 70.6 (C-4’), 124.4 (C-14); 126.5 (C-12′, C-12″); 128.8 (C-11′, C-11″); 129.5 (C-10′); 130.8 (C-13′); 147.1 (C-13); 169.2 (C-5′); 172.8 (C-3′); 177.9 (C-24); 178.7 (C-23); 179.1 (C-18). Analysis calculated for C_42_H_57_N_3_O_6_: C, 72.07; H, 8.21; N, 6.00. Found: C, 72.02; H, 8.20; N, 5.96. MS (APCI) *m*/*z* (I_rel_, %): 700.5 [M+H]^+^ (100) (calculated for C_42_H_58_N_3_O_6_, 700.93). IR (ν max, cm^−1^): 755 (Ar), 1696, 1771 (C=O), 2960 (CH), 3320–3450 (NH).

Methyl 2-(2-(2-(N-cyclohexylnicotinamido)acetamido)ethyl)-12-isopropyl-6,9a-dimethyl-1,3-dioxo-1,2,3,3a,4,5,5a,6,7,8,9,9a,9b,10,11,11a-hexadecahydro-3b,11-ethenonaphtho[2,1-e]isoindole-6-carboxylate **10**. Yield: 518 mg (74%), mp 59 °C, [α]_20_^D^ −26 (c 0.05, CHCl_3_). ^1^H NMR (CDCl_3_, 500 MHz) δ: 0.50 (s, 3H, CH_3_-20); 0.82 (d, J 6.8, 3H, CH_3_-16); 0.84 (d, J 7.0, 3H, CH_3_-17); 0.73–0.99 (m, 6H, CH_2_-1_ax_, 7′_ax_, 8′_ax_, 7″_ax_, 8″_ax_, 9′_ax_); 1.07 (s, 3H, CH_3_-19); 1.00–1.85 (m, 17H, CH_2_-1_eq_, 2, 3, 6, 7_ax_, 11, CH-5, 9, 5H, CH_2_-7′_eq_, 8′_eq_, 7″_eq_, 8″_eq_, 9′_eq_); 2.10–2.15 (m, 1H, CH-15); 2.50 (d, J 8.5, 1H, CH-21); 2.51 (dt, _1_J 3.0, _2_J 14.0, 1H, CH_2_-7_eq_); 2.85 (dd, _1_J 3.0, _2_J 8.5, 1H, CH-22); 3.02 (s, 1H, CH-12); 3.30–3.42 (m, 3H, CH-6′, CH_2_-4′); 3.49–3.50 (br.s., 2H, CH_2_-2′); 3.65 (s, 3H, CH_3_-25); 3.92–4.10 (m, 2H, CH_2_-1′); 5.35 (s, 1H, CH-14); 6.95 (br.s., 1H, NH); 7.30 (br.s., 1H, CH-12′); 7.79–7.81 (m, 1H, CH-11″); 8.80 (br.s., 1H, CH-13′); 8.90 (br.s., 1H, CH-11′). ^13^C NMR (CDCl_3_, 125.5 MHz) δ: 15.6 (C-20); 16.7 (C-19); 17.0 (C-2); 20.1 (C-16); 20.7 (C-17); 21.7 (C-6); 24.8 (C-9′), 25.4 (C-8′, C-8″), 27.6 (C-11); 31.1 (C-7′,C-7″); 32.7 (C-15), 35.2 (C-7); 35.6 (C-12); 36.7 (C-3); 37.6 (C-10, C-6′); 38.1(C-1); 39.1 (C-4′); 40.6 (C-8); 45.0 (C-4); 46.1 (C-1′); 47.1 (C-5), 49.4 (C-22); 51.9 (C-25); 52.4 (C-21); 53.9 (C-9); 59.4 (C-2′), 123.3 (C-12′); 124.4 (C-14); 132.1 (C-10′); 134.6 (C-11″); 147.1 (C-13); 147.5 (C-13′); 150.6 (C-11′); 169.5 (C-5′); 170.1 (C-3′); 178.0 (C-24); 178.8 (C-23); 179.1 (C-18). Analysis calculated for C_41_H_56_N_4_O_6_: C, 70.26; H, 8.05; N, 7.99. Found: C, 70.20; H, 8.00; N, 7.96. MS (APCI) *m*/*z* (I_rel_, %): 701.5 [M+H]^+^ (100) (calculated for C_41_H_57_N_4_O_6_, 701.42). IR (ν max, cm^−1^): 755 (Ar), 1771 (C=O), 2960 (CH), 3320–3450 (NH).

Methyl 2-(2-(2-(N-cyclohexyl-4-methoxybenzamido)acetamido)ethyl)-12-isopropyl-6,9a-dimethyl-1,3-dioxo-1,2,3,3a,4,5,5a,6,7,8,9,9a,9b,10,11,11a-hexadecahydro-3b,11-ethenonaphtho[2,1-e]isoindole-6-carboxylate **11**. Yield: 628 mg (86%), mp 65 °C, [α]_20_^D^ −11 (c 0.05, CHCl_3_). ^1^H NMR (CDCl_3_, 500 MHz) δ: 0.50 (s, 3H, CH_3_-20); 0.73–0.99 (m, 6H, CH_2_-1_ax_, 7′_ax_, 8′_ax_, 7″_ax_, 8″_ax_, 9′_ax_); 0.82 (d, J 6.8, 3H, CH_3_-16); 0.84 (d, J 7.0, 3H, CH_3_-17); 1.07 (s, 3H, CH_3_-19); 1.00–1.85 (m, 12H, CH_2_-1_eq_, 2, 3, 6, 7_ax_, 11, CH-5, 9); 2.00 (s, 5H, CH_2_-7′_eq_, 8′_eq_, 7″_eq_, 8″_eq_, 9′_eq_); 2.10–2.15 (m, 1H, CH-15); 2.50 (d, J 8.5, 1H, CH-21); 2.51 (dt, _1_J 3.0, _2_J 14.0, 1H, CH_2_-7_eq_); 2.85 (dd, _1_J 3.0, _2_J 8.5, 1H, CH-22); 3.04 (s, 1H, CH-12); 3.30–3.49 (m, 3H, CH-6′, CH_2_-4′); 3.65 (s, 3H, CH_3_-25); 3.72 (d, J 6.7, 2H, CH_2_-2′); 3.82 (s, 3H, CH_3_-14′); 3.92–4.10 (M, 2H, CH_2_-1′); 5.35 (s, 1H, CH-14); 6.95–7.00 (m, 2H, CH-12′,12″); 7.15 (br.s., 1H, NH); 7.40–7.47 (m, 2H, CH-11′,11″). ^13^C NMR (CDCl_3_, 125.5 MHz) δ: 15.6 (C-20); 16.8 (C-19); 17.1 (C-2); 20.1 (C-7′, C-7″), 20.8 (C-16); 21.8 (C-17); 25.0 (C-6); 25.6 (C-9′), 25.7 (C-8′), 27.6 (C-11); 31.1 (C-12); 32.7 (C-8″), 35.3 (C-15); 35.7 (C-7); 36.7 (C-3); 37.6 (C-1); 37.7 (C-10); 38.1 (C-8); 38.7 (C-21); 38.8 (C-1′); 40.7 (C-4); 45.0 (C-6′), 47.1 (C-5); 49.5 (C-2′); 51.9 (C-22); 52.4 (C-9); 54.0 (C-25), 55.3 (C-14′); 55.4 (C-4′), 113.7 (C-12′); 113.6 (C-12″); 124.5 (C-14); 128.6 (C-11′, C-11″); 128.8 (C-10′); 147.1 (C-13); 160.7 (C-13′); 170.6 (C-5′); 172.8 (C-3′); 177.9 (C-24); 178.7 (C-23); 179.2 (C-18). Analysis calculated for C_43_H_59_N_3_O_7_: C, 70.75; H, 8.15; N, 5.76. Found: C, 70.80; H, 8.18; N, 5.75. MS (APCI) *m*/*z* (I_rel_, %): 730.5 [M+H]^+^ (100) (calculated for C_43_H_60_N_3_O_7_, 730.4). IR (ν max, cm^−1^): 815 (Ar), 1520, 1771 (C=O), 2865 (CH), 3320–3450 (NH).

Methyl 2-(2-(2-(N-benzylfuran-2-carboxamido)acetamido)ethyl)-12-isopropyl-6,9a-dimethyl-1,3-dioxo-1,2,3,3a,4,5,5a,6,7,8,9,9a,9b,10,11,11a-hexadecahydro-3b,11-ethenonaphtho[2,1-e]isoindole-6-carboxylate **1**.. Yield: 572 mg (82%), mp 67 °C, [α]_20_^D^ −52 (c 0.05, CHCl_3_). ^1^H NMR (CDCl_3_, 500 MHz) δ: 0.50 (s, 3H, CH_3_-20); 0.73–0.99 (m, 1H, CH_2_-1_ax_); 0.82 (d, J 6.8, 3H, CH_3_-16); 0.84 (d, J 7.0, 3H, CH_3_-17); 1.07 (s, 3H, CH_3_-19); 1.09–1.85 (m, 12H, CH_2_-1_eq_, 2, 3, 6, 7_ax_, 11, CH-5, 9); 2.10–2.15 (m, 1H, CH-15); 2.50 (d, J 8.5, 1H, CH-21); 2.51 (dt, _1_J 3.0, _2_J 14.1, 1H, CH_2_-7_eq_); 2.85 (dd, _1_J 3.0, _2_J 8.5, 1H, CH-22); 3.04 (s, 1H, CH-12); 3.30–3.49 (m, 2H, CH_2_-1′); 3.65 (s, 3H, CH_3_-25); 3.72–3.80 (m, 4H, CH_2_-2′,4′); 3.92 (br.s., 2H, CH_2_-6′); 5.35 (s, 1H, CH-14); 6.49 (br.s., 1H, CH-13′); 6.95 (br.s., 1H, NH); 7.05 (s, 1H, CH-12′); 7.20–7.30 (m, 5H, CH-8′, 8″, 9′, 9″, 10′); 7.49 (br.s., 1H, CH-14′). ^13^C NMR (CDCl_3_, 125.5 MHz) δ: 15.6 (C-20); 16.8 (C-19); 17.1 (C-2); 19.1 (C-16); 20.1 (C-17); 20.8 (C-6); 27.6 (C-11); 32.7 (C-12); 35.2 (C-15); 35.6 (C-7); 36.7 (C-3); 37.7 (C-1); 38.2 (C-10); 38.6 (C-8); 40.6 (C-21); 45.0 (C-1′); 47.2 (C-4); 47.4 (C-6′), 49.5 (C-5); 52.0 (C-25); 52.4 (C-22); 53.0 (C-9); 54.0 (C-2′), 70.6 (C-4′), 111.6 (C-13′); 117.5 (C-12′); 124.5 (C-14); 127.2 (C-Ar); 127.8 (C-Ar); 128.5 (C-Ar); 128.6 (C-Ar); 128.9 (C-Ar); 132.0 (C-14′); 136.2 (C-Ar); 144.7 (C-11′); 147.1 (C-13); 161.2 (C-5′); 168.8 (C-3′); 177.9 (C-24); 178.8 (C-23); 179.2 (C-18). Analysis calculated for C_41_H_51_N_3_O_7_: C, 70.56; H, 7.37; N, 6.02. Found: C, 70.60; H, 7.40; N, 6.00. MS (APCI) *m*/*z* (I_rel_, %): 697.4 [M+H]^+^ (100) (calculated for C_41_H_52_N_3_O_7_, 698.4). IR (ν max, cm^−1^): 755 (Ar), 1520, 1771 (C=O), 2960 (CH), 3320–3450 (NH).

Methyl 2-(2-(2-(N-benzylbenzamido)acetamido)ethyl)-12-isopropyl-6,9a-dimethyl-1,3-dioxo-1,2,3,3a,4,5,5a,6,7,8,9,9a,9b,10,11,11a-hexadecahydro-3b,11-ethenonaphtho[2,1-e]isoindole-6-carboxylate **13**. Yield: 595 mg (84%), mp 58 °C, [α]_20_^D^ −9 (c 0.05, CHCl_3_). ^1^H NMR (CDCl_3_, 500 MHz) δ: 0.50 (s, 3H, CH_3_-20); 0.73–0.99 (m, 1H, CH_2_-1_ax_); 0.82 (d, J 6.8, 3H, CH_3_-16); 0.84 (d, J 7.0, 3H, CH_3_-17); 1.07 (s, 3H, CH_3_-19); 1.00–1.85 (m, 12H, CH_2_-1_eq_, 2, 3, 6, 7_ax_, 11, CH-5, 9); 2.10–2.15 (m, 1H, CH-15); 2.50 (d, J 8.5, 1H, CH-21); 2.51 (dt, _1_J 3.0, _2_J 14.0, 1H, CH_2_-7_eq_); 2.85 (dd, _1_J 3.0, _2_J 8.5, 1H, CH-22); 3.04 (s, 1H, CH-12); 3.49 (br.s., 2H, CH-2′); 3.65 (br.s., 2H, CH_2_-1′); 3.65 (s, 3H, CH_3_-25); 3.95–4.18 (m, 2H, CH_2_-4′); 4.65 (br.s, 2H, CH_2_-6′); 5.41 (s, 1H, CH-14); 6.75 (br.s., 1H, NH); 7.11–7.49 (m, 5H, CH-Ar); 7.50–7.71 (m, 5H, CH-Ar). ^13^C NMR (CDCl_3_, 125.5 MHz) δ: 15.6 (C-20); 16.8 (C-19); 17.0 (C-2); 20.1 (C-16); 20.8 (C-17); 21.7 (C-6); 27.6 (C-11); 28.9 (C-12); 29.7 (C-6′); 30.4 (C-15); 32.7 (C-7); 35. (C-3); 36.70 (C-1); 37.7 (C-10); 38.1 (C-8); 40.7 (C-21); 45.0 (C-1′); 47.2 (C-4); 48.7 (C-2′), 49.5 (C-5); 52.90 (C-25); 52.4 (C-22); 54.0 (C-9); 68.2 (C-4′), 124.5 (C-14); 127.2 (C-Ar); 128.5 (C-Ar); 128.6 (C-Ar); 128.9 (C-Ar); 129.5 (C-Ar); 130.1 (C-Ar); 130.9 (C-Ar); 132.0 (C-Ar); 132.1 (C-Ar); 132.2 (C-Ar); 135.2 (C-Ar); 135.9 (C-Ar); 147.2 (C-13); 168.7 (C-5′); 172.8 (C-3′); 178.1 (C-24); 178.9 (C-23); 179.2 (C-18). Analysis calculated for C_43_H_53_N_3_O_6_: C, 72.96; H, 7.55; N, 5.94. Found: C, 73.00; H, 7.50; N, 5.96. MS (APCI) *m*/*z* (I_rel_, %): 707.4 [M+H]^+^ (100) (calculated for C_43_H_54_N_3_O_6_, 708.4). IR (ν max, cm^−1^): 755 (Ar), 1770 (C=O), 2865 (CH), 3320–3450 (NH).

Methyl 2-(2-(2-(N-benzylnicotinamido)acetamido)ethyl)-12-isopropyl-6,9a-dimethyl-1,3-dioxo-1,2,3,3a,4,5,5a,6,7,8,9,9a,9b,10,11,11a-hexadecahydro-3b,11-ethenonaphtho[2,1-e]isoindole-6-carboxylate **1**. Yield: 567 mg (80%), mp 61 °C, [α]_20_^D^ −14 (c 0.05, CHCl_3_). ^1^H NMR (CDCl_3_, 500 MHz) δ: 0.50 (s, 3H, CH_3_-20); 0.82 (d, J 6.8, 3H, CH_3_-16); 0.84 (d, J 7.0, 3H, CH_3_-17); 0.73–0.99 (m, 1H, CH_2_-1_ax_); 1.07 (s, 3H, CH_3_-19); 1.00–1.85 (m, 12H, CH_2_-1_eq_, 2, 3, 6, 7_ax_, 11, CH-5, 9); 2.10–2.15 (m, 1H, CH-15); 2.50 (d, J 8.5, 1H, CH-21); 2.51 (dt, _1_J 3.0, _2_J 14.0, 1H, CH_2_-7_eq_); 2.85 (dd, _1_J 3.0, _2_J 8.5, 1H, CH-22); 3.02 (s, 1H, CH-12); 3.30–3.42 (br.s., 2H, CH_2_-1′); 3.49–3.50 (br.s., 2H, CH_2_-2′); 3.65 (s, 3H, CH_3_-25); 4.00 (s, 2H, CH_2_-4′); 4.60 (s, 2H, CH_2_-6′); 5.40 (s, 1H, CH-14); 6.75 (br.s., 1H, NH); 7.15 (br.s., 1H, CH-Ar); 7.19–7.81 (m, 6H, CH-Ar); 8.80 (br.s., 1H, CH-Ar); 8.90 (br.s., 1H, CH-Ar). ^13^C NMR (CDCl_3_, 125.5 MHz) δ: 15.6 (C-20); 15.7 (C-19); 16.8 (C-2); 17.1 (C-16); 20.1 (C-17); 21.8 (C-6); 27.6 (C-11); 32.7 (C-15), 35.2 (C-7); 35.7 (C-12); 36.7 (C-3); 37.6 (C-10); 37.7 (C-6′); 38.1 (C-1); 39.3 (C-4′); 40.8 (C-8); 45.0 (C-4); 47.2 (C-1′); 48.8 (C-5), 49.5 (C-22); 52.0 (C-25); 52.4 (C-21); 54.0 (C-9); 54.1 (C-2′), 123.3 (C-Ar); 124.4 (C-14); 124.5 (C-Ar); 127.0 (C-Ar); 128.1 (C-Ar); 128.8 (C-Ar), 129.1 (C-Ar); 132.1 (C-Ar); 135.1 (C-Ar); 147.2 (C-13); 148.1 (C-Ar); 151.1 (C-Ar); 161.4 (C-Ar); 168.4 (C-5′); 172.0 (C-3′); 178.0 (C-24); 178.1 (C-23); 179.2 (C-18). Analysis calculated for C_42_H_52_N_4_O_6_: C, 71.16; H, 7.39; N, 7.90. Found: C, 71.20; H, 7.40; N, 7.96. MS (APCI) *m*/*z* (I_rel_, %): 708.4 [M+H]^+^ (100) (calculated for C_42_H_53_N_4_O_6_, 709.4). IR (ν max, cm^−1^): 755 (Ar), 1768 (C=O), 2960 (CH), 3320 (NH).

Methyl 2-(2-(2-(N-benzyl-4-methoxybenzamido)acetamido)ethyl)-12-isopropyl-6,9a-dimethyl-1,3-dioxo-1,2,3,3a,4,5,5a,6,7,8,9,9a,9b,10,11,11a-hexadecahydro-3b,11-ethenonaphtho[2,1-e]isoindole-6-carboxylate **15**. Yield: 598 mg (81%), mp 85 °C, [α]_20_^D^ −11 (c 0.05, CHCl_3_). ^1^H NMR (CDCl_3_, 500 MHz) δ: 0.50 (s, 3H, CH_3_-20); 0.73–0.99 (m, 1H, CH_2_-1_ax_); 0.82 (d, J 6.8, 3H, CH_3_-16); 0.84 (d, J 7.0, 3H, CH_3_-17); 1.07 (s, 3H, CH_3_-19); 1.00–1.85 (m, 12H, CH_2_-1_eq_, 2, 3, 6, 7_ax_, 11, CH-5, 9); 2.10–2.15 (m, 1H, CH-15); 2.50 (d, J 8.5, 1H, CH-21); 2.51 (dt, _1_J 3.0, _2_J 14.0, 1H, CH_2_-7_eq_); 2.85 (dd, _1_J 3.0, _2_J 8.5, 1H, CH-22); 3.04 (s, 1H, CH-12); 3.30–3.49 (m, 4H, CH_2_-6′, CH_2_-4′); 3.65 (s, 3H, CH_3_-25); 3.72 (s, 2H, CH_2_-2′); 3.82 (s, 3H, CH_3_-14′); 4.70 (s, 2H, CH_2_-1′); 5.45 (s, 1H, CH-14); 6.85 (br.s., 1H, NH); 6.95–7.00 (m, 2H, CH-Ar); 7.10–7.60 (m, 6H, CH-Ar); 8.10–8.20 (m, 2H, CH-Ar). ^13^C NMR (CDCl_3_, 125.5 MHz) δ: 15.7 (C-20); 16.9 (C-19); 17.2 (C-2); 20.2 (C-16); 20.8 (C-17); 21.9 (C-6); 27.7 (C-11); 32.8 (C-12); 35.4 (C-15); 35.8 (C-7); 36.8 (C-3); 37.7 (C-1); 37.8 (C-10); 38.0 (C-8); 38.2 (C-21); 39.1 (C-1′); 40.8 (C-4); 45.2 (C-6′), 47.3 (C-5); 49.6 (C-2′); 52.1 (C-22); 52.2 (C-9); 54.1 (C-25), 54.2 (CH_3_); 55.5 (C-4′), 113.8 (2C-Ar); 122.0 (C-Ar); 124.6 (C-14); 127.2 (C-Ar); 127.9 (C-Ar); 128.6 (C-Ar); 129.0 (C-Ar); 129.4 (C-Ar); 132.3 (2C-Ar); 136.3 (C-Ar); 147.4 (C-13); 161.5 (C-Ar); 164.0 (C-5′); 170.9 (C-3′); 178.2 (C-24); 179.0 (C-23); 179.3 (C-18). Analysis calculated for C_44_H_55_N_3_O_7_: C, 71.62; H, 7.51; N, 5.69. Found: C, 71.60; H, 7.50; N, 5.70. MS (APCI) *m*/*z* (I_rel_, %): 737.4 [M+H]^+^ (100) (calculated for C_44_H_56_N_3_O_7_, 738.4). IR (ν max, cm^−1^): 750 (Ar), 1520, 1771 (C=O), 2960 (CH), 3320 (NH).

Synthesis of compounds **16**–**18**. General Procedure. Diterpene isocyanide **3** (466 mg, 1 mmol), paraformaldehyde (1 equiv.) and TMSN_3_ (0.21 mL, 1.8 mmol) were added to a stirred solution of morpholine (0.10 mL, 1 mmol) for compound **16** or cyclohexylamine (0.12 mL, 1 mmol) for compound **17** or benzylamine (0.11 mL, 1 mmol) for compound **18** in MeOH (25 mL). The resulting mixture was stirred at r.t. for 2–3 days (TLC control). After completion of the reaction, the mixture was poured into acidified water, and the precipitate was filtered off, dried and purified by column chromatography.

Methyl 2-(2-(5-(morpholinomethyl)-2H-tetrazol-2-yl)ethyl)-12-isopropyl-6,9a-dimethyl-1,3-dioxo-1,2,3,3a,4,5,5a,6,7,8,9,9a,9b,10,11,11a-hexadecahydro-3b,11-ethenonaphtho[2,1-e]isoindole-6-carboxylate **16**. Yield: 487 mg (80%), mp 150 °C, [α]_20_^D^ −29 (c 0.05, CHCl_3_). ^1^H NMR (CDCl_3_, 500 MHz) δ: 0.50 (s, 3H, CH_3_-20); 0.73–0.82 (m, 1H, CH_2_-1_ax_); 0.82 (d, J 6.8, 3H, CH_3_-16); 0.84 (d, J 7.0, 3H, CH_3_-17); 1.10 (s, 3H, CH_3_-19); 1.00–1.85 (m, 12H, CH_2_-1_eq_, 2, 3, 6, 7_ax_, 11, CH-5, 9); 2.10–2.15 (m, 1H, CH-15); 2.42–2.51 (m, 6H, CH-21, CH_2_-7_eq_, 5′,5″); 2.85 (dd, _1_J 3.0, _2_J 8.5, 1H, CH-22); 3.04 (s, 1H, CH-12); 3.61–3.65 (m, 4H, CH_2_-6′,6″); 3.65 (s, 3H, CH_3_-25); 4.15 (s, 4H, CH_2_-2′,4′); 4.50–4.65 (m, 2H, CH_2_-1’); 5.40 (s, 1H, CH-14). ^13^C NMR (CDCl_3_, 125.5 MHz) δ: 15.6 (C-20); 16.7 (C-19); 16.9 (C-2); 20.2 (C-16); 20.7 (C-17); 21.7 (C-6); 27.5 (C-11); 29.7 (C-12); 32.7 (C-15); 35.2 (C-7); 35.4 (C-3); 36.6 (C-1); 36.8 (C-1′); 37.6 (C-10); 38.1 (C-8); 38.7 (C-4); 40.6 (C-21); 44.5 (C-5); 45.0 (C-25); 52.0 (C-4′); 52.3 (C-22); 53.5 (C-5′,C-5″), 54.1 (C-9); 66.7 (C-6′, C-6″); 68.1 (C-2′), 124.7 (C-14); 147.0 (C-13); 151.7 (C-3′); 176.9 (C-24); 178.1 (C-23); 179.1 (C-18). Analysis calculated for C_33_H_48_N_6_O_5_: C, 65.11; H, 7.95; N, 13.80. Found: C, 65.10; H, 7.98; N, 14.00. MS (APCI) *m*/*z* (I_rel_, %): 609.5 [M+H]^+^ (100) (calculated for C_33_H_49_N_6_O_5_, 609.4). IR (ν max, cm^−1^): 1540 (C=N), 1770 (C=O), 2865 (CH), 3450 (NH).

Methyl 2-(2-(5-((cyclohexylamino)methyl)-2H-tetrazol-2-yl)ethyl)-12-isopropyl-6,9a-dimethyl-1,3-dioxo-1,2,3,3a,4,5,5a,6,7,8,9,9a,9b,10,11,11a-hexadecahydro-3b,11-ethenonaphtho[2,1-e]isoindole-6-carboxylate **17**. Yield: 552 mg (89%), mp 55 °C, [α]_20_^D^ −20 (c 0.05, CHCl_3_). ^1^H NMR (CDCl_3_, 500 MHz) δ: 0.50 (s, 3H, CH_3_-20); 0.73–1.09 (m, 5H, CH_2_-1_ax_, 7′, 7″); 0.82 (d, J 6.8, 3H, CH_3_-16); 0.84 (d, J 7.0, 3H, CH_3_-17); 1.10 (s, 3H, CH_3_-19); 1.00–1.85 (m, 18H, CH_2_-1_eq_, 2, 3, 6, 7_ax_, 11, 6′, 6″, 8′, CH-5, 9); 2.10–2.15 (m, 1H, CH-15); 2.42–2.51 (m, 3H, CH-21, CH_2_-7_eq_, 5′); 2.85 (dd, _1_J 3.0, _2_J 8.5, 1H, CH-22); 3.04 (s, 1H, CH-12); 3.65 (s, 3H, CH_3_-25); 3.79–3.82 (m, 2H, CH_2_-2′); 4.15 (s, 2H, CH_2_-4′); 4.17 (br.s., 1H, NH); 4.50–4.65 (m, 2H, CH_2_-1′); 5.40 (s, 1H, CH-14). ^13^C NMR (CDCl_3_, 125.5 MHz) δ: 15.6 (C-20); 16.8 (C-19); 17.1 (C-2); 19.1 (C-16); 20.2 (C-17); 20.7 (C-6); 24.8 (C-11); 26.0 (C-12); 27.5 (C-7′, C-7″); 32.7 (C-15); 33.3 (C-8′), 35.2 (C-7); 35.4 (C-3); 36.7 (C-1); 36.8 (C-6′, C-6″); 37.7 (C-10); 38.2 (C-8); 38.8 (C-4); 39.8 (C-21); 40.7 (C-5); 44.6 (C-1′); 45.1 (C-25); 52.0 (C-1′); 52.4 (C-22); 54.2 (C-9); 56.7 (C-5′), 68.2 (C-2′), 124.7 (C-14); 147.0 (C-13); 154.0 (C-3′); 177.0 (C-24); 178.2 (C-23); 179.2 (C-18). Analysis calculated for C_35_H_52_N_6_O_4_: C, 67.71; H, 8.44; N, 13.54. Found: C, 67.73; H, 8.48; N, 13.55. MS (APCI) *m*/*z* (I_rel_, %): 621.5 [M+H]^+^ (100) (calculated for C_35_H_53_N_6_O_4_, 622.4). IR (ν max, cm^−1^): 1540 (C=N), 1699, 1770 (C=O), 2865 (CH), 3450 (NH).

Methyl 2-(2-(5-((benzylamino)methyl)-2H-tetrazol-2-yl)ethyl)-12-isopropyl-6,9a-dimethyl-1,3-dioxo-1,2,3,3a,4,5,5a,6,7,8,9,9a,9b,10,11,11a-hexadecahydro-3b,11-ethenonaphtho[2,1-e]isoindole-6-carboxylate **18**. Yield: 522 mg (83%), mp 61 °C, [α]_20_^D^ −38(c 0.05, CHCl_3_). ^1^H NMR (CDCl_3_, 500 MHz) δ: 0.49 (s, 3H, CH_3_-20); 0.73–1.09 (m, 2H, CH_2_-1_ax_,); 0.82 (d, J 6.8, 3H, CH_3_-16); 0.84 (d, J 7.0, 3H, CH_3_-17); 1.10 (s, 3H, CH_3_-19); 1.00–1.85 (m, 12H, CH_2_-1_eq_, 2, 3, 6, 7_ax_, 11, CH-5, 9); 2.10–2.15 (m, 1H, CH-15); 2.42–2.51 (m, 2H, CH-21, CH_2_-7_eq_); 2.85 (dd, _1_J 3.0, _2_J 8.5, 1H, CH-22); 3.04 (s, 1H, CH-12); 3.65 (s, 3H, CH_3_-25); 3.79–3.82 (m, 2H, CH_2_-5′); 4.15 (s, 2H, CH_2_-4′); 4.50–4.65 (m, 4H, CH_2_-1′, CH_2_-2′); 5.45 (s, 1H, CH-14); 7.2–7.4 (m, 5H, H-Ar); 7.57 (br.s., 1H, NH). ^13^C NMR (CDCl_3_, 125.5 MHz) δ: 15.6 (C-20); 16.8 (C-19); 17.1 (C-2); 19.1 (C-16); 20.2 (C-17); 20.7 (C-6); 27.7 (C-11); 29.7 (C-12); 32.7 (C-15); 35.2 (C-7); 35.9 (C-3); 36.7 (C-1); 36.9 (C-10); 37.7 (C-8); 38.2 (C-4); 39.4 (C-21); 45.2 (C-5); 47.2 (C-4′); 49.5 (C-25); 52.3 (C-1′); 52.6 (C-22); 53.4 (C-9); 54.1 (C-5′), 70.6 (C-2′), 124.7 (C-14); 127.5 (C-Ar); 128.4 (C-Ar); 128.5 (C-Ar); 128.7 (C-Ar); 129.2 (C-Ar); 138.8 (C-Ar); 147.0 (C-13); 153.4 (C-3′); 177.0 (C-24); 177.5 (C-23); 179.1 (C-18). Analysis calculated for C_36_H_48_N_6_O_4_: C, 68.76; H, 7.69; N, 13.37. Found: C, 68.73; H, 7.70; N, 13.35. MS (APCI) *m*/*z* (I_rel_, %): 628.4 [M+H]^+^ (100) (calculated for C_36_H_49_N_6_O_4_, 629.4). IR (ν max, cm^−1^): 1540 (C=N), 1770 (C=O), 2960 (CH), 3060 (Ar), 3450 (NH).

Comprehensive ADME and drug-likeness evaluation of synthesized compounds using SwissADME. SwissADME calculations were performed using the freely available web platform SwissADME (http://www.swissadme.ch, accessed on 15 January 2026) to evaluate the physicochemical properties, ADME profile and drug-likeness of the synthesized diterpene derivatives. The following descriptors were obtained: molecular weight, number of hydrogen bond donors and acceptors, number of rotatable bonds, topological polar surface area (TPSA), fraction of sp3-hybridized carbons, and several lipophilicity predictors (iLOGP, XLOGP3, WLOGP, MLOGP, SILICOS-IT), as well as the consensus LogP value. Aqueous solubility was estimated using built-in solubility models (e.g., ESOL and SILICOS-IT), which classify compounds into standard solubility classes. The ADME-related properties were predicted using the gastrointestinal absorption and blood–brain barrier penetration models (including the BOILED-Egg model), as well as support vector machine-based classifiers for P-glycoprotein substrate recognition and cytochrome P450 inhibition. Drug-likeness was assessed according to Lipinski, Ghose, Veber, Egan and Muegge rules, with the number of rule violations recorded for each compound. Medicinal chemistry friendliness was further evaluated using PAINS filters, structural alerts, and the synthetic accessibility score provided by SwissADME.

Targets prediction of newly synthesized products **1**–**18** was performed through Swiss Targets Prediction tools. Molecules’ structures in (smi) format were imported in the panel box and submitted to predict the new biological targets by selecting the organism (homosapiens) humans. The SWISS Targets Prediction Software Swiss target prediction tool was used to predict new biological targets, and new query compound structures were uploaded in SMILES format, as indicated by the red ‘predict targets’ button. Clicking the button initiates the computation process. Chemical structures are processed through JChemWeb services (version 18.29.0) and Open Babel (version 2.4.1), which perform canonicalization, kekulization, and hydrogenation with pH 7.4. These parameters are applied to the SMILES format which is then translated both into FP2 binary vectors and 20 conformers of each molecule which finally generate ES5D vectors. Structure validation of the input molecules was applied through the JChem structure checker to the 2D and 3D screening process to search for similar molecules among the compounds already known to be experimentally active against one or several of the 3068 available protein targets in the database. The process took a few seconds, and the results of the molecule target prediction are displayed on a single panel [50].

Toxicity. Toxicity profiles of the synthesized diterpene derivatives **1**–**18** were predicted using the ProTox-III web server (https://tox-new.charite.de/protox_III/ (accessed on 15 January 2026)), a computational platform for assessing multiple toxicity endpoints through molecular similarity, pharmacophore models, fragment propensities, and machine learning approaches. The server predicts 61 toxicity categories including acute toxicity (LD_50_), organ toxicity (hepatotoxicity, nephrotoxicity, neurotoxicity, cardiotoxicity), carcinogenicity, mutagenicity, cytotoxicity, immunotoxicity, ecotoxicity, clinical toxicity, nutritional toxicity, molecular initiating events (MIE), Tox21 pathways, and off-target toxicity liabilities. Models employ Random Forest classifiers and Deep Neural Networks trained on curated datasets with various data sampling methods (SMOTE, randOS, SMOTEVDM) to achieve balanced sensitivity/specificity, providing predictions with confidence scores, toxicity class probabilities, predicted LD_50_ values, and visualization through toxicity radar and network plots. Standard default settings were used, with molecular structures input as SMILES or drawn directly in the interface [51,52,53].

## 4. Conclusions

In conclusion, we have developed the first strategy for the synthesis of abietane diterpene isocyanide and applied it to the synthesis of novel α-acyloxycarboxamides, α-acylaminocarboxamides and tetrazoles under IMCRs conditions. Taking into account the obtained results (high yields of intermediates, ease of synthesis, availability of reagents), we believe that this approach can also be used for other amine-containing terpenoids and significantly expand the set of structurally diverse peptide-like conjugates obtained by IMCRs. A comprehensive in silico evaluation demonstrated that the synthesized series of diterpene derivatives possess an optimal set of pharmacological properties, indicating their high preclinical potential. A consensus Log Po/w in the range of 3.35–5.68 ensures balanced lipophilicity, while high gastrointestinal absorption of most compounds predetermines the potential for oral administration. Selective inhibition of CYP3A4 without affecting other cytochromes P450 minimizes the risk of drug interactions, while the absence of PAINS alerts guarantees the specificity of the biological action, and low LD_50_ values without hepato- or carcinogenic effects confirm a favorable safety profile. The most promising in this series were the tetrazole derivatives **16**–**18**, while other highly lipophilic analogs can be optimized for solubility by introducing polar functional groups. The presented ADME and drug-likeness characteristics are preliminary theoretical predictions justifying the selection of these compounds as promising candidates. The obtained results indicate that newly abietane isocyanide is a versatile building block for constructing MCR libraries, opening up opportunities for targeted SAR design of diterpene derivatives with improved pharmacological parameters and stimulating further research on solubility optimization and in vitro confirmation of their biological activity. The next stage of research includes experimental validation of in silico predictions, including an ADME panel (solubility, permeability, metabolism) and biological activity tests (in vitro/in vivo).

## Figures and Tables

**Table 1:** Optimization of conditions in the synthesis of diterpene isocyanide **3** from formamide **2**.

№	Reagent	Solvent	Time, h	Yield, %
1	TsCl	Et_3_N	46–48	0
2	POCl_3_	Et_3_N	46–48	5–10
3	POCl_3_	Et_3_N, CH_2_Cl_2_	46–48	5–9
4	POCl_3_	Et_3_N, Py	46–48	5–9
5	I_2_, Ph_3_P	Et_3_N, CH_2_Cl_2_	10–12	60–69
6	I_2_, Ph_3_P	Et_3_N	46–48	30–32

**Table 2:** The bioavailability scores and pharmacokinetics of derivatives **1**–**18** calculated with SwissADME database.

Comp.	GI Abs ^a^	Bioavail. Score ^b^	BBB per ^c^	P-gp Substrate ^d^	CYP1A2 Inh ^e^	CYP2C 19 Inh ^f^	CYP2C9 Inh ^g^	CYP2D6 Inh ^h^	CYP3A4 Inh ^i^
**1**	High	0.55	No	Yes	No	No	No	No	Yes
**2**	High	0.55	No	Yes	No	No	No	No	Yes
**3**	High	0.55	Yes	Yes	No	No	No	No	No
**4**	Low	0.55	No	Yes	No	No	No	No	Yes
**5**	High	0.17	No	Yes	No	No	No	No	Yes
**6**	Low	0.55	No	Yes	No	No	No	No	Yes
**7**	Low	0.55	No	Yes	No	No	No	No	Yes
**8**	Low	0.55	No	Yes	No	No	No	No	Yes
**9**	Low	0.17	No	Yes	No	No	No	No	Yes
**10**	Low	0.55	No	Yes	No	No	No	No	Yes
**11**	Low	0.17	No	Yes	No	No	No	No	Yes
**12**	Low	0.55	No	Yes	No	No	No	No	Yes
**13**	Low	0.17	No	Yes	No	No	No	No	Yes
**14**	Low	0.55	No	Yes	No	No	No	No	Yes
**15**	High	0.17	No	Yes	No	No	No	No	Yes
**16**	High	0.17	No	Yes	No	No	No	No	Yes
**17**	High	0.55	No	Yes	No	No	No	No	Yes
**18**	High	0.55	No	Yes	No	No	No	No	Yes

^a^ Gastrointestinal absorption, ^b^ bioavailability score, ^c^ blood–brain barrier permeability, ^d^ P-gp substrate activity, ^e^ potential inhibition CYP1A2, ^f^ potential inhibition CYP2C, ^g^ potential inhibition CYP2C9, ^h^ potential inhibition CYP2D6, ^i^ potential inhibition CYP3A4.

**Table 3:** Toxicity prediction of synthesized compounds **1**–**18**.

Comp.	LD_50_ (mg/kg)	Toxicity Class	Average Similarity (%)	Prediction Accuracy (%)	H ^a^	Cg ^b^	I ^c^	M ^d^	Ct ^e^
**1**	1000	4	73.73	69.26	−(0.87)	+(0.55)	+(0.80)	−(0.62)	−(0.59)
**2**	2000	4	72.04	69.26	−(0.87)	+(0.54)	+(0.93)	−(0.62)	−(0.61)
**3**	1000	4	67.61	68.07	−(0.88)	+(0.59)	−(0.59)	−(0.62)	−(0.56)
**4**	2000	4	49.11	54.26	−(0.85)	+(0.54)	+(0.87)	−(0.67)	−(0.56)
**5**	3500	5	62.95	68.07	−(0.86)	+(0.52)	+(0.92)	−(0.69)	−(0.54)
**6**	3500	5	62.95	68.07	−(0.87)	+(0.51)	+(0.99)	−(0.66)	−(0.61)
**7**	2000	4	54.27	67.38	−(0.84)	+(0.54)	+(0.98)	−(0.70)	−(0.51)
**8**	3000	5	54.54	67.38	−(0.79)	+(0.52)	+(0.86)	−(0.67)	−(0.59)
**9**	2250	5	61.63	68.07	−(0.83)	+(0.51)	+(0.94)	−(0.68)	−(0.56)
**10**	3500	5	57.77	67.38	−(0.87)	+(0.55)	+(0.80)	−(0.62)	−(0.59)
**11**	4000	5	61.00	68.07	−(0.80)	+(0.53)	+(0.99)	−(0.67)	−(0.55)
**12**	3000	5	54.77	67.38	−(0.84)	+(0.55)	+(0.67)	−(0.67)	−(0.68)
**13**	2250	5	61.53	68.07	−(0.86)	+(0.53)	+(0.73)	−(0.68)	−(0.67)
**14**	2250	5	53,50	67,38	−(0.87)	+(0.53)	+(0.67)	−(0.70)	−(0.72)
**15**	4000	5	60.94	68.07	−(0.83)	+(0.56)	+(0.93)	−(0.69)	−(0.63)
**16**	9500	6	50.59	67.38	−(0.60)	−(0.53)	+(0.91)	−(0.51)	+(0.53)
**17**	10,000	6	48.43	54.26	−(0.65)	−(0.56)	−(0.59)	+(0.51)	+(0.55)
**18**	2000	4	44.64	54.26	−(0.67)	−(0.58)	+(0.63)	+(0.50)	+(0.57)

^a^ Hepatotoxicity; ^b^ carcinogenicity; ^c^ immunotoxicity; ^d^ mutagenicity; ^e^ cytotoxicity; (+) means active or toxic; (−) means inactive or nontoxic. (The numbers in round brackets indicate the probability.)

## Data Availability

The original contributions presented in this study are included in the article/Supplementary Material. Further inquiries can be directed to the corresponding author.

## Supplementary Materials

The following supporting information can be downloaded at: https://www.mdpi.com/article/10.3390/ijms27083494/s1.

## References

[1] Graziano G., Stefanachi A., Contino M., Prieto-Díaz R., Ligresti A., Kumar P., Scilimati A., Sotelo E., Leonetti F. (2023). Multicomponent Reaction-Assisted Drug Discovery: A Time- and Cost-Effective Green Approach Speeding Up Identification and Optimization of Anticancer Drugs. Int. J. Mol. Sci..

[2] Ugi I. (1982). From isocyanides via four component condensations to antibiotic syntheses. Angew. Chem. Int. Ed. Engl..

[3] Ugi I., Domling A., Horl W. (1994). Multicomponent reaction in organic chemistry. Endeavor.

[4] Reza Kazemizadeh A., Ramazani A. (2012). Synthetic Applications of Passerini Reaction. Curr. Org. Chem..

[5] Dömling A., Wang W., Wang K. (2012). Chemistry and biology of multicomponent reactions. Chem. Rev..

[6] Toure B.B., Hall D.G. (2009). Natural product synthesis using multicomponent reaction strategies. Chem. Rev..

[7] Veena K.S., Taniya M.S., Ravindran J., Thangarasu A.K., Priya S., Lankalapalli R.S. (2022). Semi-synthetic diversification of coronarin D, a labdane diterpene, under Ugi reaction conditions. Nat. Prod. Res..

[8] Czollner L., Beseda I., Jordis U., Stanetty C., Amer H., Del Ruiz-Ruiz M.C., Kosma P., Classen-Houben D. (2009). Ugi reactions of tertiary carboxylic acids: Combinatorial synthesis of glycyrrhetinic acid derivatives. Proceedings of the 13th International Electronic Conference on Synthetic Organic Chemistry.

[9] Rodríguez-López F., García-Gutiérrez H.A., Gámez-Montaño R. (2022). Synthesis of Bis-Amides Employing a Plant-Derived Triterpenoid as Component in the Ugi Reaction. Chem. Proc..

[10] Sultani H.N., Morgan I., Hussain H., Roos A.H., Haeri H.H., Kaluđerović G.N., Hinderberger D., Westermann B. (2021). Access to New Cytotoxic Triterpene and Steroidal Acid-TEMPO Conjugates by Ugi Multicomponent-Reactions. Int. J. Mol. Sci..

[11] Wiemann J., Heller L., Csuk R. (2018). An access to a library of novel triterpene derivatives with a promising pharmacological potential by Ugi and Passerini multicomponent reactions. Eur. J. Med. Chem..

[12] Wiemann J., Fischer L., Kessler J., Ströhl D., Csuk R. (2018). Ugi multicomponent-reaction: Syntheses of cytotoxic dehydroabietylamine derivatives. Bioorg. Chem..

[13] Heise N.V., Schmidt A., Schüler J.-A., Csuk R. (2024). Dehydroabietylamine derived bistetrazoles from ultrasound-assisted pseudo-seven-component Ugi reactions act as efficient and selective inhibitors of cholinesterases. Eur. J. Med. Chem. Rep..

[14] Tretyakova E.V., Ma X., Kazakova O.B., Shtro A.A., Petukhova G.D., Smirnova A.A., Xu H., Xiao S. (2023). Abietic, maleopimaric and quinopimaric dipeptide Ugi-4CR derivatives and their potency against influenza A and SARS-CoV-2. Nat. Prod. Res..

[15] Smirnova A.A., Zakirova L.M., Smirnova I.E., Tretyakova E.V. (2023). Synthesis of Novel Diterpenic Peptides via the Ugi Reaction and Their Anticancer Activities. Molbank.

[16] Smirnova A.A., Tretyakova E.V., Kazakova O.B. (2024). Inhibiting the cancer cell growth by maleopimarate amino imide bis-tetrazoles synthesized via the azido-Ugi reaction. Mendeleev Commun..

[17] Tretyakova E., Smirnova A., Babkov D., Kazakova O. (2024). Derivatization of Abietane Acids by Peptide-like Substituents Leads to Submicromolar Cytotoxicity at NCI-60 Panel. Molecules.

[18] Smirnova A., Tretyakova E., Kazakova O. (2024). New Cytotoxic α-Aminoacylamide and bis-1,5- Disubstituted Tetrazole Adducts from Amino-Diterpene Molecules by Ugi Reaction. Chem. Biol. Drug Des..

[19] Liu C., Huang W., Zhang J., Rao Z., Gu Y., Jérôme F. (2021). Formaldehyde in multicomponent reactions. Green Chem..

[20] Bode M.L., Gravestock D., Rousseau A.L. (2016). Synthesis, Reactions and Uses of Isocyanides in Organic Synthesis. An Update. Org. Prep. Proced. Int..

[21] Koodkaew I., Sunohara Y., Matsuyama S., Matsumoto H. (2012). Isolation of ambiguine D isonitrile from *Hapalosiphon* sp. and characterization of its phytotoxic activity. Plant Growth Regul..

[22] Wright A.D., McCluskey A., Robertson M.J., MacGregor K.A., Gordon C.P., Guenther J. (2011). Anti-malarial, anti-algal, anti-tubercular, anti-bacterial, anti-photosynthetic, and anti-fouling activity of diterpene and diterpene isonitriles from the tropical marine sponge *Cymbastela hooperi*. Org. Biomol. Chem..

[23] Miyaoka H., Abe Y., Kawashima E. (2012). Synthesis of Marine Diterpene Isocyanide (−)-Kalihinol Y and Diterpene Isothiocyanate (−)-10-Epi-kalihinol I. Chem. Pharm. Bull..

[24] Allan K.M., Kobayashi K., Rawal V.H. (2012). A Unified Route to the Welwitindolinone Alkaloids: Total Syntheses of (−)-N-Methylwelwitindolinone C Isothiocyanate, (−)-N-Methylwelwitindolinone C Isonitrile, and (−)-3-Hydroxy-N-methylwelwitindolinone C Isothiocyanate. J. Am. Chem. Soc..

[25] Reguera L., Attorresi C.I., Ramírez J.A., Rivera D.G. (2019). Steroid diversification by multicomponent reactions. Beilstein J. Org. Chem..

[26] Wessjohann L.A., Voigt B., Rivera D.G. (2005). Diversity Oriented One-Pot Synthesis of Complex Macrocycles: Very Large Steroid–Peptoid Hybrids from Multiple Multicomponent Reactions Including Bifunctional Building Blocks. Angew. Chem. Int. Ed..

[27] Rivera D.G., León F., Concepción O., Morales F.E., Wessjohann L.A. (2013). A Multiple Multicomponent Approach to Chimeric Peptide–Peptoid Podands. Chem. Eur. J..

[28] Lesma G., Luraghi A., Rainoldi G., Mattiuzzo E., Bortolozzi R., Viola G., Silvani A. (2016). Multicomponent Approach to Bioactive Peptide–Ecdysteroid Conjugates: Creating Diversity at C6 by Means of the Ugi Reaction. Synthesis.

[29] Echemendía R., da Silva G.P., Kawamura M.Y., de la Torre A.F., Corrêa A.G., Ferreira M.A.B., Rivera D.G., Paixão M.W. (2019). A stereoselective sequential organocascade and multicomponent approach for the preparation of tetrahydropyridines and chimeric derivatives. Chem. Commun..

[30] Kitano Y., Chiba K., Tada M. (2001). Highly efficient conversion of alcohols to isocyanides. Synthesis.

[31] Kitano Y., Chiba K., Tada M. (1999). A direct conversion of alkenes to isocyanides. Synlett.

[32] Gassman P.G., Guggenheim T.L. (1982). Opening of epoxides with trimethylsilyl cyanide to produce beta-hydroxy isonitriles. A general synthesis of oxazolines and beta-amino alcohols. J. Am. Chem. Soc..

[33] Hofmann A.W. (1867). Ueber eine neue Reihe von Homologen der Cyanwasserstoffsäure. Justus Liebigs Ann. Chem..

[34] Gautier A. (1867). Ueber die Einwirkung des Chlorwasserstoffs ua auf das Aethyl-und Methylcyanür. Justus Liebigs Ann. Chem..

[35] El Kaim L., Grimaud L., Schiltz A. (2009). “Isocyanide-free” Ugi reactions. Org. Biomol. Chem..

[36] Ugi I., Meyr R. (1958). Neue darstellungsmethode für isonitrile. Angew. Chem..

[37] Josien H., Ko S.B., Bom D., Curran D.P. (1998). A General Synthetic Approach to the (20S)-Camptothecin Family of Antitumor Agents by a Regiocontrolled Cascade Radical Cyclization of Aryl Isonitriles. Chem. Eur. J..

[38] Wang X., Wang Q.G., Luo Q.L. (2015). Synthesis of isonitriles from N-substituted formamides using triphenylphosphine and iodine. Synthesis.

[39] Stephany R.W., de Bie M.J.A., Drenth W. (1974). A ^13^C-NMR and IR study of isocyanides and some of their complexes. Org. Magn. Reson..

[40] SwissAdme Swiss Drug Design. http://www.swissadme.ch.

[41] ProTox 3.0—Prediction. https://tox.charite.de/protox3/index.php?site=home.

[42] Ertl P., Rohde B., Selzer P. (2000). Fast Calculation of Molecular Polar Surface Area as a Sum of Fragment-Based Contributions and Its Application to the Prediction of Drug Transport Properties. J. Med. Chem..

[43] Veber D.F., Johnson S.R., Cheng H.-Y., Smith B.R., Ward K.W., Kopple K.D. (2002). Molecular Properties That Influence the Oral Bioavailability of Drug Candidates. J. Med. Chem..

[44] Pliška V., Testa B., van de Waterbeemd H., Mannhold R., Kubinyi H., Testa B., van de Waterbeemd H., Timmerman H., Pliška V. (1996). Lipophilicity in drug action and toxicology. Methods and Principles in Medicinal Chemistry.

[45] Sitarek P., Kowalczyk T., Synowiec E., Merecz-Sadowska A., Bangay G., Princiotto S., Śliwiński T., Rijo P. (2022). An Evaluation of the Novel Biological Properties of Diterpenes Isolated from *Plectranthus ornatus* Codd. In Vitro and In Silico. Cells.

[46] Pantaleão S.Q., Fernandes P.O., Gonçalves J.E., Maltarollo V.G., Honorio K.M. (2021). Recent Advances in the Prediction of Pharmacokinetics Properties in Drug Design Studies: A Review. Chem. Med. Chem..

[47] Mancini F., Unver M.Y., Elgaher W.A.M., Jumde V.R., Alhayek A., Lukat P., Herrmann J., Witte M.D., Köck M., Blankenfeldt W. (2020). Protein-Templated Hit Identification through an Ugi Four-Component Reaction. Chem. Eur. J..

[48] Hussein M., Varala R., Kamsali M.M.A., Seema V., Beda D.P., Syed M.A., Alam M.M. (2025). Recent Advances in the Chemistry of Tetrazole Derivatives-A Quinquennial Update (Mid-2019 to date). Mini-Rev. Org. Chem..

[49] Khabibullina G.R., Fedotova E.S., Tretyakova E.V., Tyumkina T.V., Parfenova L.V., Ibragimov A.G. (2019). Synthesis of N-substituted thiazacycloalkanes by cyclothiomethylation of primary aliphatic amines and amino derivatives of maleopimaric acid. Russ. J. Gen. Chem..

[50] Daina A., Michielin O., Zoete V. (2017). SwissADME: A free web tool to evaluate pharmacokinetics, drug-likeness and medicinal chemistry friendliness of small molecules. Sci. Rep..

[51] Drwal M.N., Banerjee P., Dunkel M., Wettig M.R., Preissner R. (2014). ProTox: A web server for the in silico prediction of rodent oral toxicity. Nucleic Acids Res..

[52] Banerjee P., Eckert A.O., Schrey A.K., Preissner R. (2018). ProTox-II: A webserver for the prediction of toxicity of chemicals. Nucleic Acids Res..

[53] Banerjee P., Kemmler E., Dunkel M., Preissner R. (2024). ProTox 3.0: A webserver for the prediction of toxicity of chemicals. Nucleic Acids Res..

