# Supplementary material for: A First Diversity-Oriented N-Maleopimarimido-Isocyanide for Multicomponent Reactions: Synthesis, Application, and In Silico Evaluation
Source: Int J Mol Sci. 2026 Apr 14;27(8):3494. doi: 10.3390/ijms27083494 (PMC13116359; doi:10.3390/ijms27083494)

SUPPORTING MATERIALS

A First Diversity Oriented N-Maleopimarimido-Isocyanide for Multicomponent Reactions: synthesis, application, and in silico evaluation

Elena Tretyakova*, Anna Smirnova, Oxana Kazakova

Ufa Institute of Chemistry of the Ufa Federal Research Centre of the Russian Academy of Sciences, 71 prosp. Oktyabrya, Ufa 450054, Russian Federation

* Correspondence: tretyakovaelv@gmail.com

Table S1. The physicochemical properties of diterpene derivatives **1-18** calculated with the Swiss ADME database.

Comp.	Molecular formula	MW[a] (g/mol)	nHA[b]	nAHA[c]	F.Csp3[d]	nRB[e]	nHBA[f]	nHBD[g]	MR[i]	TPSA[k] (A^2)
**1**	$C_{27}H_{40}N_2O_4$	456.62	33	0	0.81	5	5	1	131.28	89.70
**2**	$C_{28}H_{40}N_2O_5$	484.63	35	0	0.79	7	5	1	136.77	92.78
**3**	$C_{28}H_{38}N_2O_4$	466.61	34	0	0.79	5	5	0	132.90	63.68
**4**	$C_{34}H_{44}N_2O_8$	608.72	44	5	0.68	11	8	1	164.26	132.22
**5**	$C_{36}H_{46}N_2O_7$	618.76	45	6	0.64	11	7	1	171.99	119.08
**6**	$C_{35}H_{45}N_3O_7$	619.75	45	6	0.66	11	8	1	169.79	131.97
**7**	$C_{37}H_{48}N_2O_8$	648.79	47	6	0.65	12	8	1	178.48	128.31
**8**	$C_{40}H_{55}N_3O_7$	689.88	50	5	0.72	12	7	1	192.80	126.23
**9**	$C_{42}H_{57}N_3O_6$	699.92	51	6	0.69	12	6	1	200.53	113.09
**10**	$C_{41}H_{56}N_4O_6$	700.91	51	6	0.71	12	7	1	198.33	125.98
**11**	$C_{43}H_{59}N_3O_7$	729.94	53	6	0.70	13	7	1	207.02	122.32
**12**	$C_{41}H_{51}N_3O_7$	697.86	51	11	0.59	13	7	1	195.36	126.23
**13**	$C_{43}H_{53}N_3O_6$	707.90	52	12	0.56	13	6	1	203.10	113.09
**14**	$C_{42}H_{52}N_4O_6$	708.89	52	12	0.57	13	7	1	200.89	125.98
**15**	$C_{44}H_{55}N_3O_7$	737.92	54	12	0.57	14	7	1	209.59	122.32
**16**	$C_{33}H_{48}N_6O_5$	608.77	44	5	0.82	8	9	0	170.71	119.75
**17**	$C_{35}H_{52}N_6O_4$	620.83	45	5	0.83	9	8	1	175.23	119.31
**18**	$C_{36}H_{48}N_6O_4$	628.80	46	11	0.67	10	8	1	177.79	119.31

[a] Molecular weight; [b] number of heavy atom; [c] number of arom. heavy atom; [d] number of sp3 hybridized carbon out of total carbon count; [e] number of rotatable bonds; [f] number of H-bond acceptors; [g] number of H-bond donors, [i] molar refractivity; [k] topological polar surface area.

Table S2. The characteristics of lipophilicity and water solubility of compounds **1-18**.

**Comp.**	**Lipophilicity Consensus Log $P_{o/w}$**	**Water solubility**					
		**Log S (ESOL)**	**Solubility class**	**Log S (Ali)**	**Solubility class**	**Log S (SILICOS-IT)**	**Solubility class**
**1**	3.56	-4.53	Moderately soluble	-5.04	Moderately soluble	-4.54	Moderately soluble
**2**	3.56	-4.74	Moderately soluble	-5.38	Moderately soluble	-4.87	Moderately soluble
**3**	3.09	-4.99	Moderately soluble	-5.14	Moderately soluble	-4.98	Moderately soluble
**4**	4.03	-5.93	Moderately soluble	-7.21	Poorly soluble	-6.54	Poorly soluble
**5**	4.61	-6.39	Poorly soluble	-7.55	Poorly soluble	-7.31	Poorly soluble
**6**	3.94	-5.72	Moderately soluble	-6.71	Poorly soluble	-6.94	Poorly soluble
**7**	4.58	-6.49	Poorly soluble	-7.72	Poorly soluble	-7.40	Poorly soluble
**8**	5.11	-7.25	Poorly soluble	-8.55	Poorly soluble	-7.46	Poorly soluble
**9**	5.68	-7.71	Poorly soluble	-8.90	Poorly soluble	-8.23	Poorly soluble
**10**	4.98	-7.04	Poorly soluble	-8.06	Poorly soluble	-7.86	Poorly soluble
**11**	5.58	-7.81	Poorly soluble	-9.06	Poorly soluble	-8.31	Poorly soluble
**12**	5.04	-7.12	Poorly soluble	-8.22	Poorly soluble	-8.93	Poorly soluble
**13**	5.63	-7.58	Poorly soluble	-8.58	Poorly soluble	-9.70	Poorly soluble
**14**	4.86	-6.91	Poorly soluble	-7.74	Poorly soluble	-9.33	Poorly soluble
**15**	5.52	-7.67	Poorly soluble	-8.74	Poorly soluble	-9.78	Poorly soluble
**16**	3.35	-5.53	Moderately soluble	-5.95	Moderately soluble	-5.09	Moderately soluble
**17**	4.53	-6.61	Poorly soluble	-7.71	Poorly soluble	-6.38	Poorly soluble
**18**	4.47	-6.49	Poorly soluble	-7.39	Poorly soluble	-7.85	Poorly soluble

Table S3. Drug-likeness scores and medicinal chemistry properties of compounds **1-18**

Comp.	Drug-likeness rules					Medicinal chemistry			
	**Lipinski**	**Ghose**	**Veber**	**Egan**	**Muegge**	**PAINS**	**Brenk**	**Lead likeness**	**Synthetic accessibility**
**1**	Yes	No; 2 violations: MR>130, #atoms>70	Yes	Yes	Yes	0 alert	2 alerts: isolated_alkene, phthalimide	No; 1 violation: MW>350	5.78
**2**	Yes	No; 3 violations: MW>480, MR>130, #atoms>70	Yes	Yes	Yes	0 alert	3 alerts: aldehyde, isolated_alkene, phthalimide	No; 2 violations: MW>350, XLOGP3>3.5	5.87
**3**	Yes	No; 2 violations: MR>130, #atoms>70	Y	Y	Y	0 alert	3 alerts: carbo_cation_anion, isolated_alkene, phthalimide	No; 2 violations: MW>350, XLOGP3>3.5	5.98
**4**	Yes; 1 violation: MW>500	No; 3 violations: MW>480, MR>130, #atoms>70	No; 1 violation: Rotors>10	No; 1 violation: TPSA>131.6	No; 1 violation: MW>600	0 alert	3 alerts: isolated_alkene, more_than_2_esters, phthalimide	No; 3 violations: MW>350, Rotors>7, XLOGP3>3.5	6.68
**5**	No; 2 violations: MW>500, MLOGP>4.15	No; 3 violations: MW>480, MR>130, #atoms>70	No; 1 violation: Rotors>10	Yes	No; 2 violations: MW>600, XLOGP3>5	0 alert	3 alerts: isolated_alkene, more_than_2_esters, phthalimide	No; 3 violations: MW>350, Rotors>7, XLOGP3>3.5	6.48
**6**	Yes; 1 violation: MW>500	No; 3 violations: MW>480, MR>130, #atoms>70	No; 1 violation: Rotors>10	No; 1 violation: TPSA>131.6	No; 1 violation: MW>600	0 alert	3 alerts: isolated_alkene, more_than_2_esters, phthalimide	No; 3 violations: MW>350, Rotors>7, XLOGP3>3.5	6.40
**7**	Yes; 1 violation: MW>500	No; 3 violations: MW>480, MR>130, #atoms>70	No; 1 violation: Rotors>10	Yes	No; 2 violations: MW>600, XLOGP3>5	0 alert	3 alerts: isolated_alkene, more_than_2_esters, phthalimide	No; 3 violations: MW>350, Rotors>7, XLOGP3>3.5	6.62

**8**	Yes; 1 violation: MW>500	No; 3 violations: MW>480, MR>130, #atoms>70	No; 1 violation: Rotors >10	Yes	No; 2 violations: MW>600, XLOGP3>5	0 alert	2 alerts: isolated_alkene, phthalimide	No; 3 violations: MW>350, Rotors>7, XLOGP3>3.5	7.26
**9**	No; 2 violations: MW>500, MLOGP>4.15	No; 4 violations: MW>480, WLOGP>5.6, MR>130, #atoms>70	No; 1 violation: Rotors >10	Yes	No; 2 violations: MW>600, XLOGP3>5	0 alert	2 alerts: isolated_alkene, phthalimide	No; 3 violations: MW>350, Rotors>7, XLOGP3>3.5	7.04
**10**	Yes; 1 violation: MW>500	No; 3 violations: MW>480, MR>130, #atoms>70	No; 1 violation: Rotors >10	Yes	No; 2 violations: MW>600, XLOGP3>5	0 alert	2 alerts: isolated_alkene, phthalimide	No; 3 violations: MW>350, Rotors>7, XLOGP3>3.5	7.02
**11**	No; 2 violations: MW>500, MLOGP>4.15	No; 4 violations: MW>480, WLOGP>5.6, MR>130, #atoms>70	No; 1 violation: Rotors >10	Yes	No; 2 violations: MW>600, XLOGP3>5	0 alert	2 alerts: isolated_alkene, phthalimide	No; 3 violations: MW>350, Rotors>7, XLOGP3>3.5	7.20
**12**	Yes; 1 violation: MW>500	No; 3 violations: MW>480, MR>130, #atoms>70	No; 1 violation: Rotors >10	Yes	No; 2 violations: MW>600, XLOGP3>5	0 alert	2 alerts: isolated_alkene, phthalimide	No; 3 violations: MW>350, Rotors>7, XLOGP3>3.5	7.08
**13**	No; 2 violations: MW>500, MLOGP>4.15	No; 3 violations: MW>480, MR>130, #atoms>70	No; 1 violation: Rotors >10	Yes	No; 2 violations: MW>600, XLOGP3>5	0 alert	2 alerts: isolated_alkene, phthalimide	No; 3 violations: MW>350, Rotors>7, XLOGP3>3.5	6.91
**14**	Yes; 1 violation: MW>500	No; 3 violations: MW>480, MR>130, #atoms>70	No; 1 violation: Rotors >10	Yes	No; 2 violations: MW>600, XLOGP3>5	0 alert	2 alerts: isolated_alkene, phthalimide	No; 3 violations: MW>350, Rotors>7, XLOGP3>3.5	6.87

**15**	No; 2 violations: MW>500, MLOGP>4.15	No; 3 violations: MW>480, MR>130, #atoms>70	No; 1 violation: Rotors>10	Yes	No; 2 violations: MW>600, XLOGP3>5	0 alert	2 alerts: isolated_alkene, phthalimide	No; 3 violations: MW>350, Rotors>7, XLOGP3>3.5	7.07
**16**	No; 2 violations: MW>500, NorO>10	No; 3 violations: MW>480, MR>130, #atoms>70	Yes	Yes	No; 1 violation: MW>600	0 alert	2 alerts: isolated_alkene, phthalimide	No; 3 violations: MW>350, Rotors>7, XLOGP3>3.5	7.02
**17**	Yes; 1 violation: MW>500	No; 3 violations: MW>480, MR>130, #atoms>70	Yes	Yes	No; 2 violations: MW>600, XLOGP3>5	0 alert	2 alerts: isolated_alkene, phthalimide	No; 3 violations: MW>350, Rotors>7, XLOGP3>3.5	7.22
**18**	Yes; 1 violation: MW>500	No; 3 violations: MW>480, MR>130, #atoms>70	Yes	Yes	No; 2 violations: MW>600, XLOGP3>5	0 alert	2 alerts: isolated_alkene, phthalimide	No; 3 violations: MW>350, Rotors>7, XLOGP3>3.5	6.90

Figure S1. ^{1}H NMR spectrum of compound **2** in $CDCl_3$ (500 MHz).

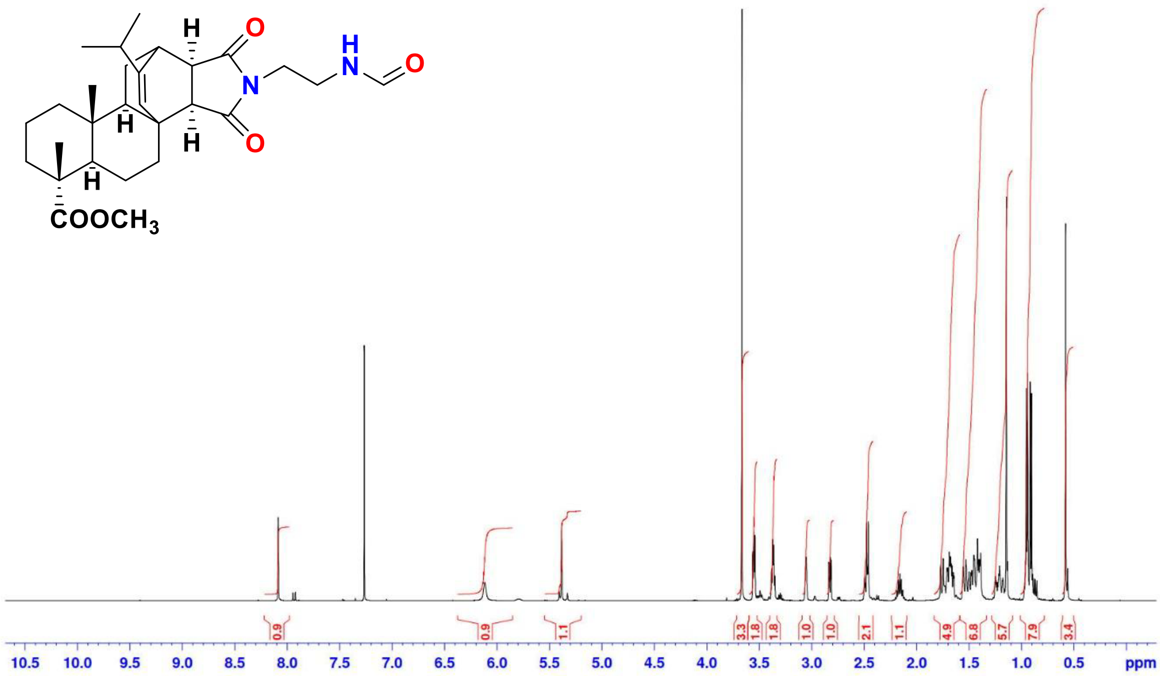

Figure S2. ^{13}C NMR spectrum of compound **2** in $CDCl_3$ (125MHz)

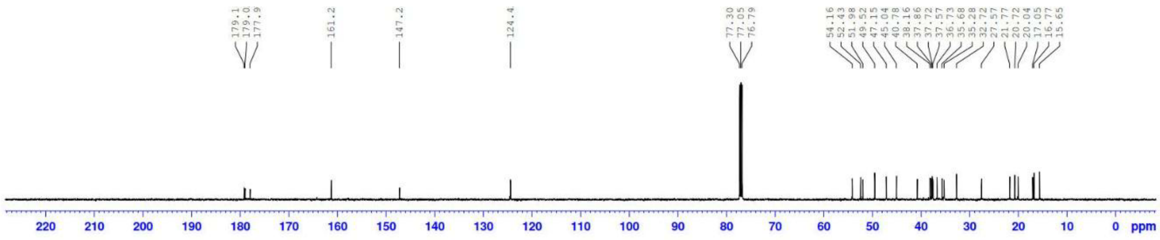

Figure S3. ^{1}H-^{1}H COSY spectrum of compound **2** in $CDCl_3$

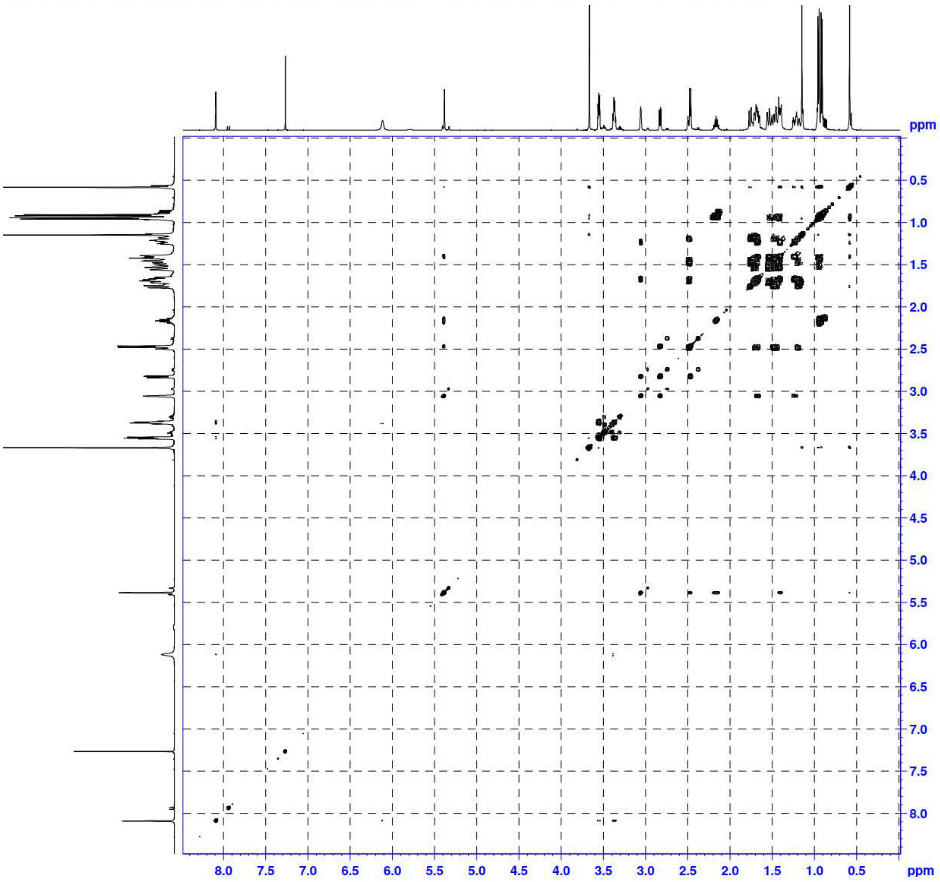

Figure S4. ^{1}H-^{13}C HSQC spectrum of compound **2** in $CDCl_3$

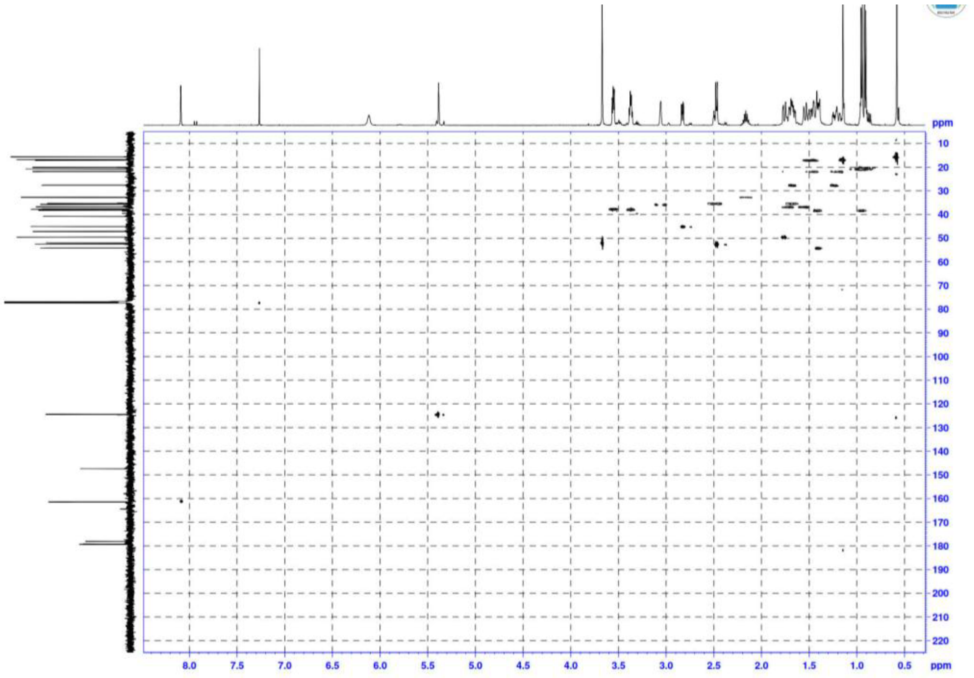

Figure S5. ^{1}H-^{13}C HMBC spectrum of compound **2** in $CDCl_3$

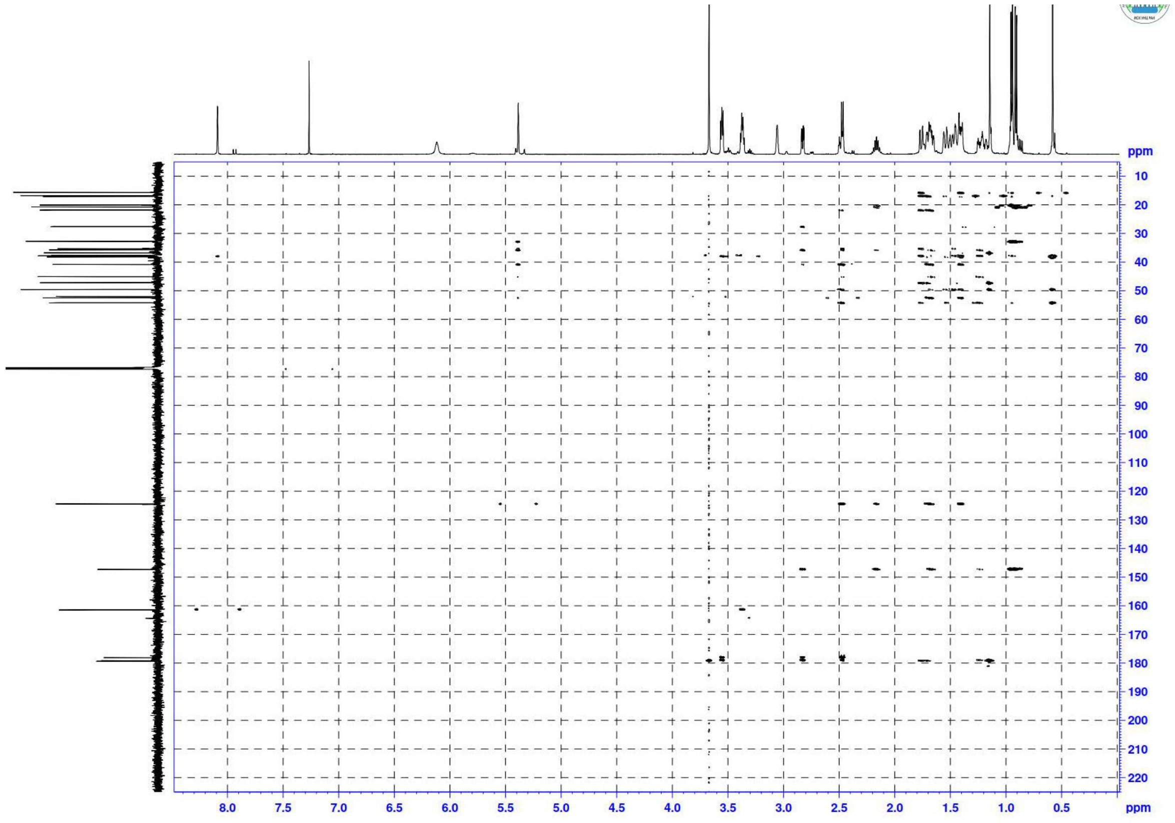

Figure S6. ^{1}H-^{15}N HSQC spectrum of compound **2** in $CDCl_3$

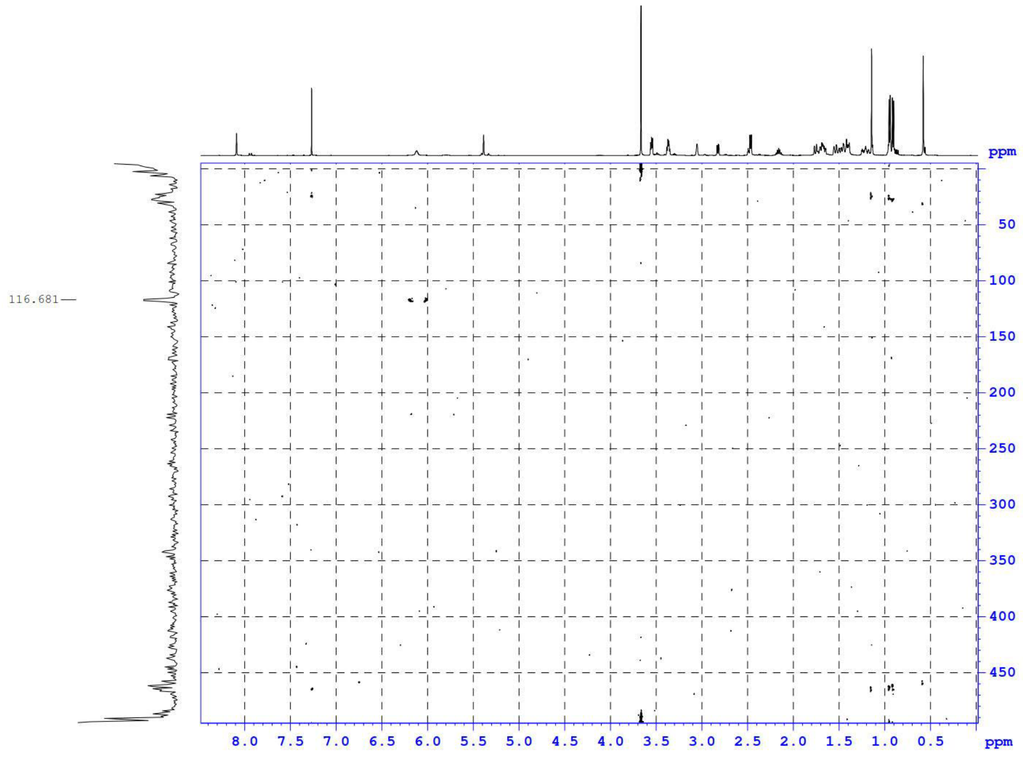

Figure S7. ^{1}H-^{15}N HMBC spectrum of compound **2** in $CDCl_3$

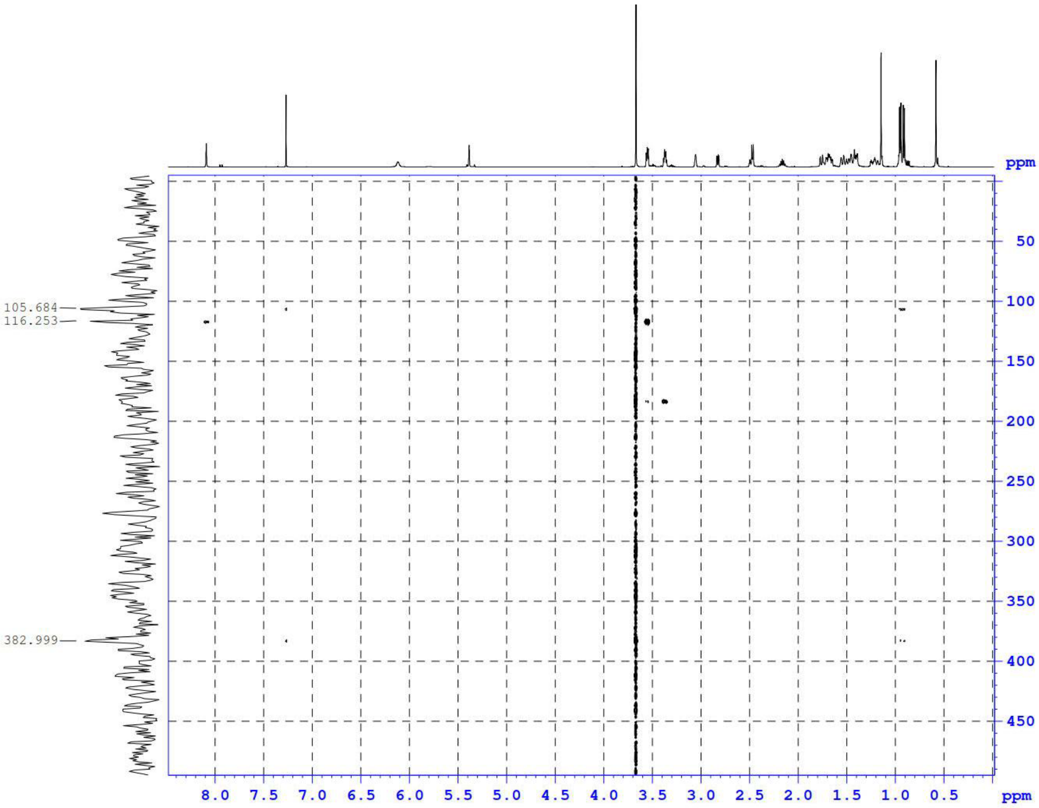

Figure S8. ^{1}H-^{1}H NOESY spectrum of compound **2** in $CDCl_3$

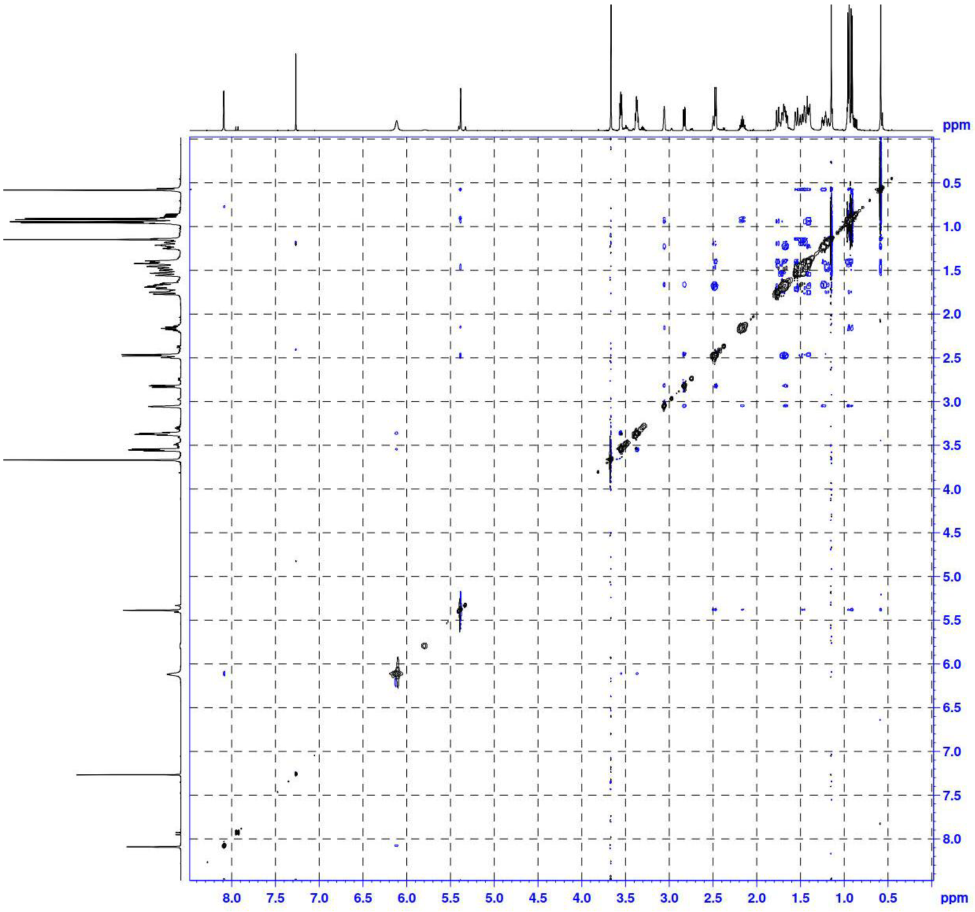

Figure S9. ^{1}H NMR spectrum of compound **3** in $CDCl_3$ (500 MHz)

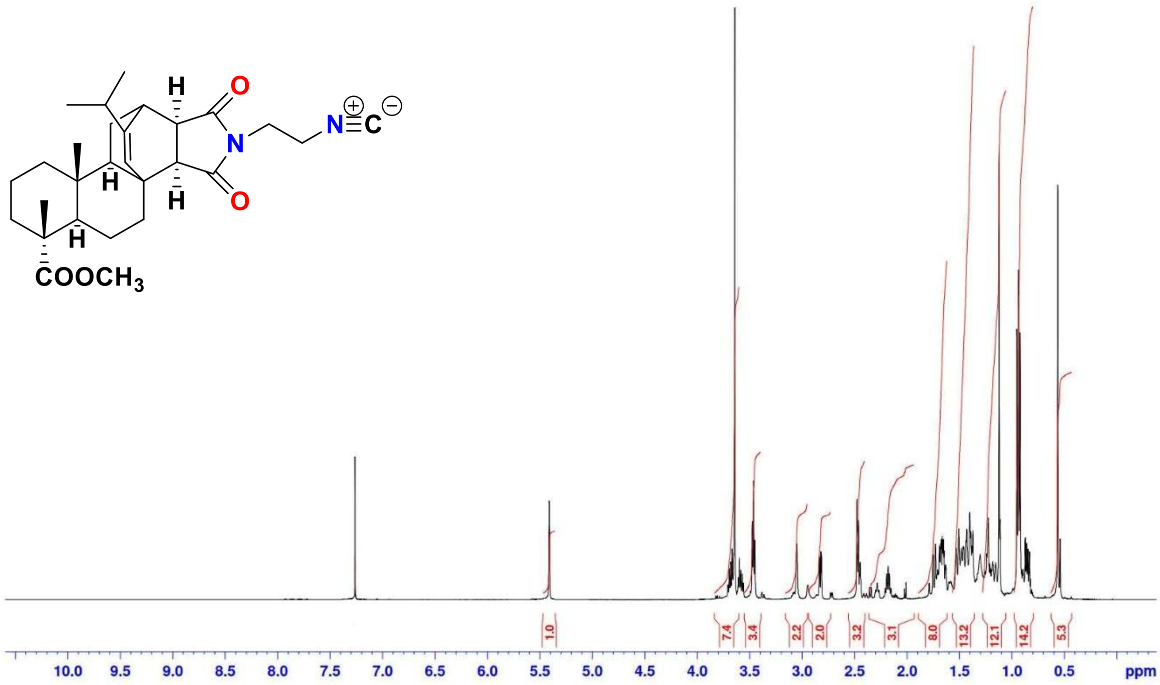

Figure S10. ^{13}C NMR spectrum of compound **3** in $CDCl_3$ (125MHz)

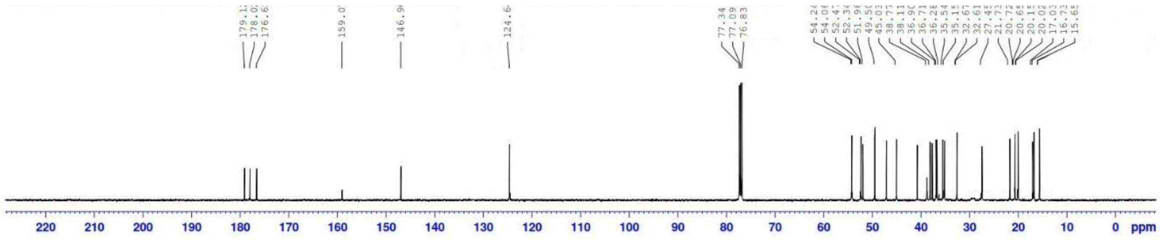

Figure S11. ^{1}H-^{1}H COSY spectrum of compound **3** in $CDCl_3$

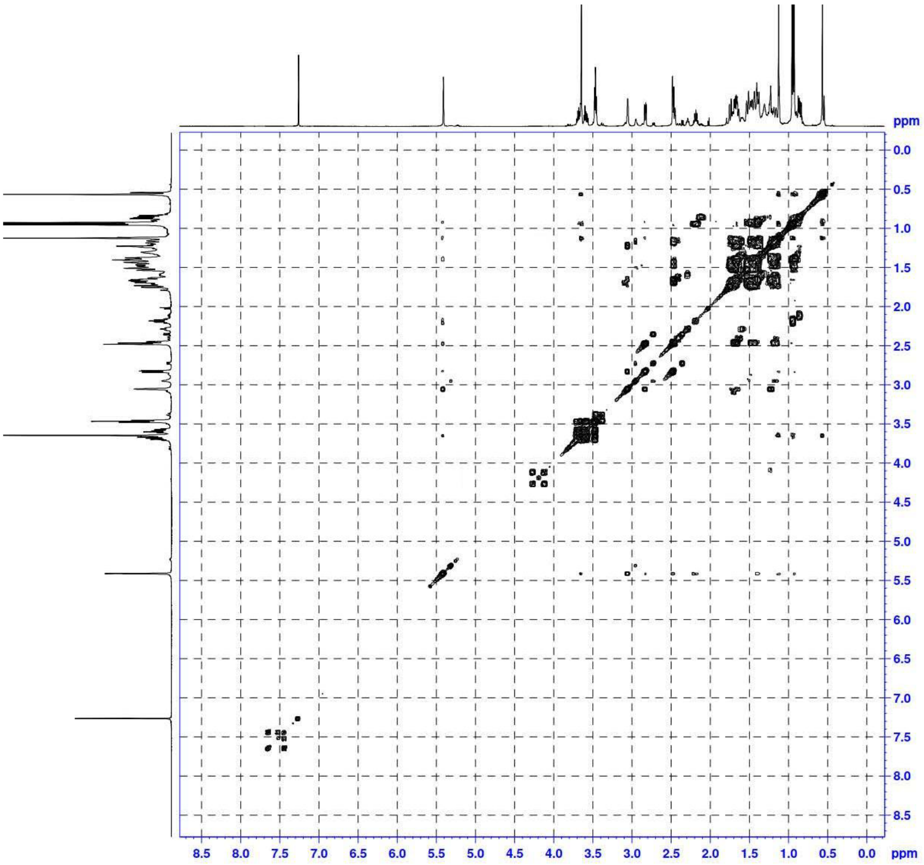

Figure S12. ^{1}H-^{13}C HSQC spectrum of compound **3** in $CDCl_3$

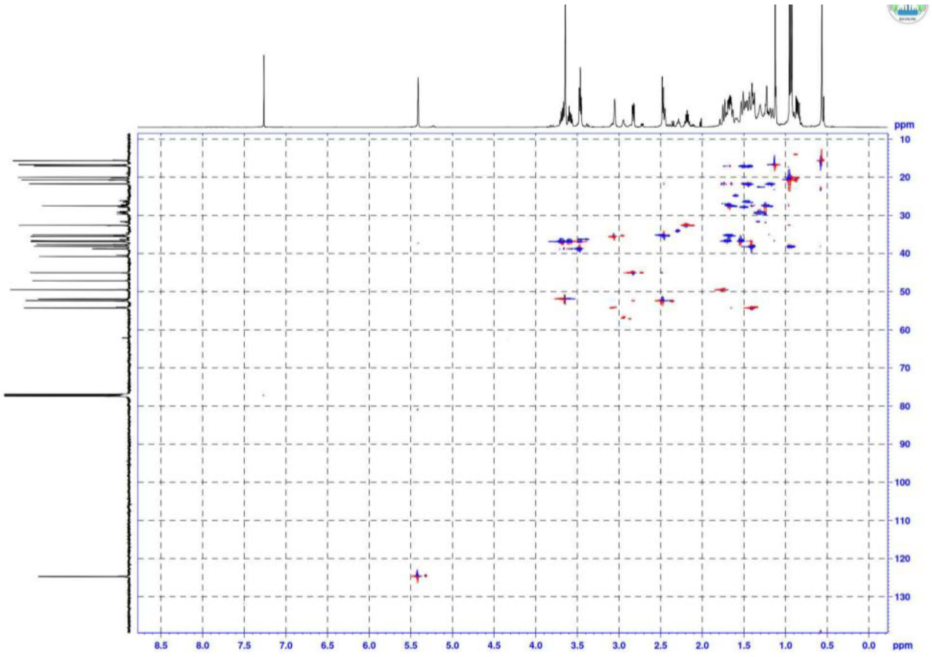

Figure S13. ^{1}H-^{13}C HMBC spectrum of compound **3** in $CDCl_3$

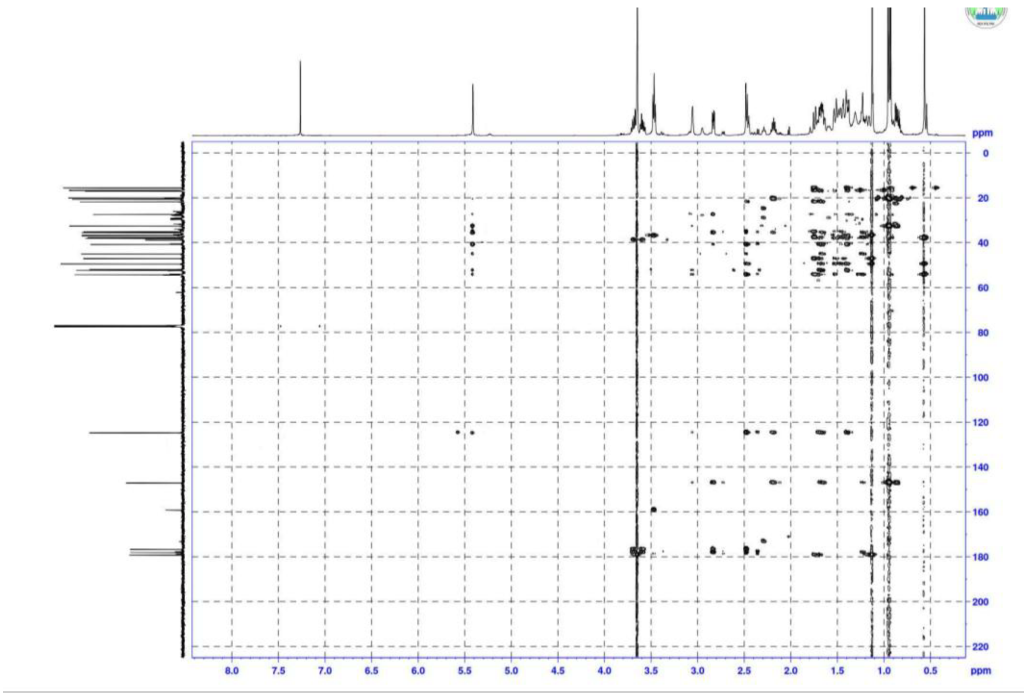

Figure S14. ^{1}H-^{15}N HMBC spectrum of compound **3** in $CDCl_3$

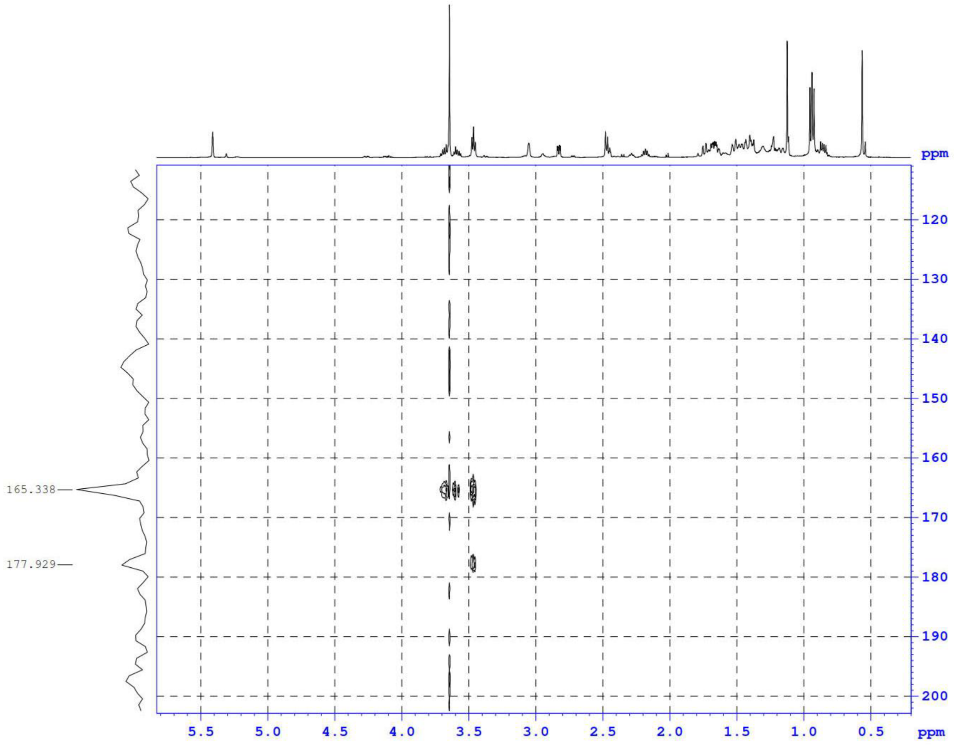

Figure S15. ^{1}H NMR spectrum of compound **4** in $CDCl_3$ (500 MHz)

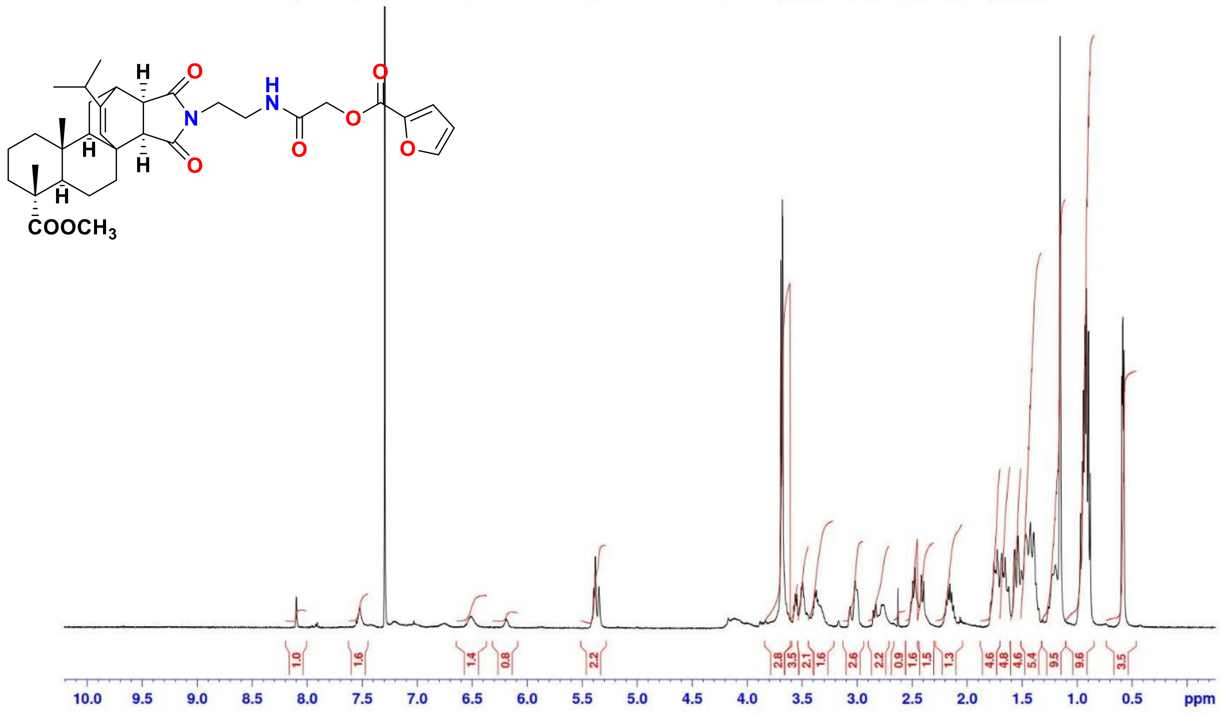

Figure S16. ^{13}C NMR spectrum of compound **4** in $CDCl_3$ (125MHz)

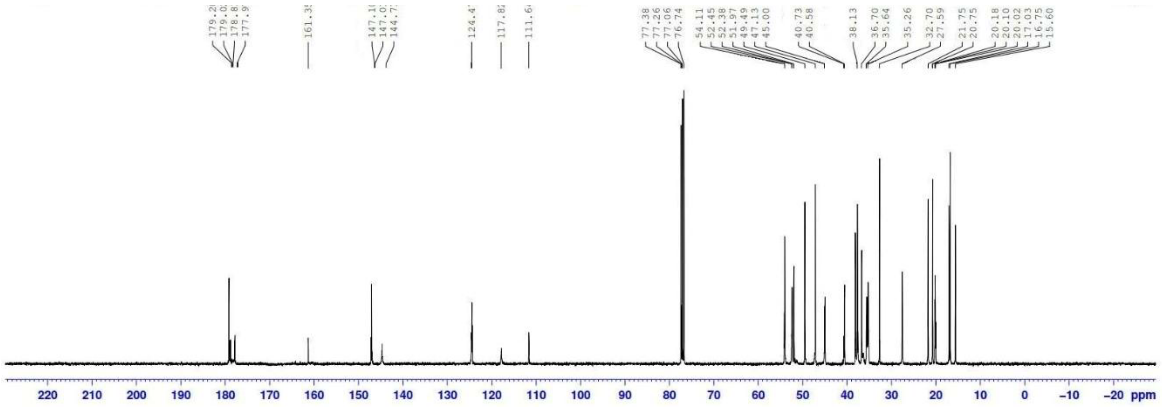

Figure S17. ^{1}H-^{1}H COSY spectrum of compound **4** in $CDCl_3$

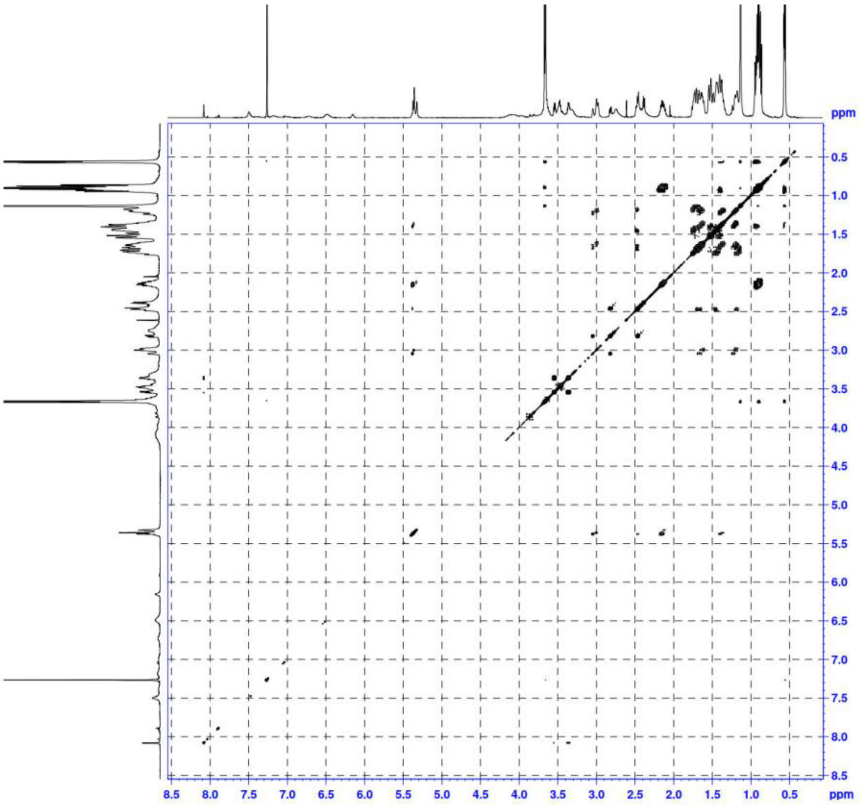

Figure S18. ^{1}H-^{13}C HSQC spectrum of compound **4** in $CDCl_3$

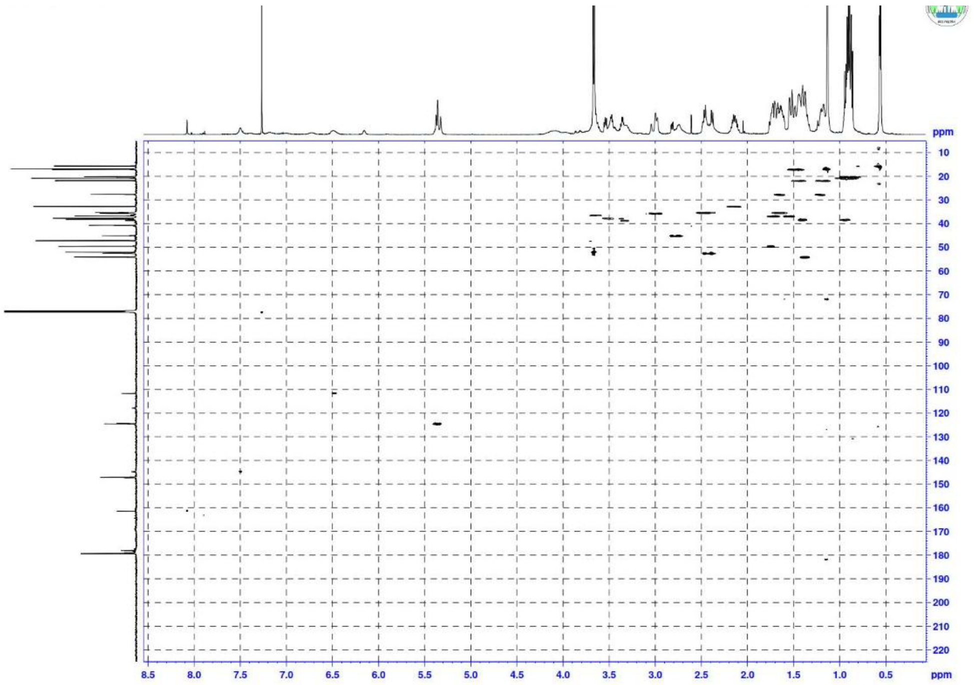

Figure S19. ^{1}H-^{13}C HMBC spectrum of compound **4** in $CDCl_3$

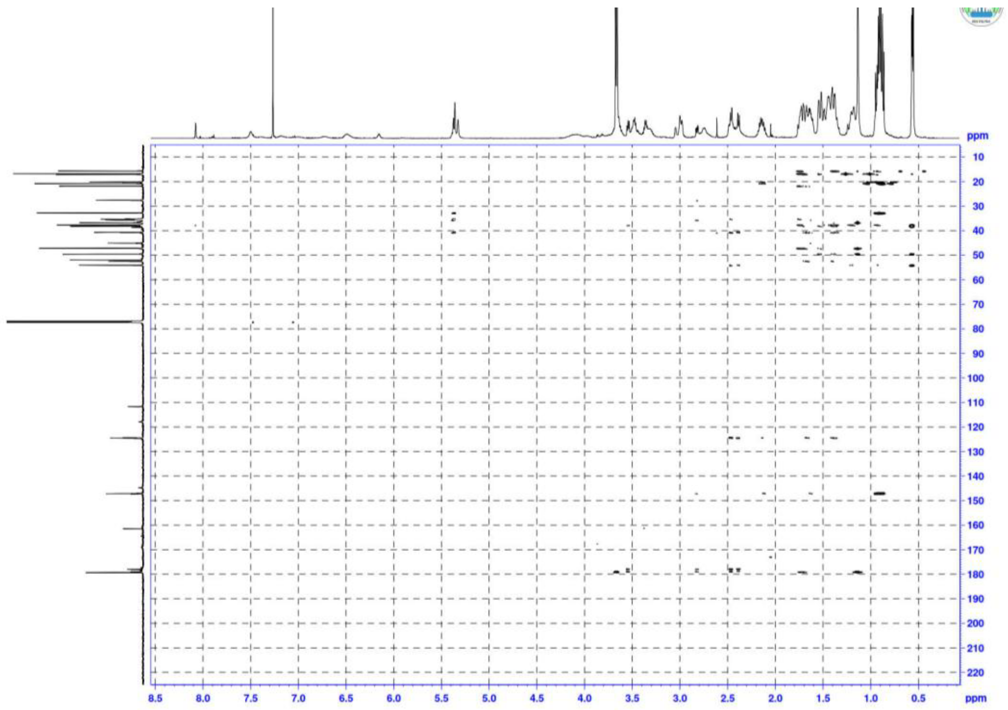

Figure S20. ^{1}H-^{1}H ROESY spectrum of compound **4** in $CDCl_3$

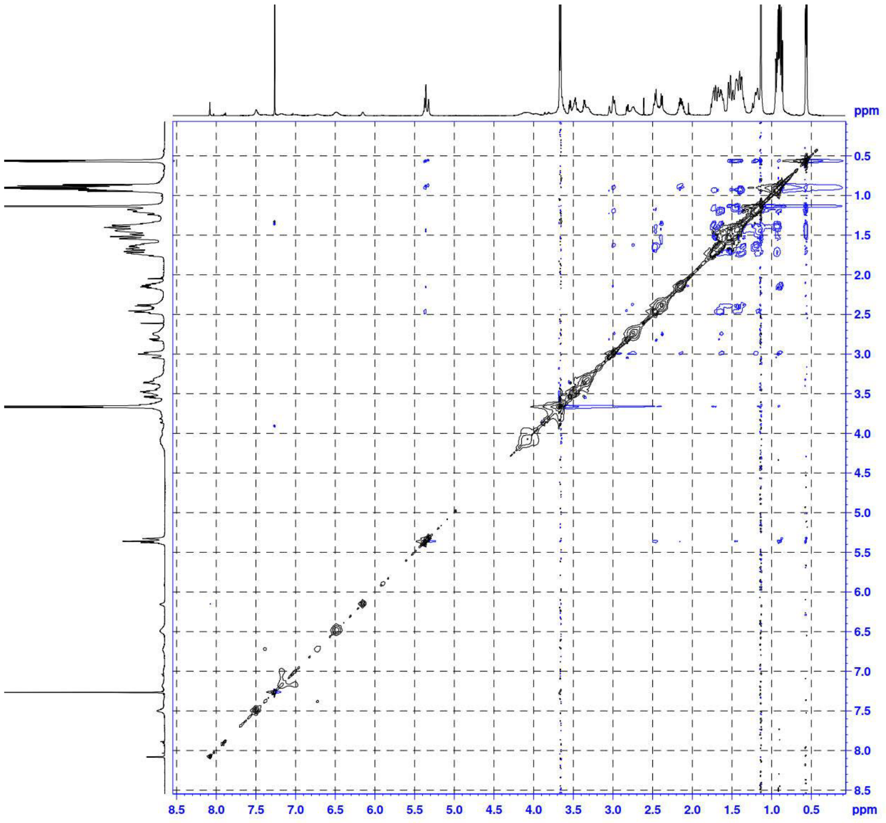

Figure S21. ^{1}H NMR spectrum of compound **5** in $CDCl_3$ (500 MHz)

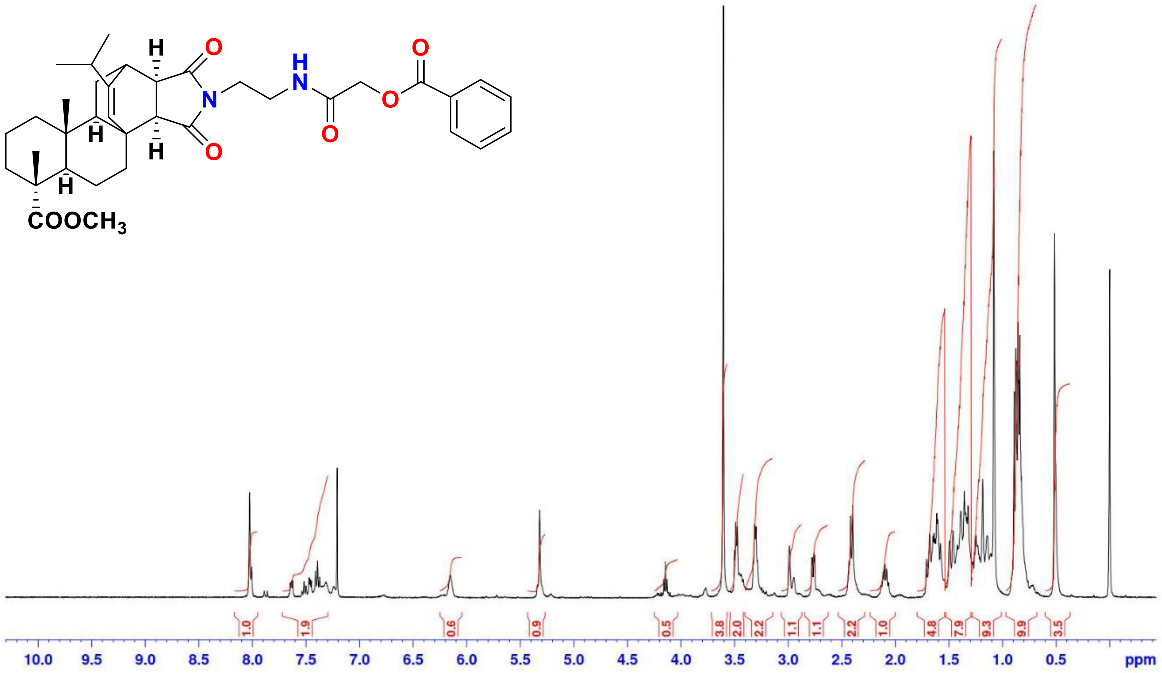

Figure S22. ^{13}C NMR spectrum of compound **5** in $CDCl_3$ (125MHz)

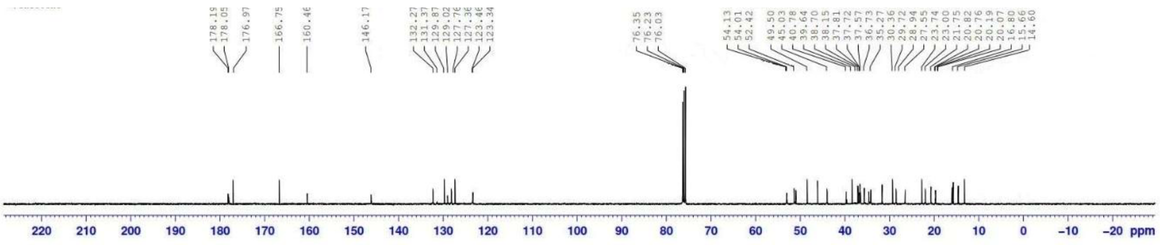

Figure S23. ^{1}H NMR spectrum of compound **6** in $CDCl_3$ (500 MHz)

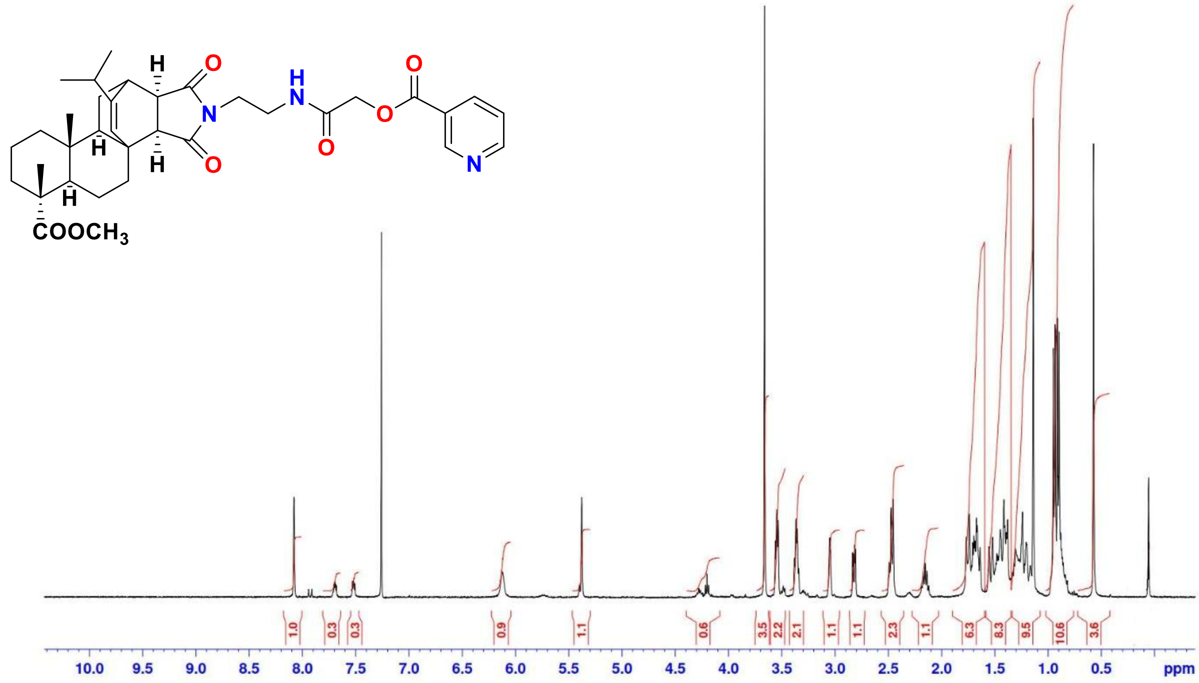

Figure S24. ^{13}C NMR spectrum of compound **6** in $CDCl_3$ (125MHz)

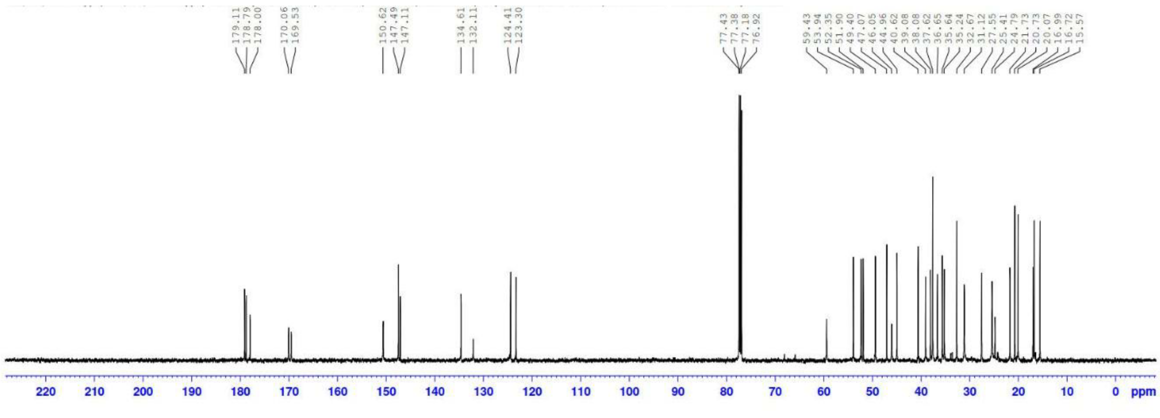

Figure S25. ^{1}H NMR spectrum of compound **7** in $CDCl_3$ (500 MHz)

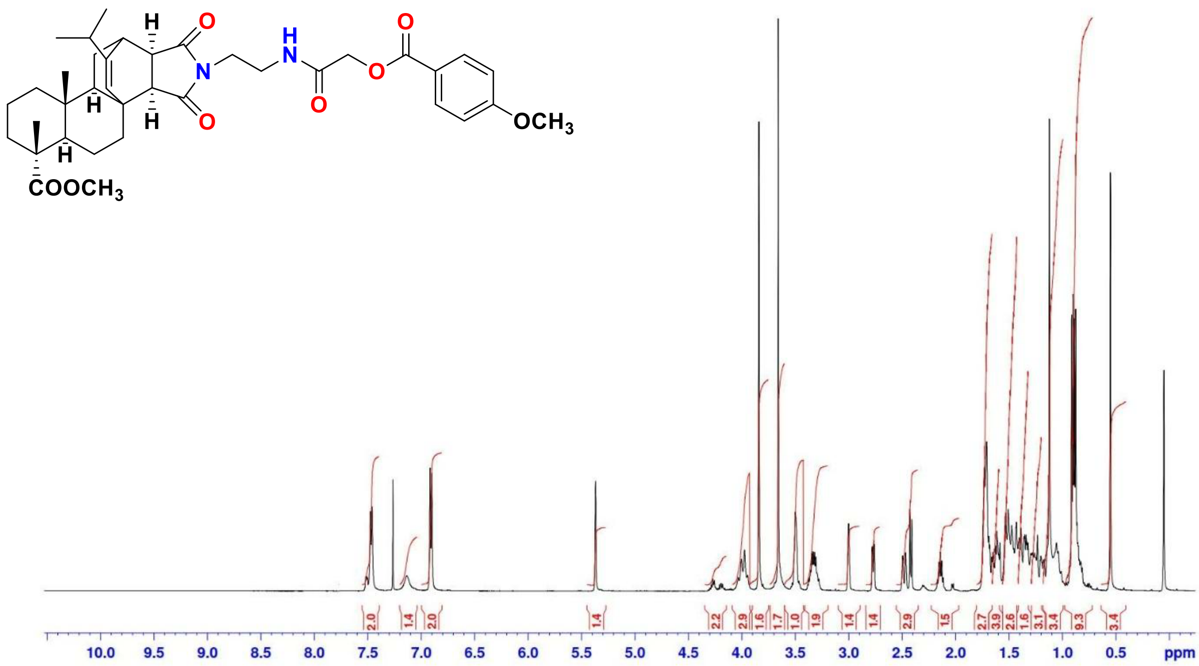

Figure S26. ^{13}C NMR spectrum of compound **7** in $CDCl_3$ (125 MHz)

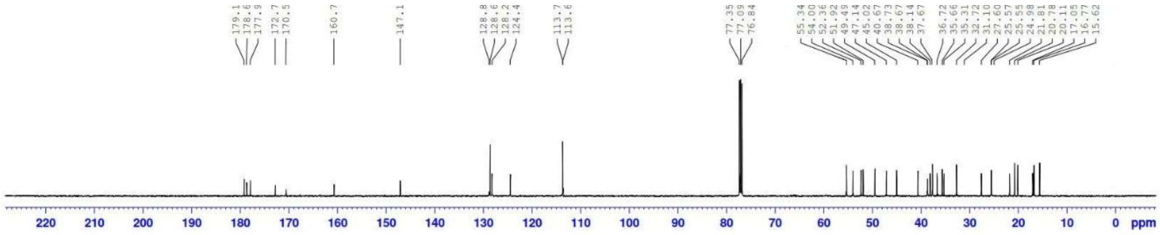

Figure S27. ^{1}H NMR spectrum of compound **8** in $CDCl_3$ (500 MHz)

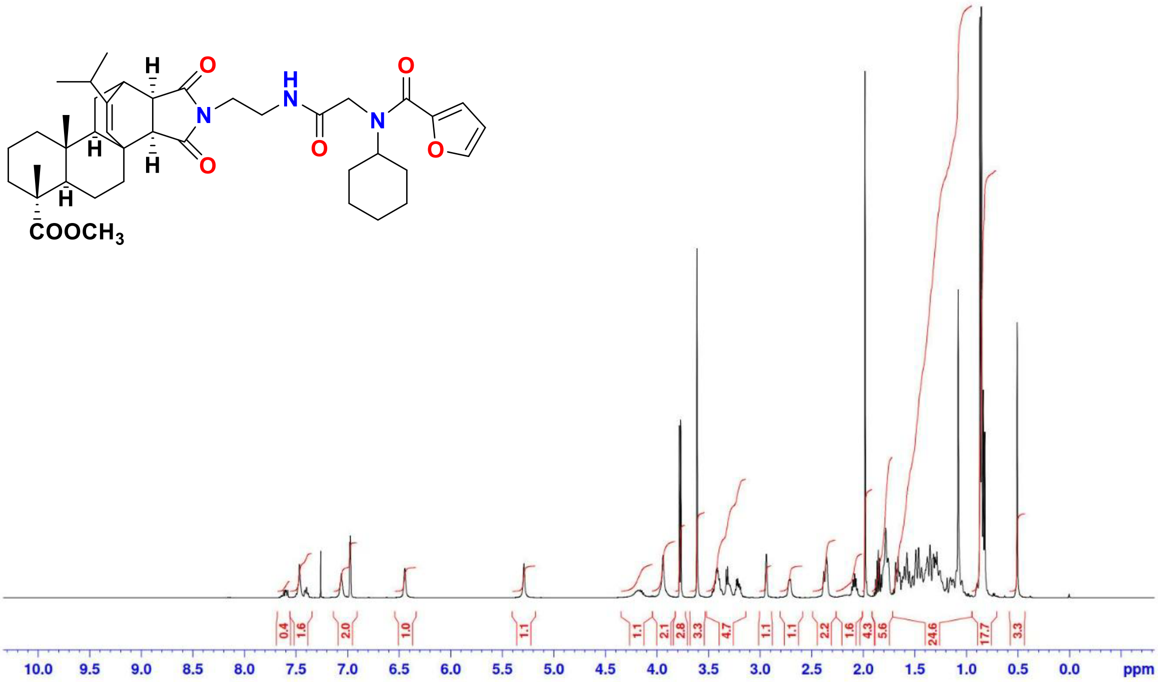

Figure S28. ^{13}C NMR spectrum of compound **8** in $CDCl_3$ (125MHz)

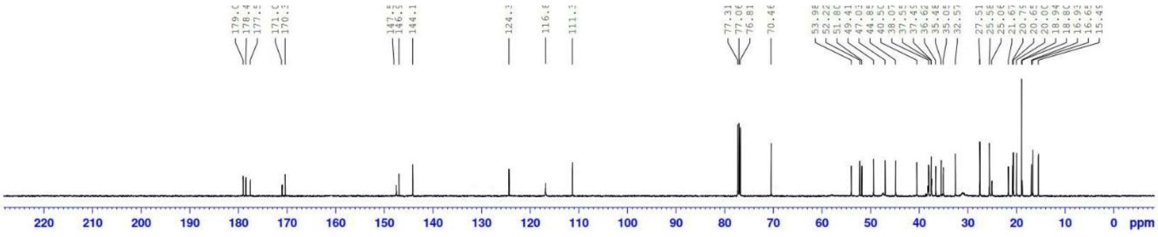

Figure S29. ^{1}H-^{1}H COSY spectrum of compound **8** in $CDCl_3$

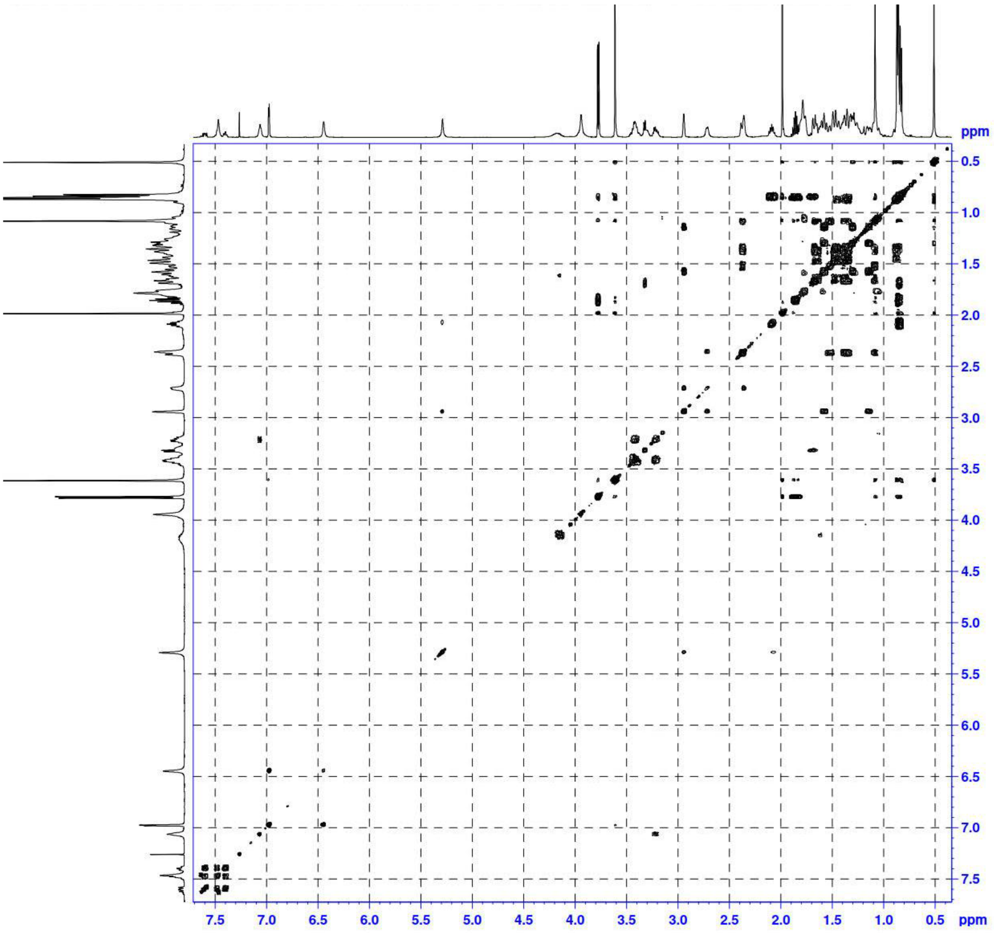

Figure S30. ^{1}H-^{13}C HSQC spectrum of compound **8** in $CDCl_3$

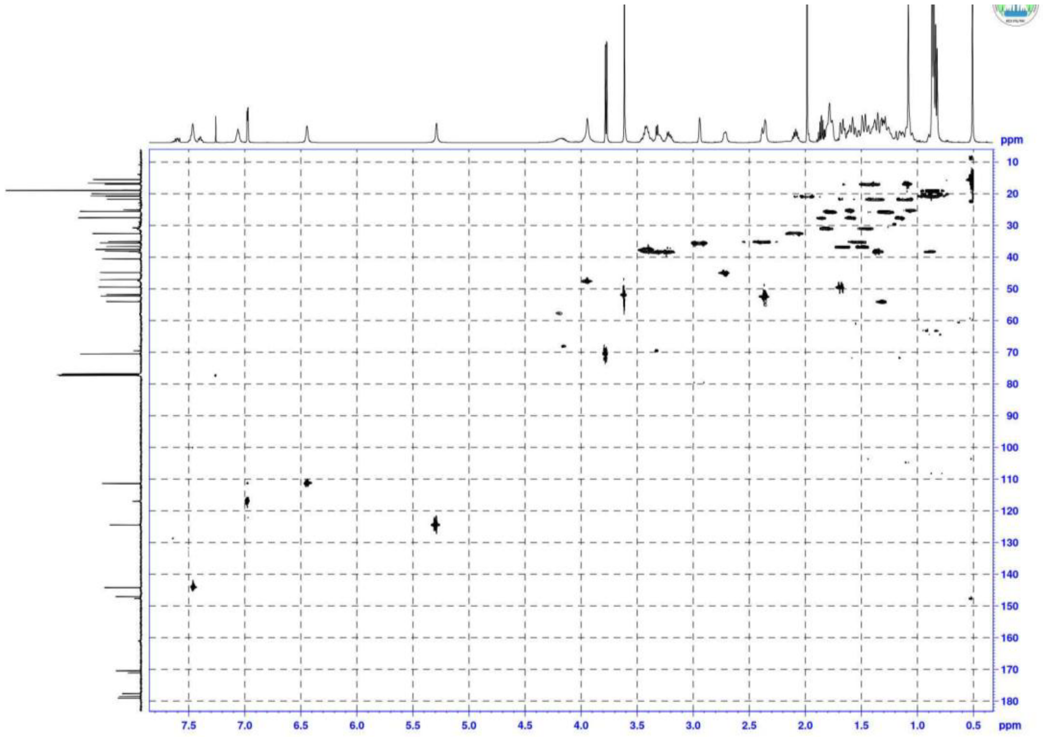

Figure S31. ^{1}H-^{13}C HMBC spectrum of compound **8** in $CDCl_3$

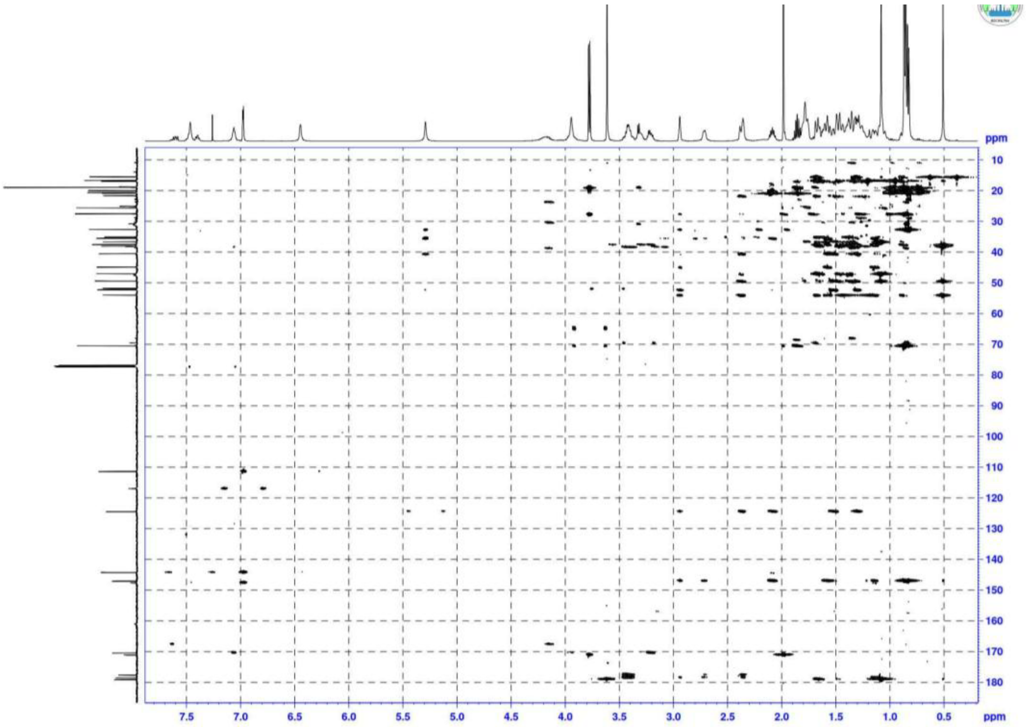

Figure S32. ^{1}H-^{1}H NOESY spectrum of compound **8** in $CDCl_3$

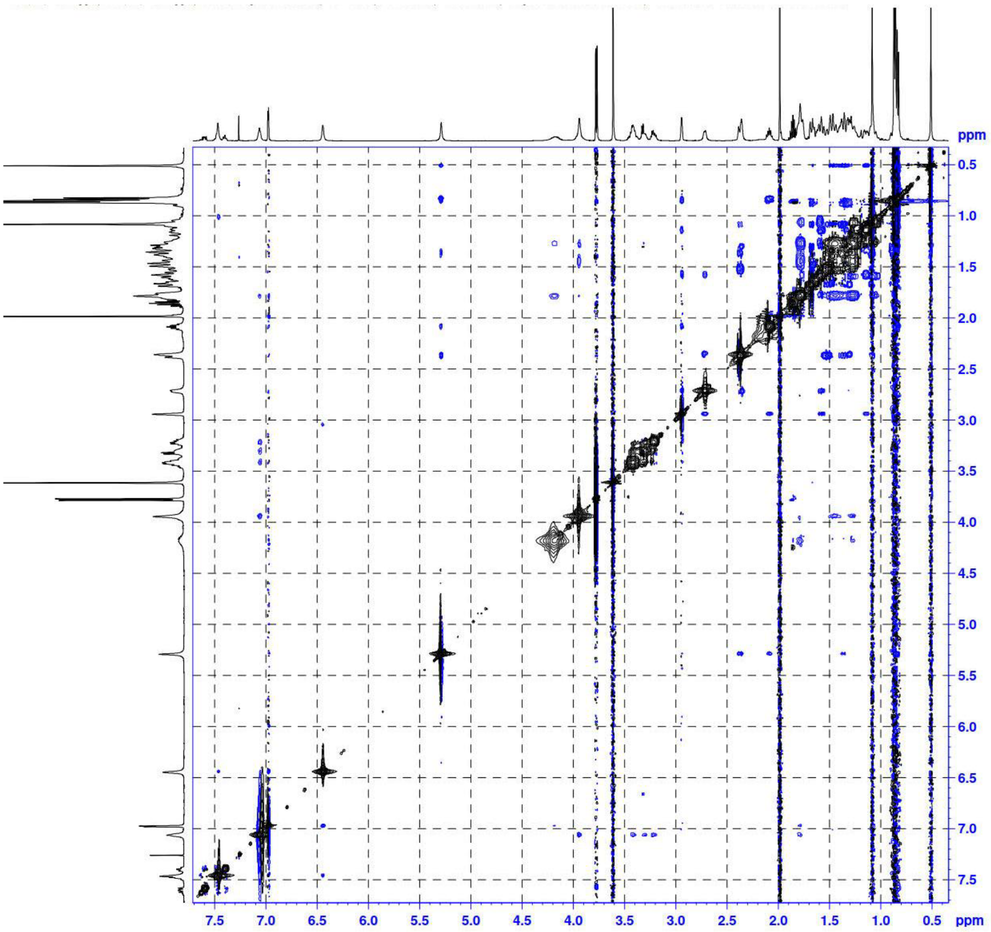

Figure S33. ^{1}H-^{15}N HSQC spectrum of compound **8** in $CDCl_3$

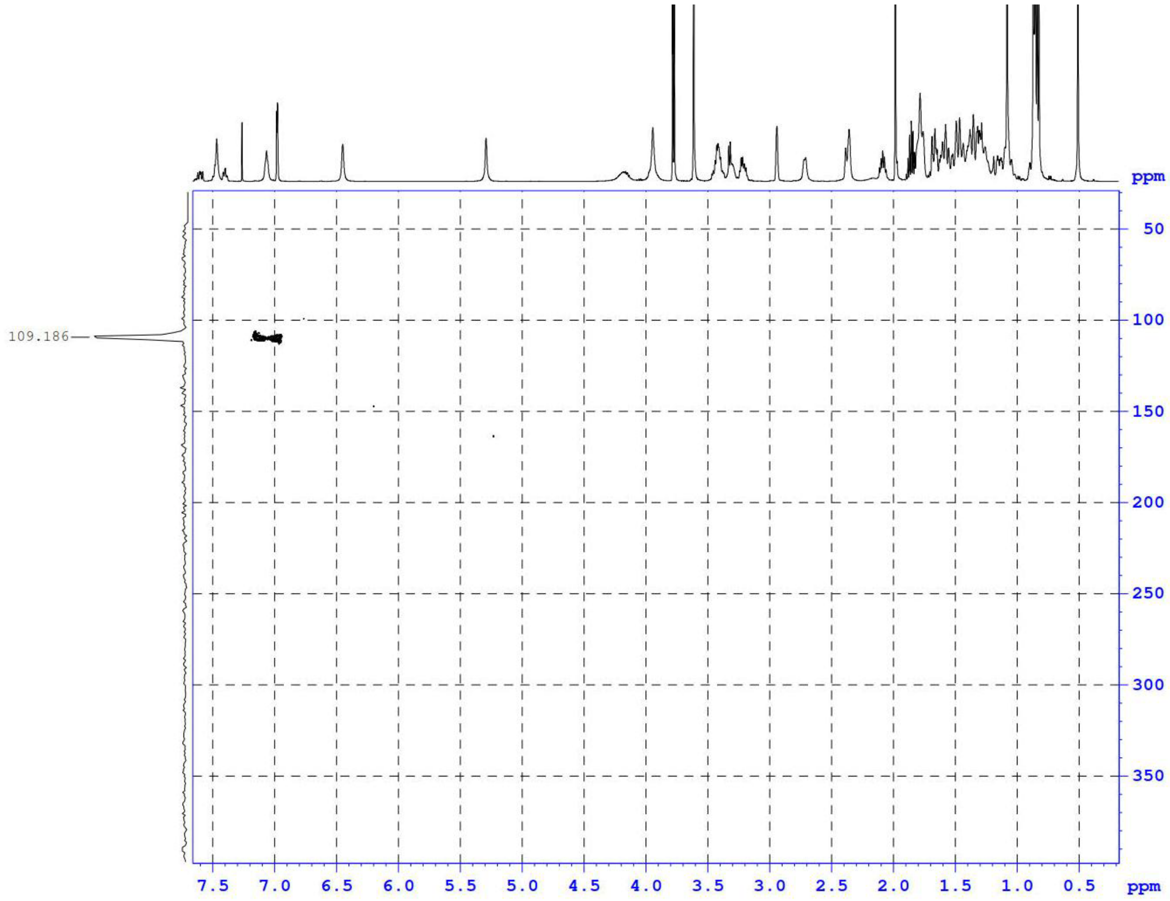

Figure S34. ^{1}H-^{15}N HMBC spectrum of compound **8** in $CDCl_3$

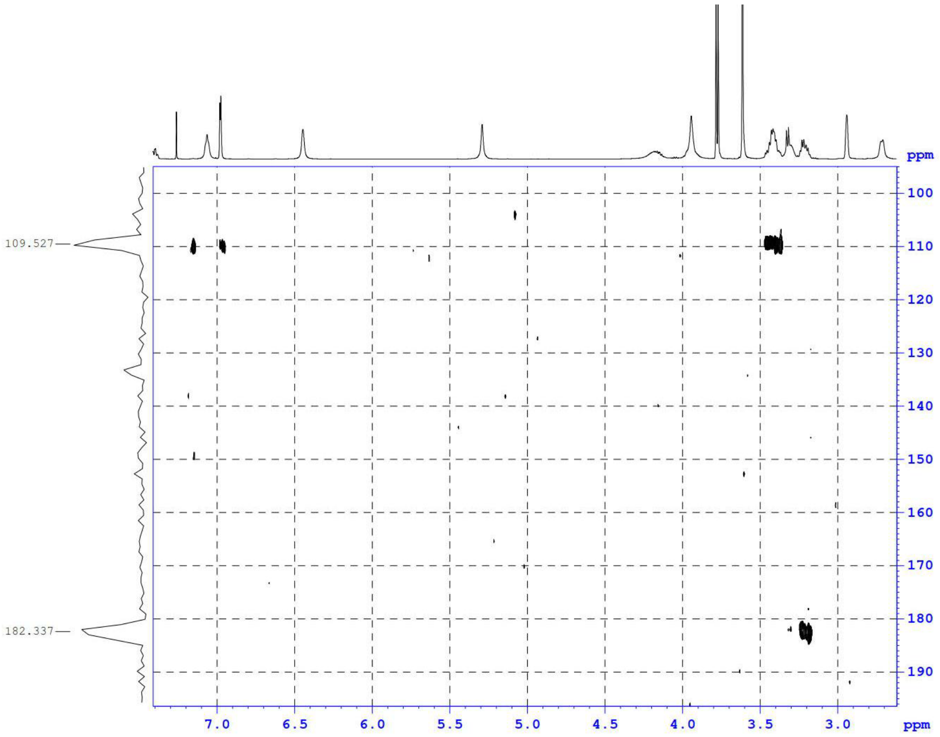

Figure S35. ^{1}H NMR spectrum of compound **9** in $CDCl_3$ (500 MHz)

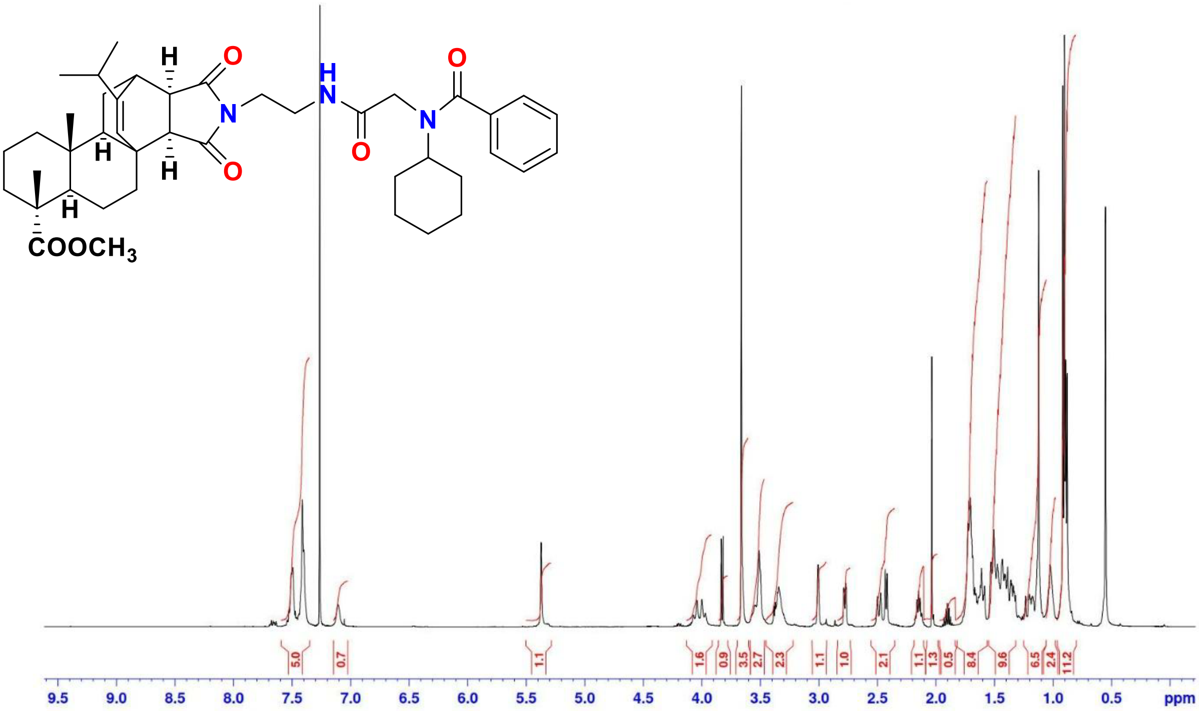

Figure S36. ^{13}C NMR spectrum of compound **9** in $CDCl_3$ (125 MHz)

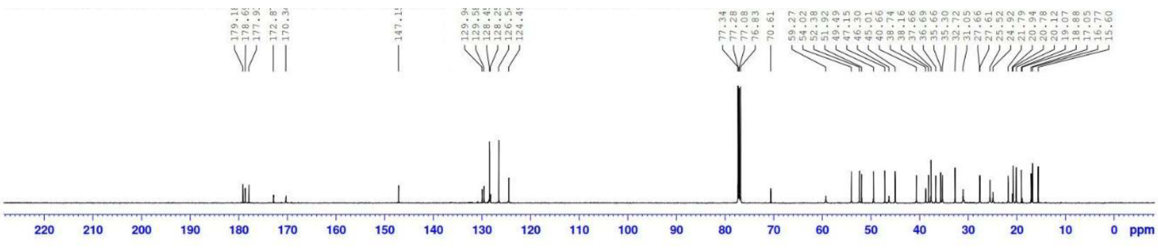

Figure S37. ^{1}H-^{1}H COSY spectrum of compound **9** in $CDCl_3$

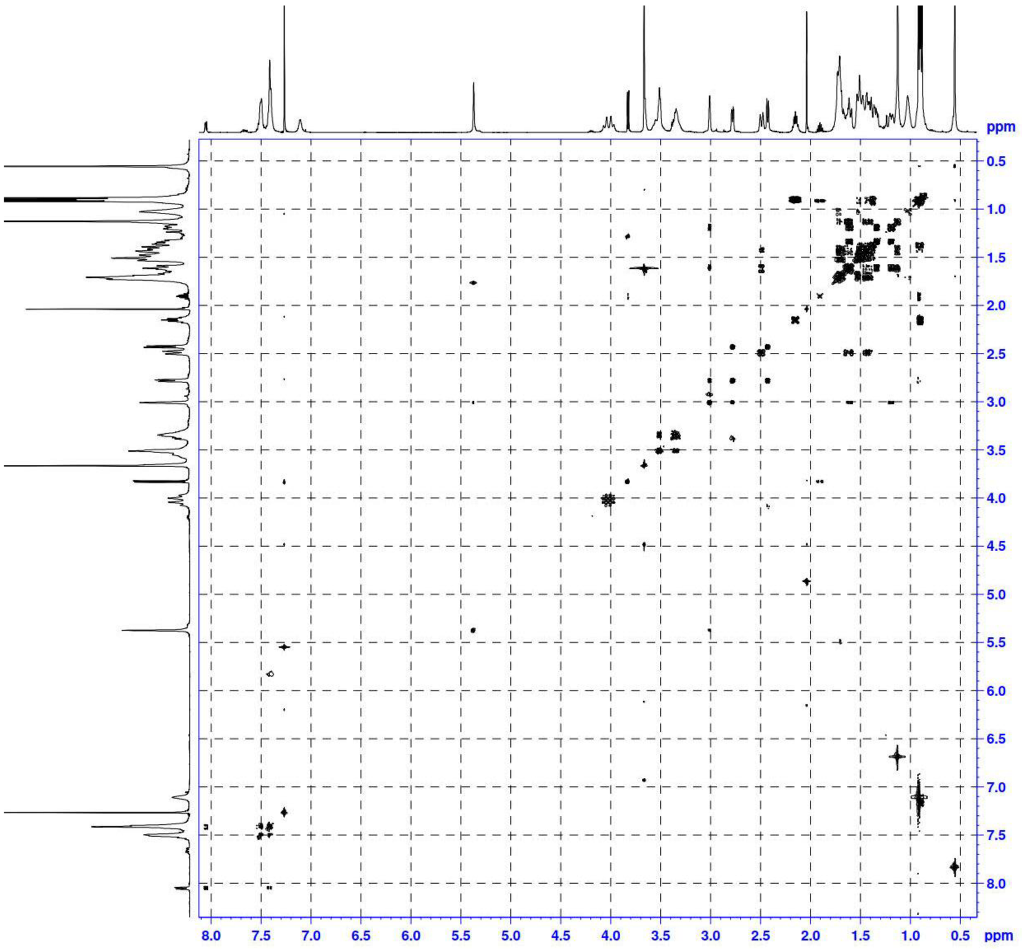

Figure S38. ^{1}H-^{13}C HSQC spectrum of compound **9** in $CDCl_3$

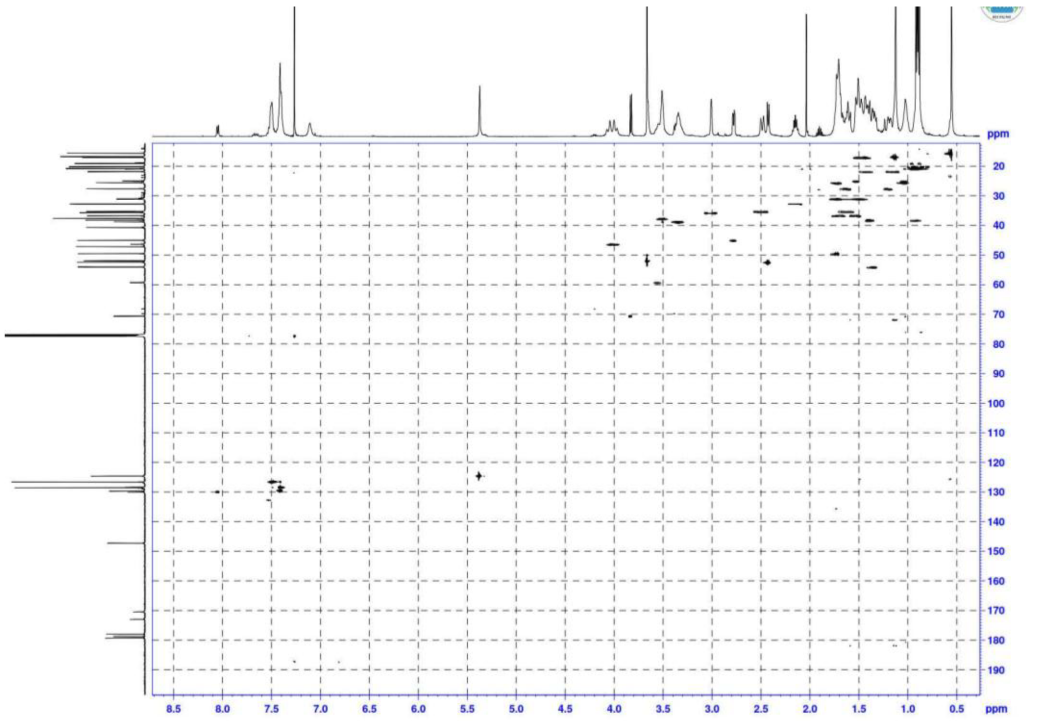

Figure S39. ^{1}H-^{13}C HMBC spectrum of compound **9** in $CDCl_3$

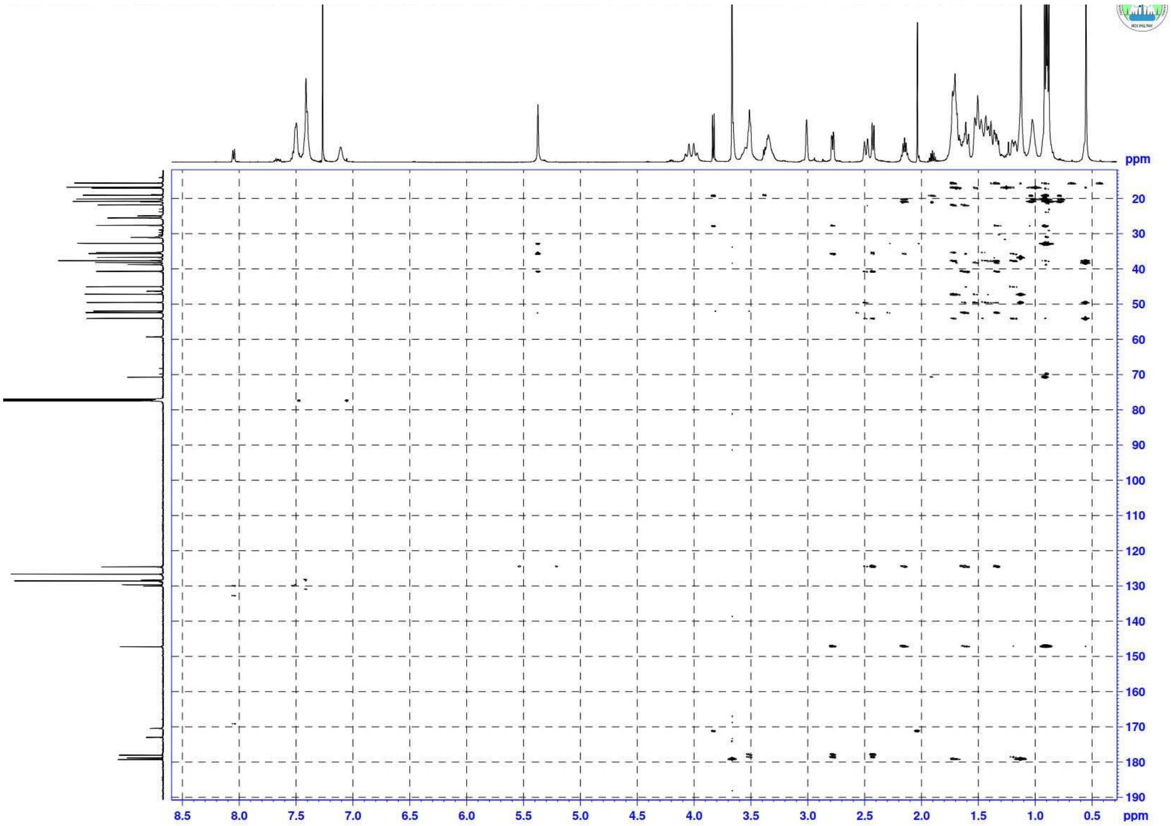

Figure S40. ^{1}H-^{15}N HSQC spectrum of compound **9** in $CDCl_3$

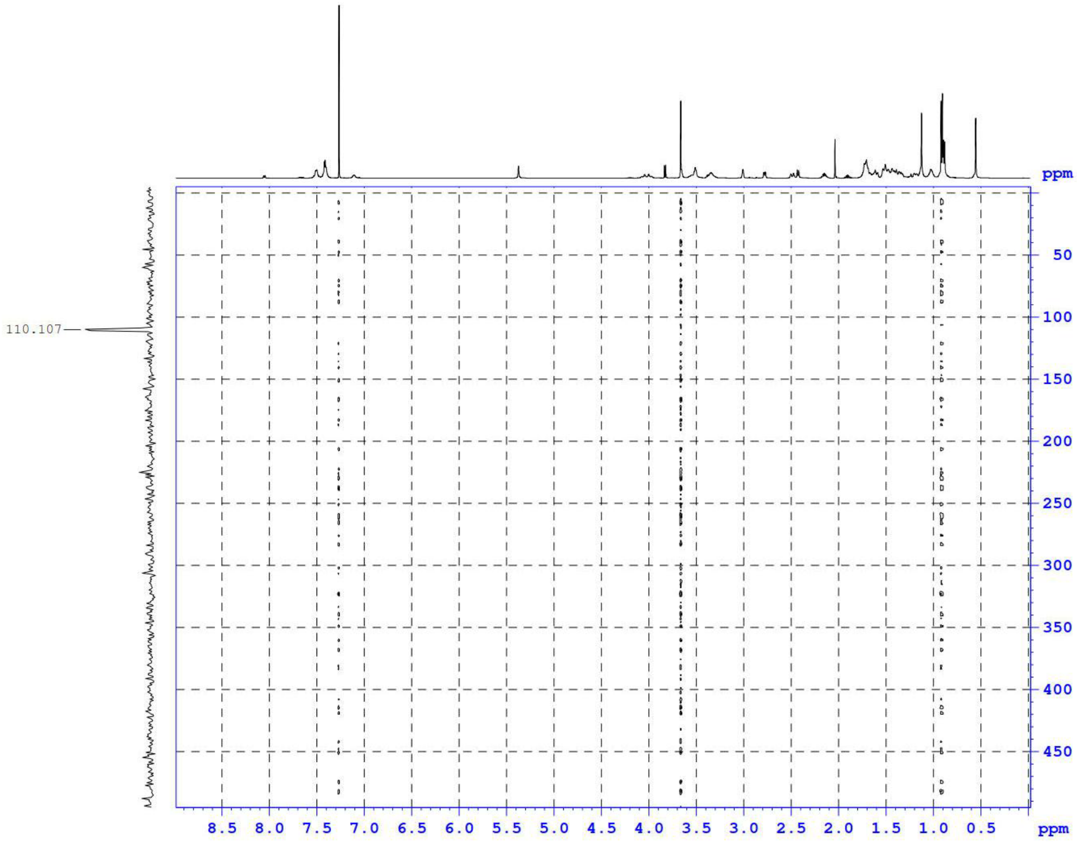

Figure S41. ^{1}H-^{1}H NOESY spectrum of compound **9** in $CDCl_3$

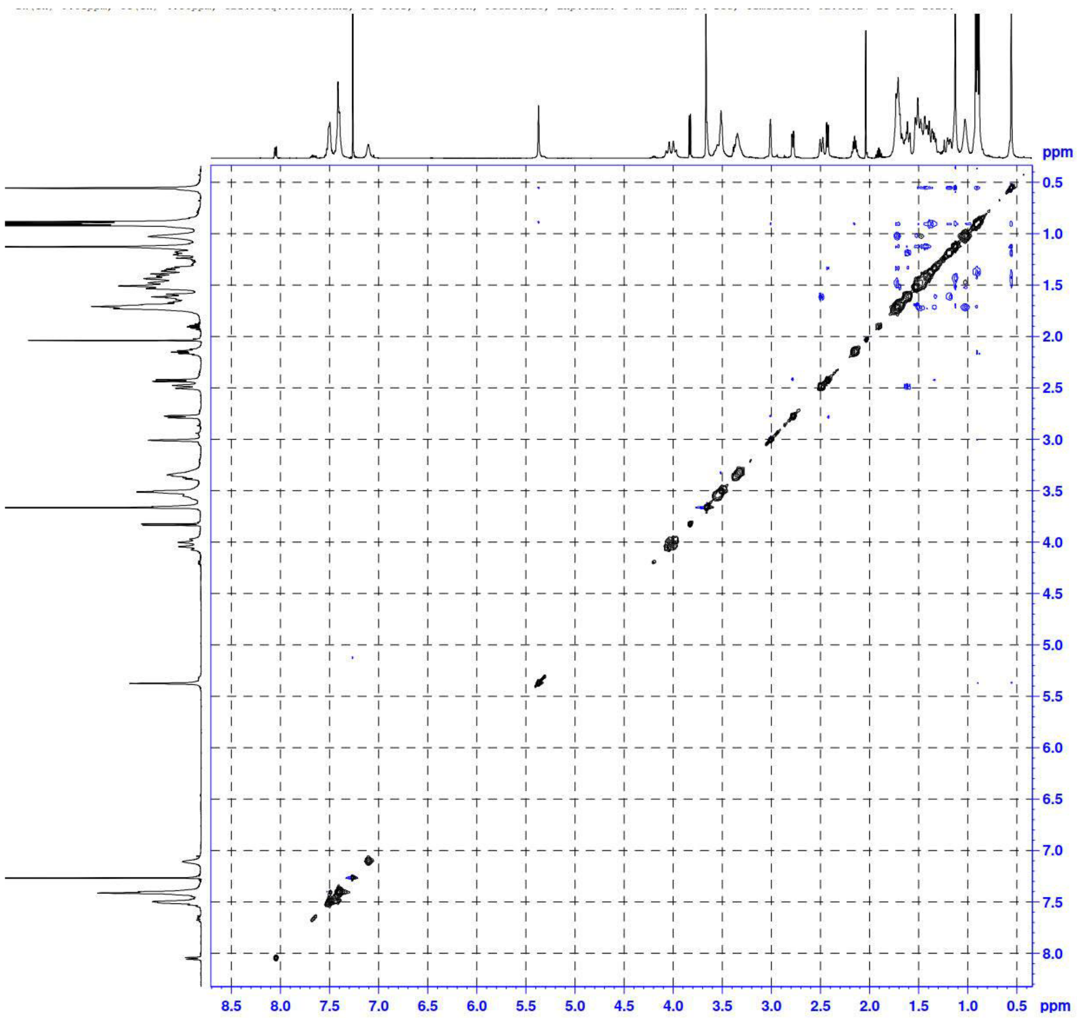

Figure S42. ^{1}H NMR spectrum of compound **10** in $CDCl_3$ (500 MHz)

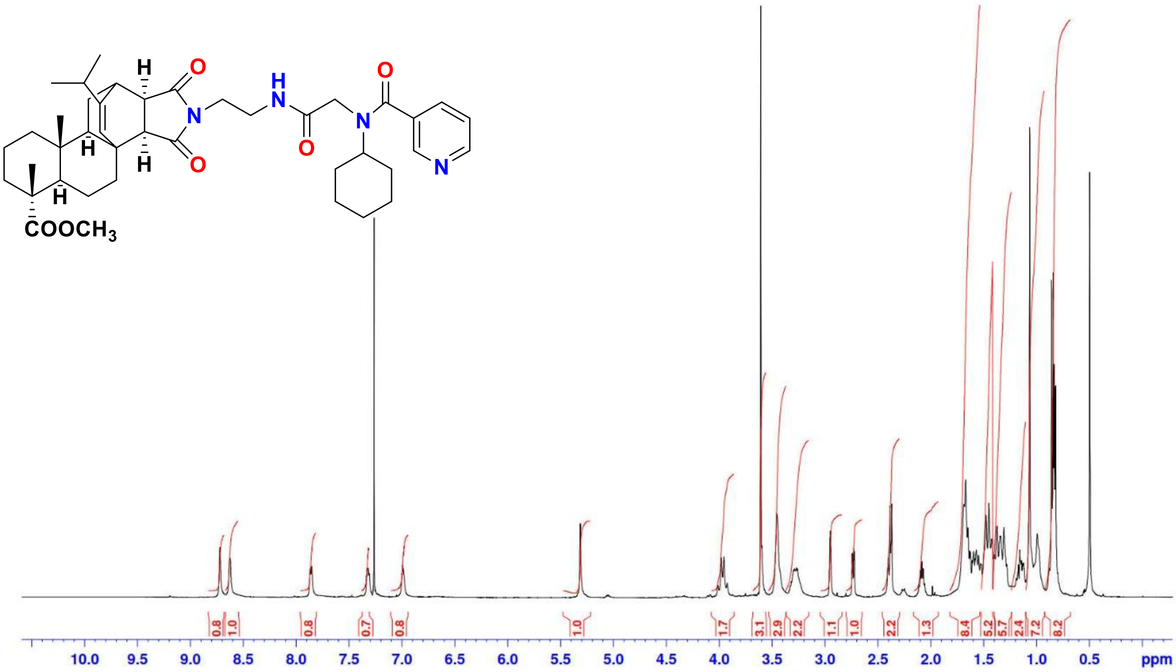

Figure S43. ^{13}C NMR spectrum of compound **10** in $CDCl_3$ (125 MHz)

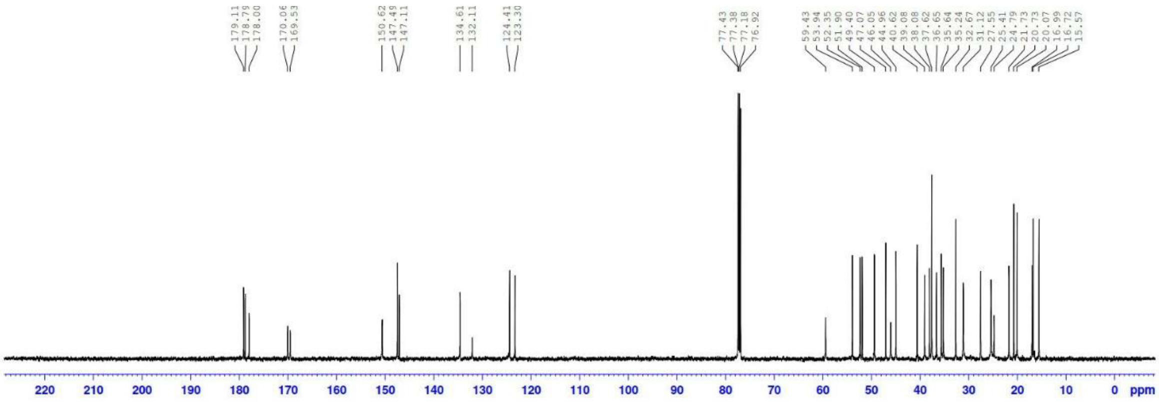

Figure S44. ^{1}H-^{1}H COSY spectrum of compound **10** in $CDCl_3$

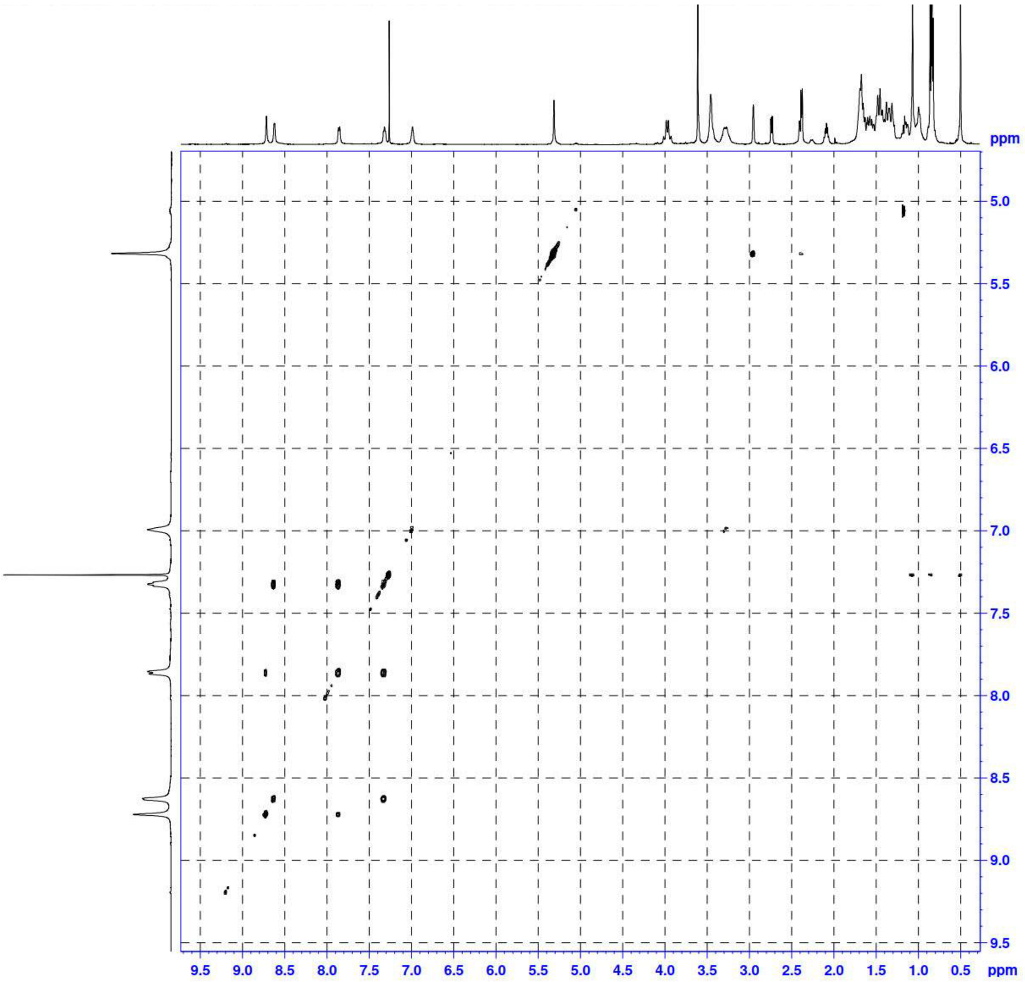

Figure S45. ^{1}H-^{13}C HSQC spectrum of compound **10** in $CDCl_3$

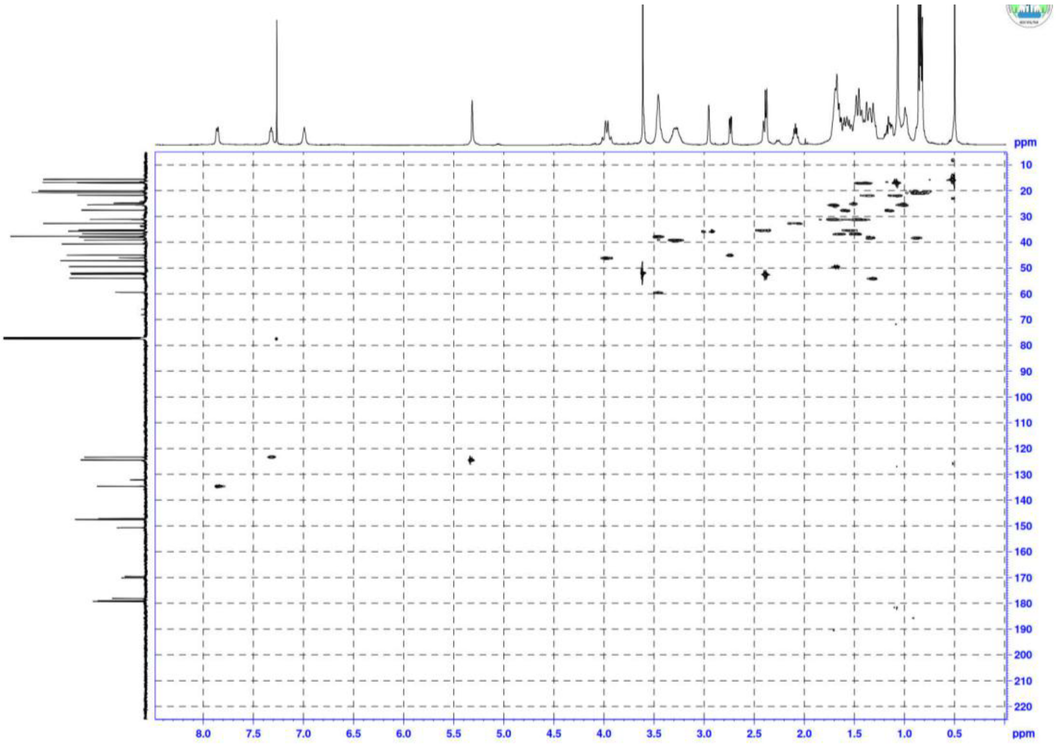

Figure S46. ^{1}H-^{13}C HMBC spectrum of compound **10** in $CDCl_3$

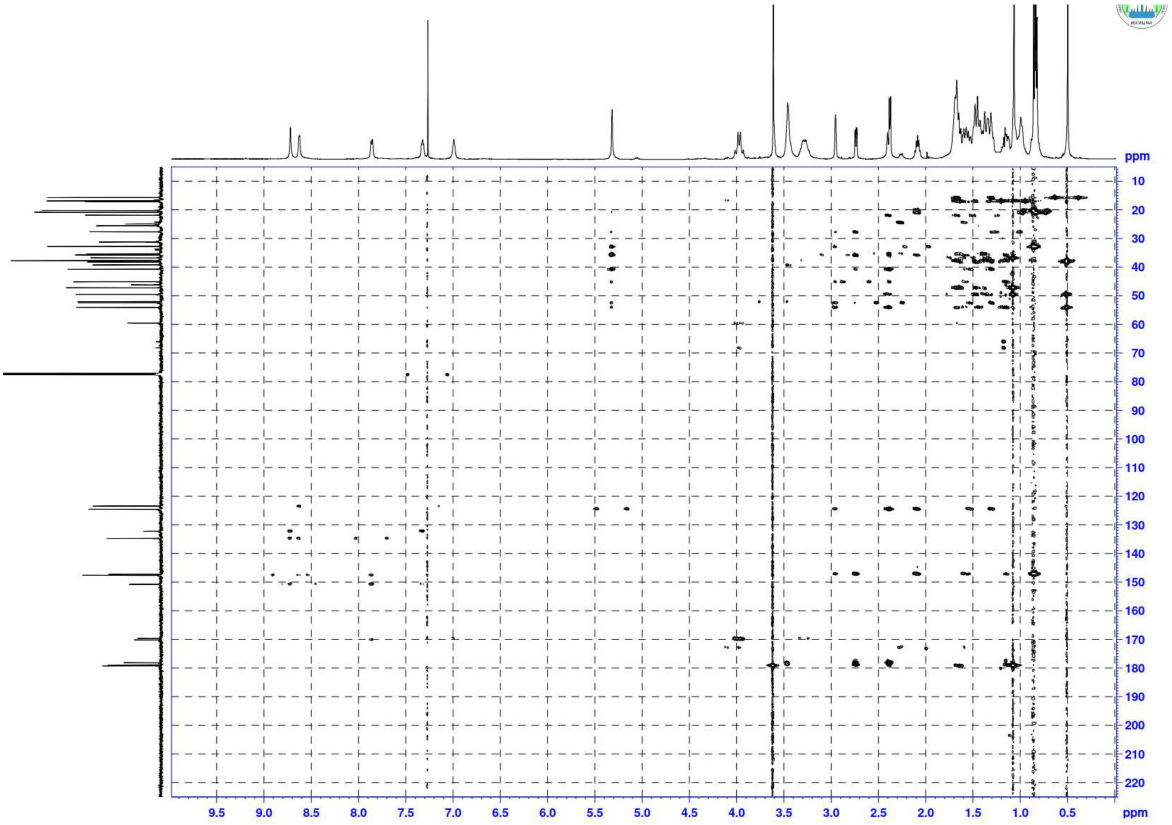

Figure S47. ^{1}H-^{1}H NOESY spectrum of compound **10** in $CDCl_3$

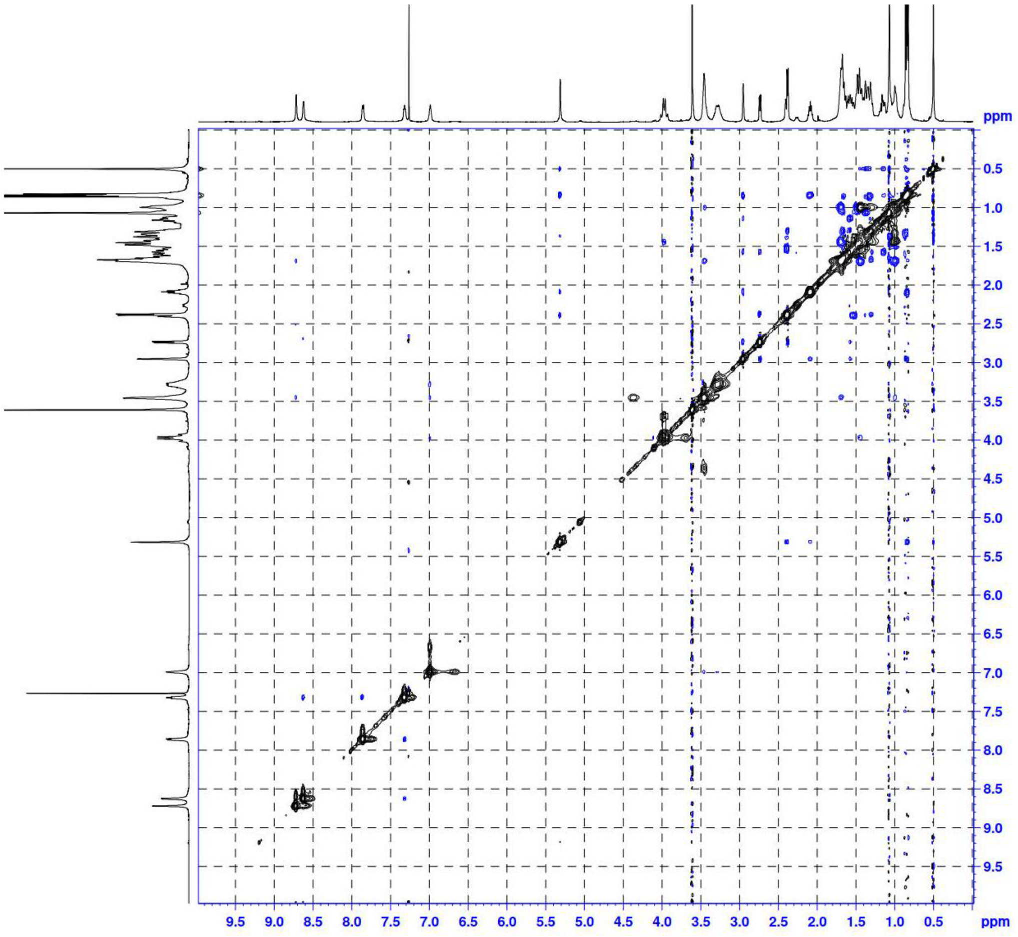

Figure S48. ^{1}H NMR spectrum of compound **11** in $CDCl_3$ (500 MHz)

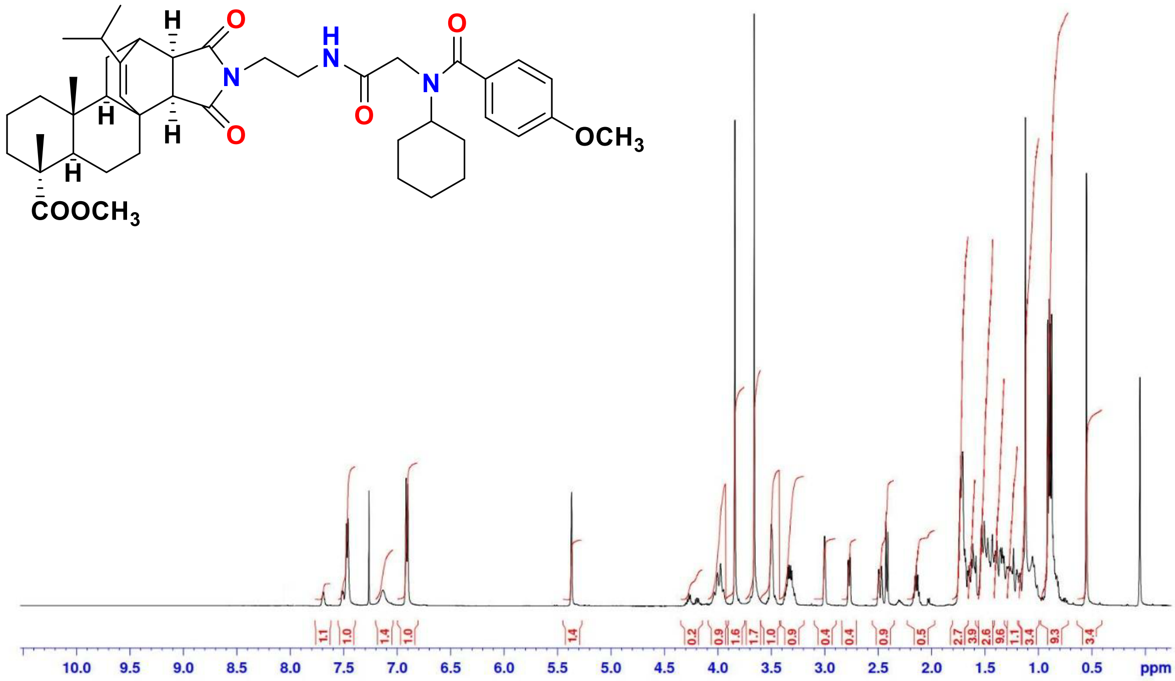

Figure S49. ^{13}C NMR spectrum of compound **11** in $CDCl_3$ (125MHz)

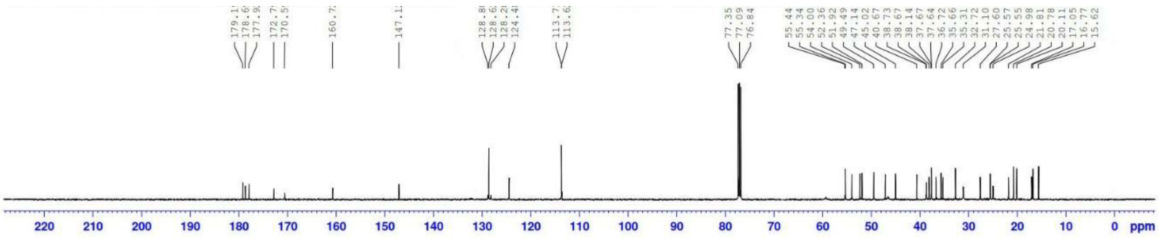

Figure S50. ^1H-^1H COSY spectrum of compound **11** in $CDCl_3$

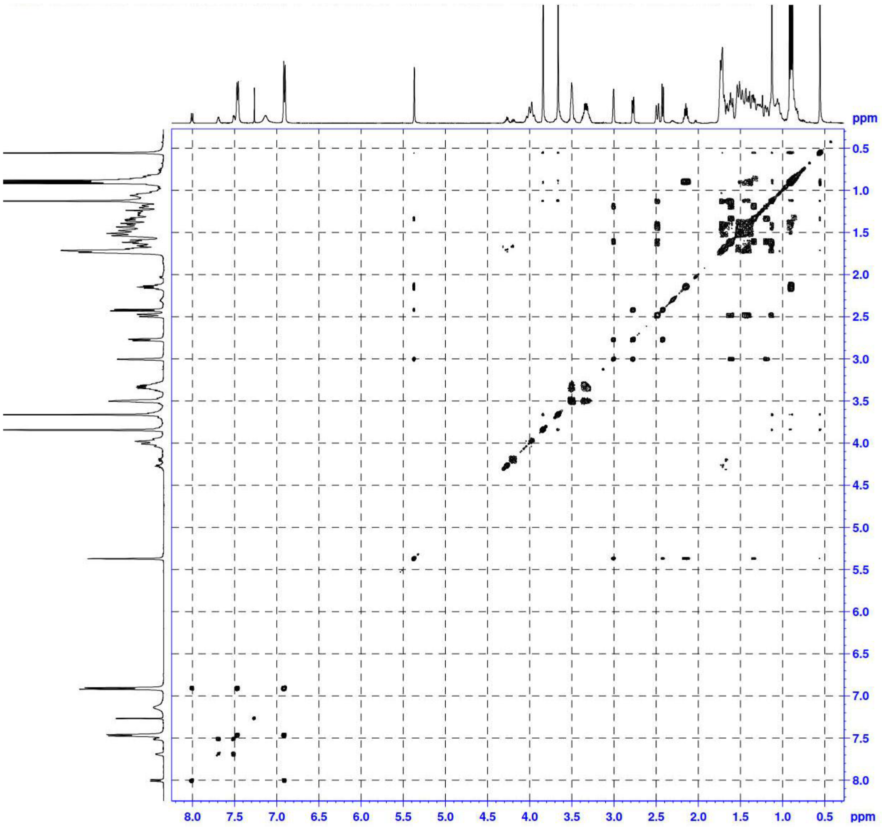

Figure S51. NOESY spectrum of compound **11** in $CDCl_3$

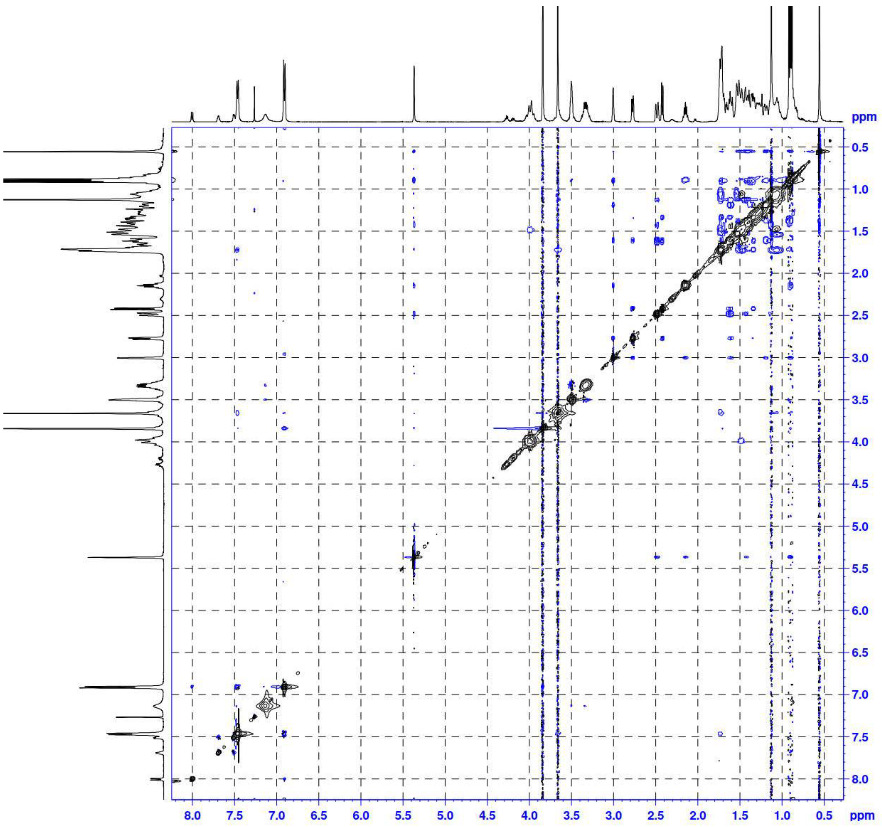

Figure S52. ^{1}H-^{13}C HSQC spectrum of compound **11** in $CDCl_3$

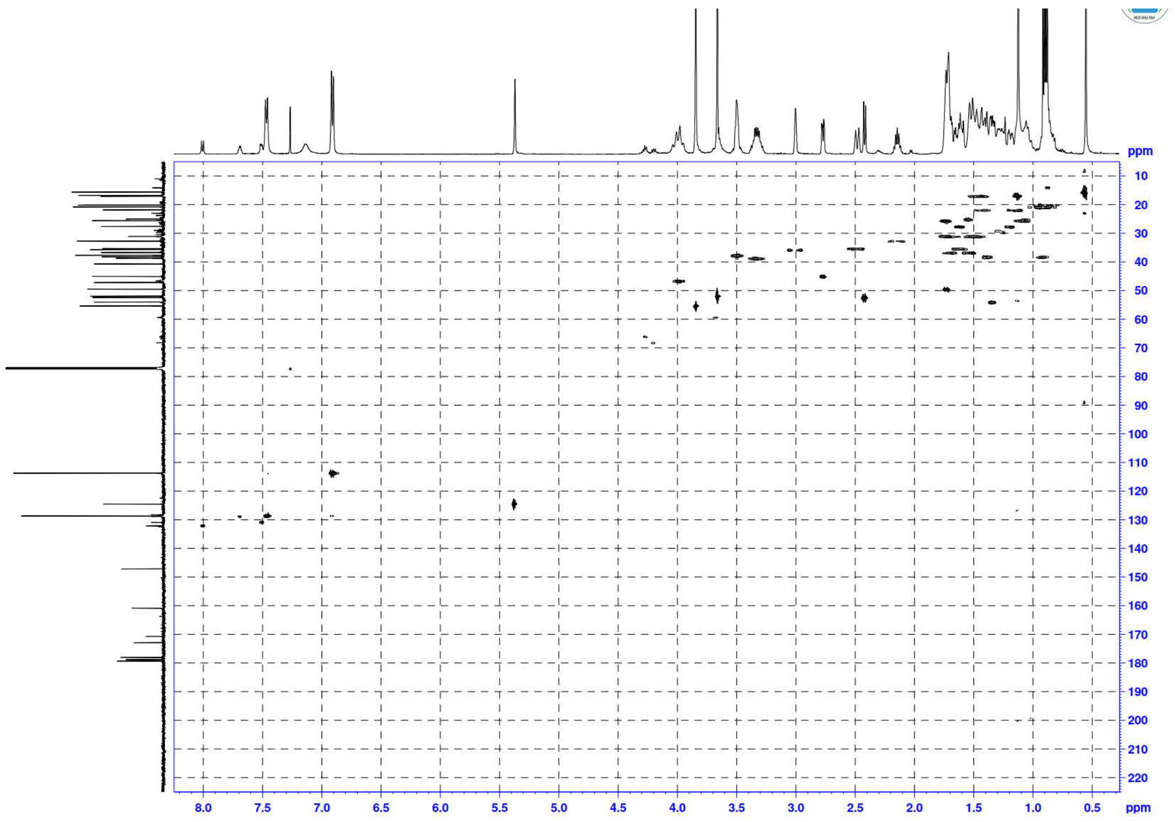

Figure S53. ^{1}H-^{13}C HMBC spectrum of compound **11** in $CDCl_3$

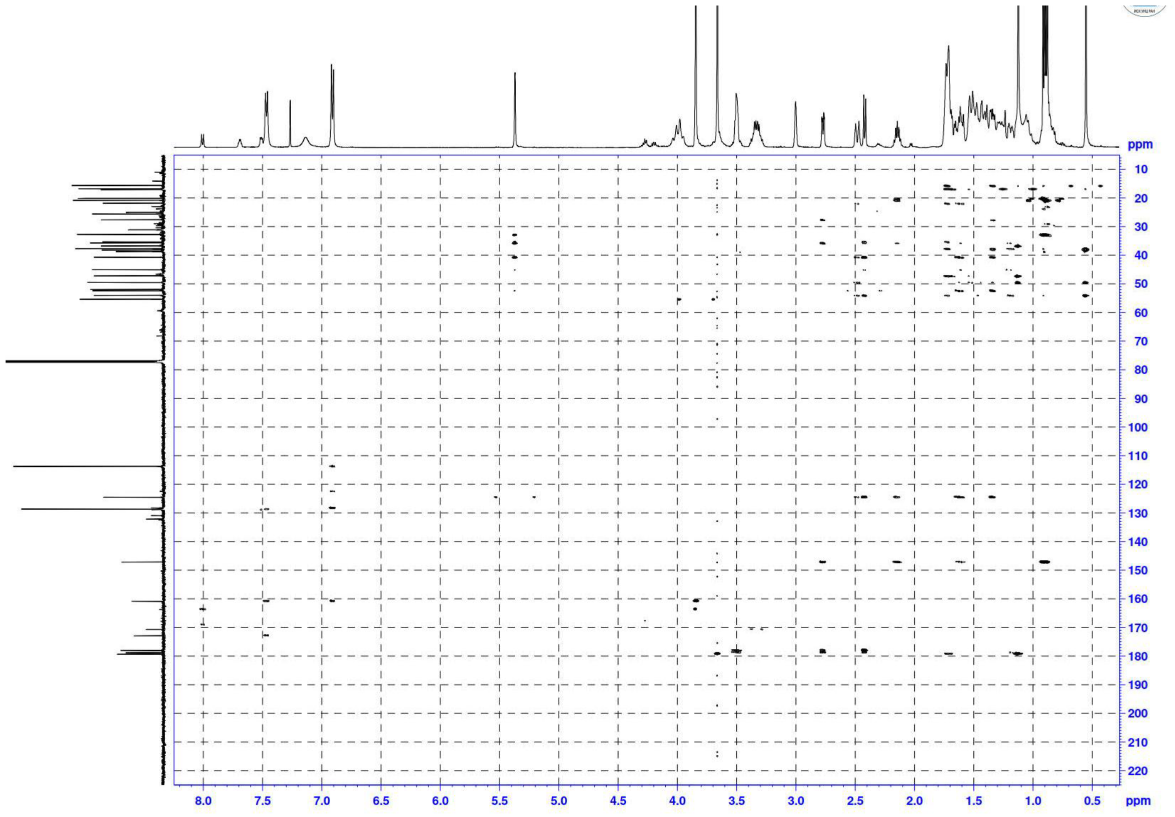

Figure S54. ^{1}H-^{15}N HMBC spectrum of compound **11** in $CDCl_3$

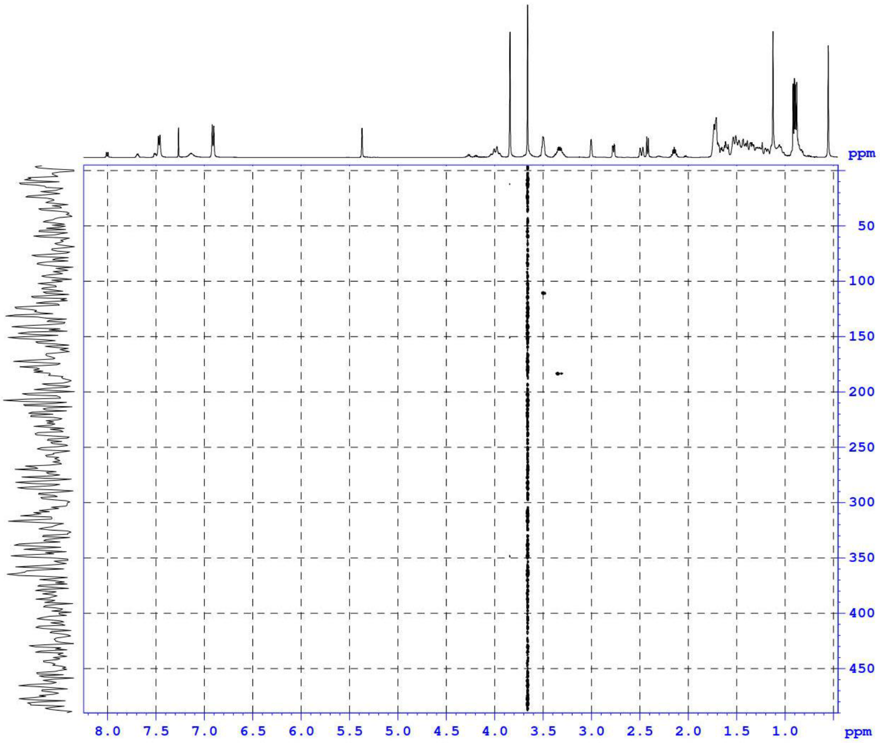

Figure S55. ^{1}H NMR spectrum of compound **12** in $CDCl_3$ (500 MHz)

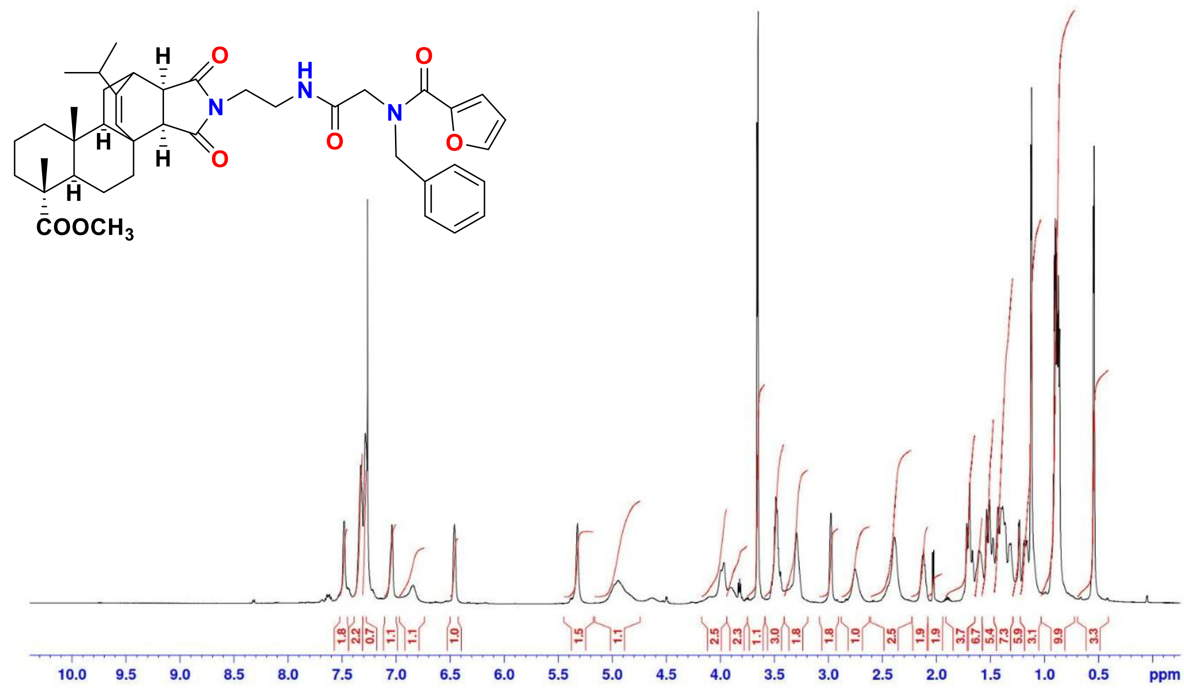

Figure S56. ^{13}C NMR spectrum of compound **12** in $CDCl_3$ (125MHz)

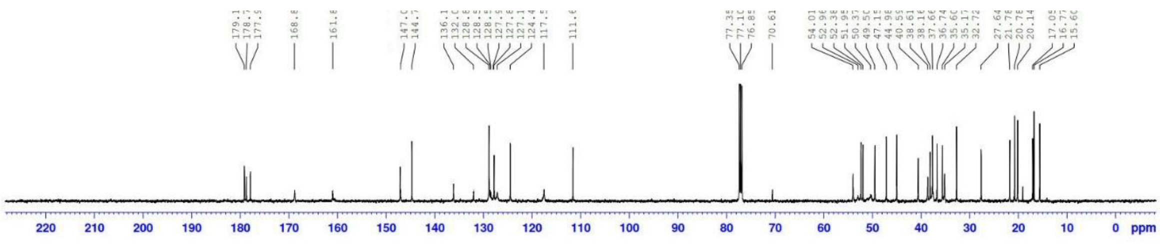

Figure S57. ^{1}H-^{1}H COSY spectrum of compound **12** in $CDCl_3$

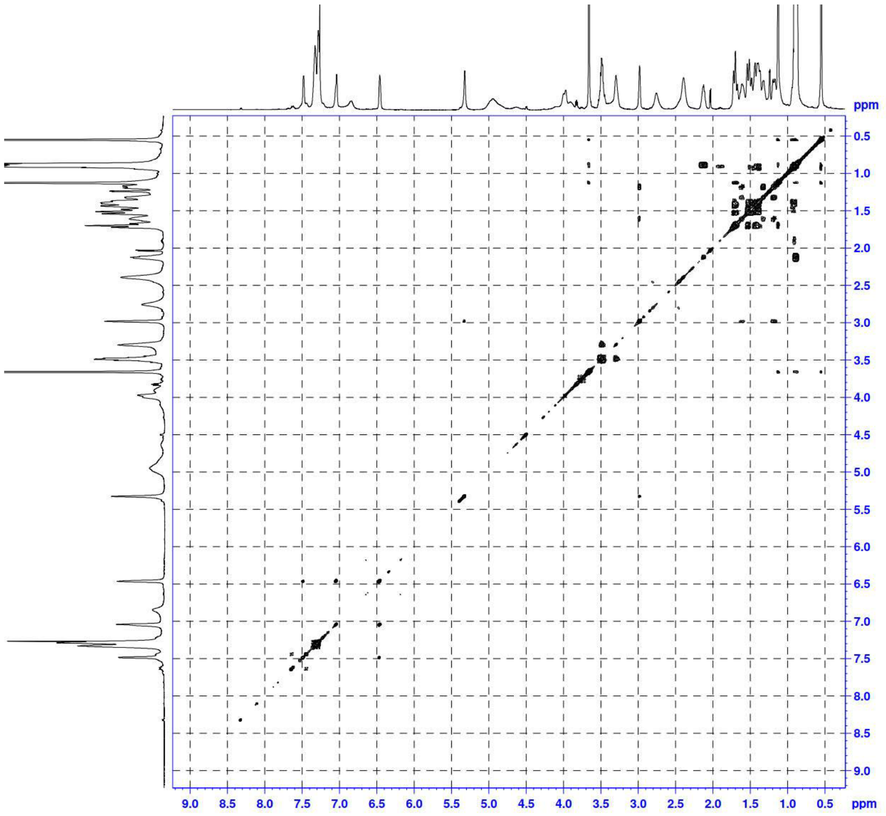

Figure S58. NOESY spectrum of compound **12** in $CDCl_3$

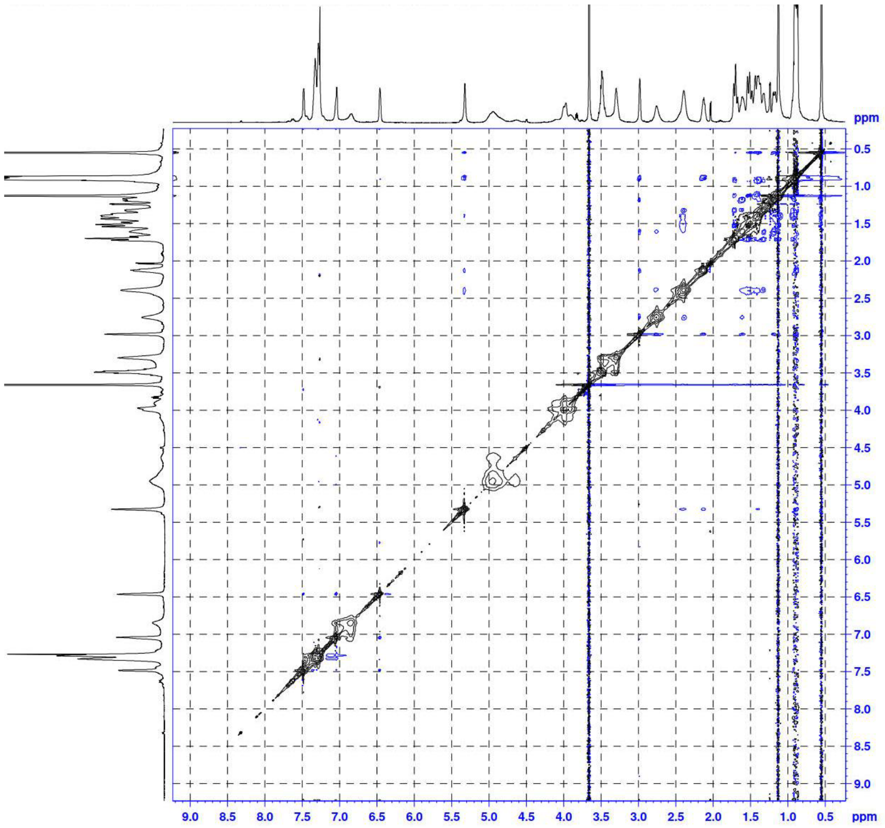

Figure S59. ^{1}H-^{13}C HSQC spectrum of compound **12** in $CDCl_3$

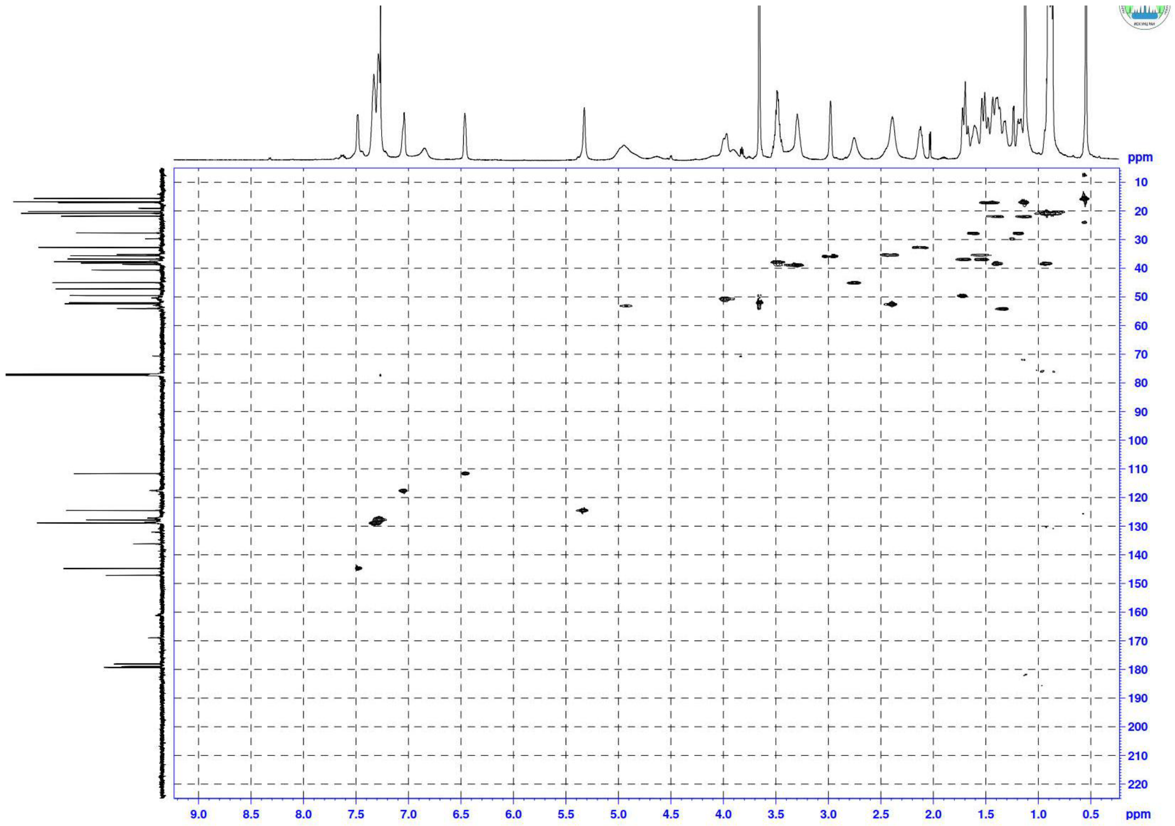

Figure S60. ^{1}H-^{13}C HMBC spectrum of compound **12** in $CDCl_3$

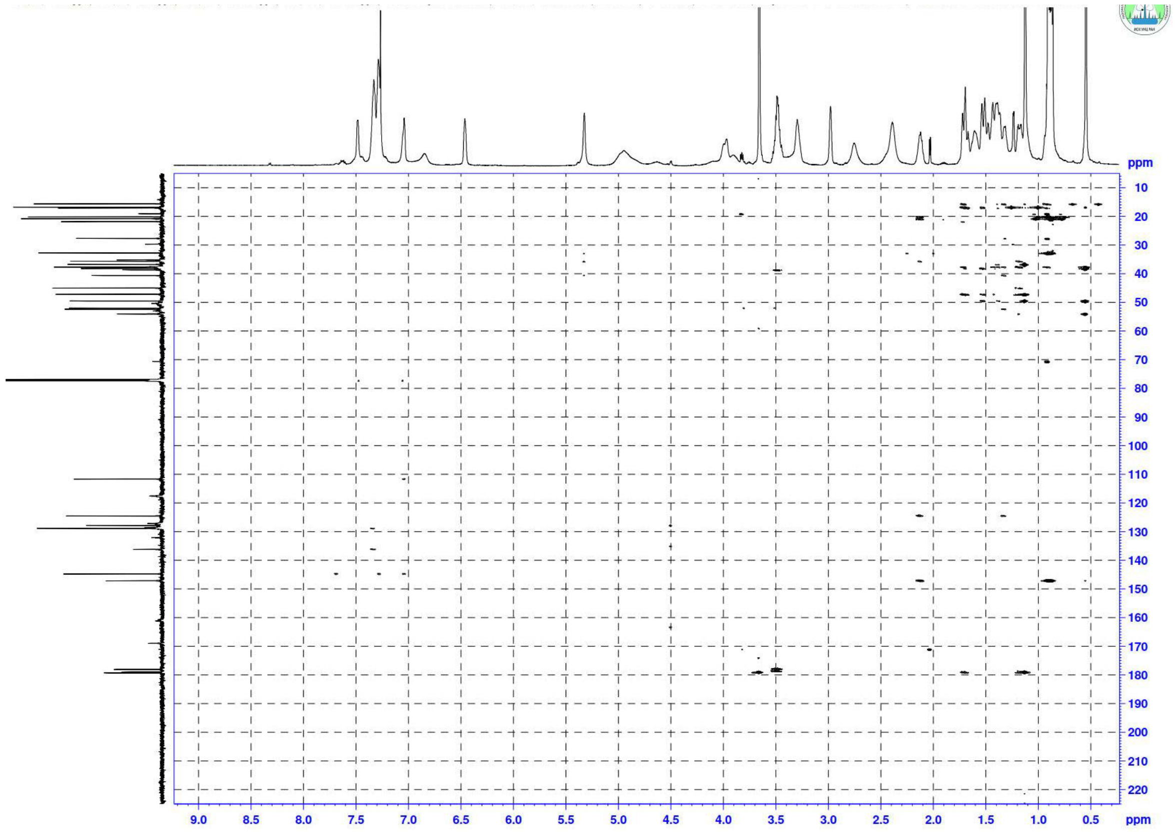

Figure S61. ^{1}H-^{15}N HSQC spectrum of compound **12** in $CDCl_3$

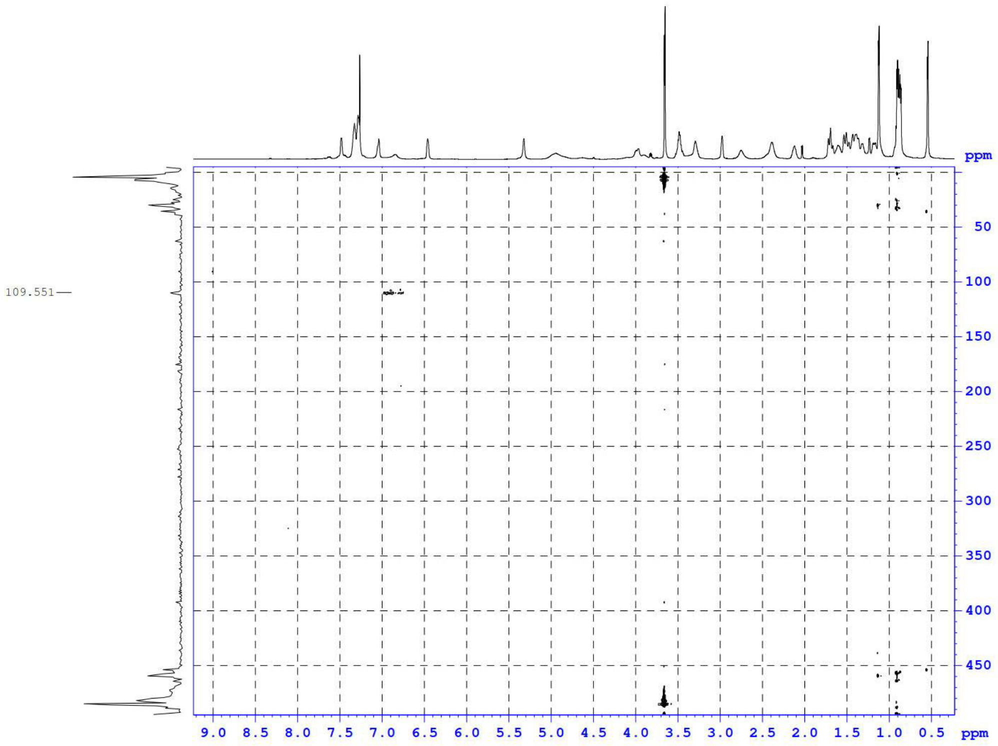

Figure S62. ^{1}H-^{15}N HMBC spectrum of compound **12** in $CDCl_3$

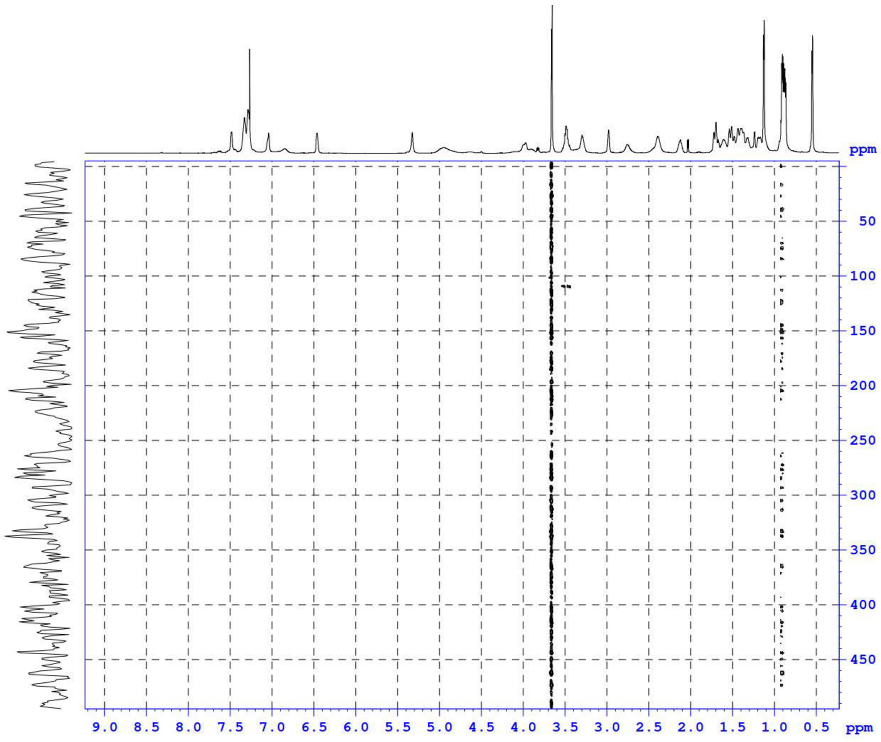

Figure S63. ^{1}H NMR spectrum of compound **13** in $CDCl_3$ (500 MHz)

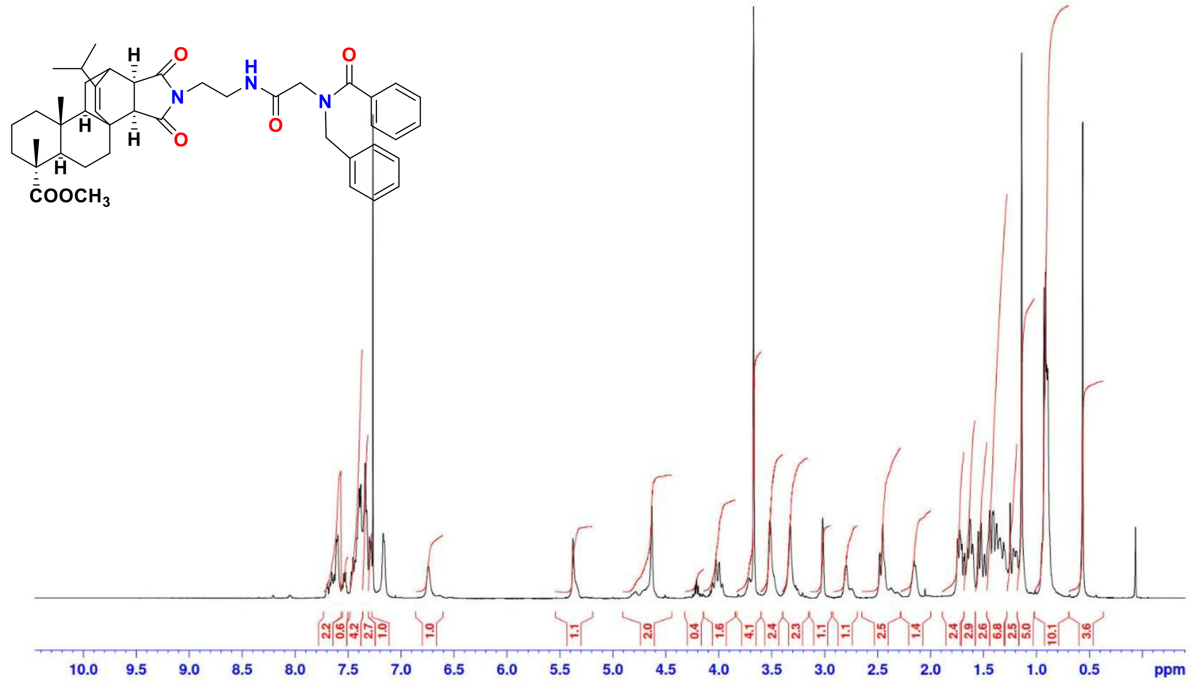

Figure S64. ^{13}C NMR spectrum of compound **13** in $CDCl_3$ (125MHz)

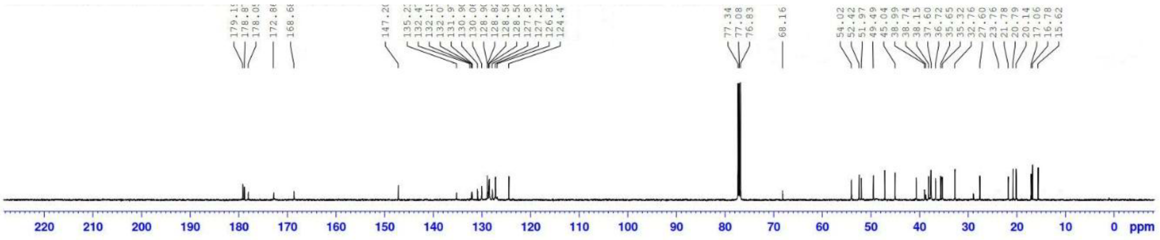

Figure S65. ^1H-^1H COSY spectrum of compound **13** in $CDCl_3$

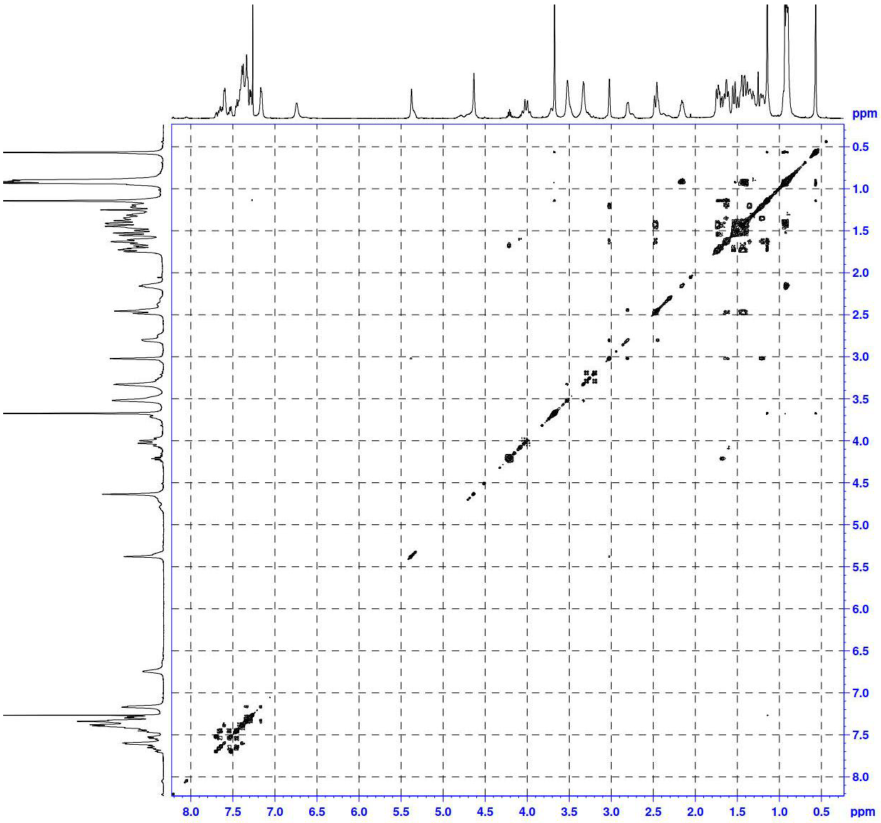

Figure S66. NOESY spectrum of compound **13** in $CDCl_3$

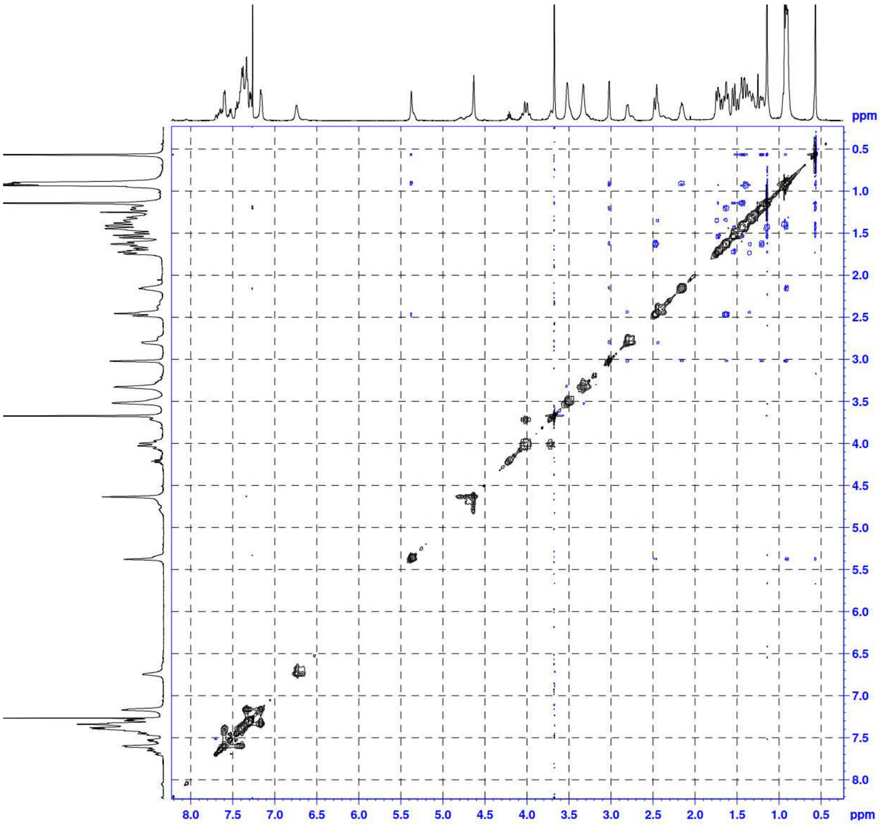

Figure S67. ^{1}H-^{13}C HSQC spectrum of compound **13** in $CDCl_3$

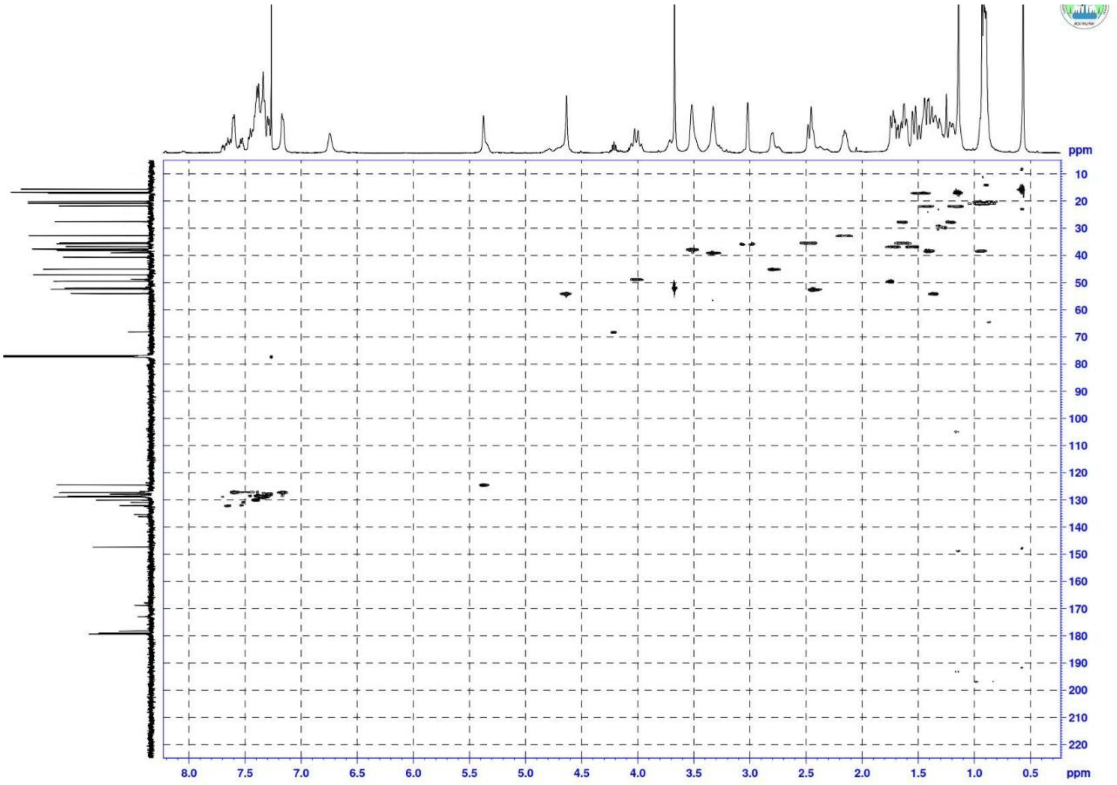

Figure S68. ^{1}H-^{13}C HMBC spectrum of compound **13** in $CDCl_3$

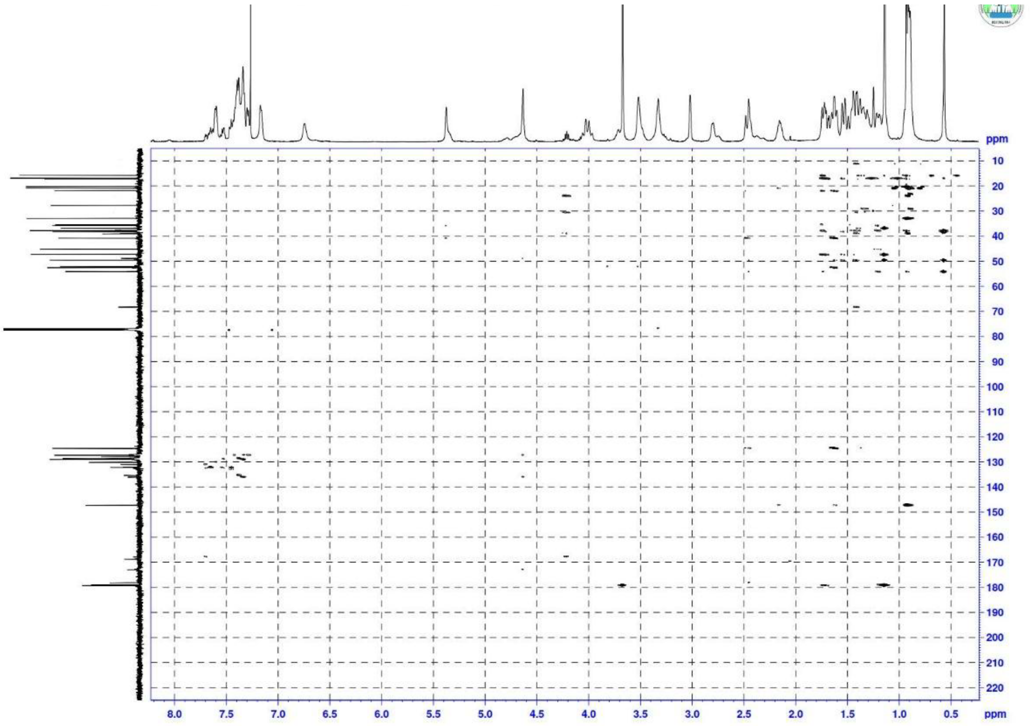

Figure S69. ^{1}H-^{15}N HSQC spectrum of compound **13** in $CDCl_3$

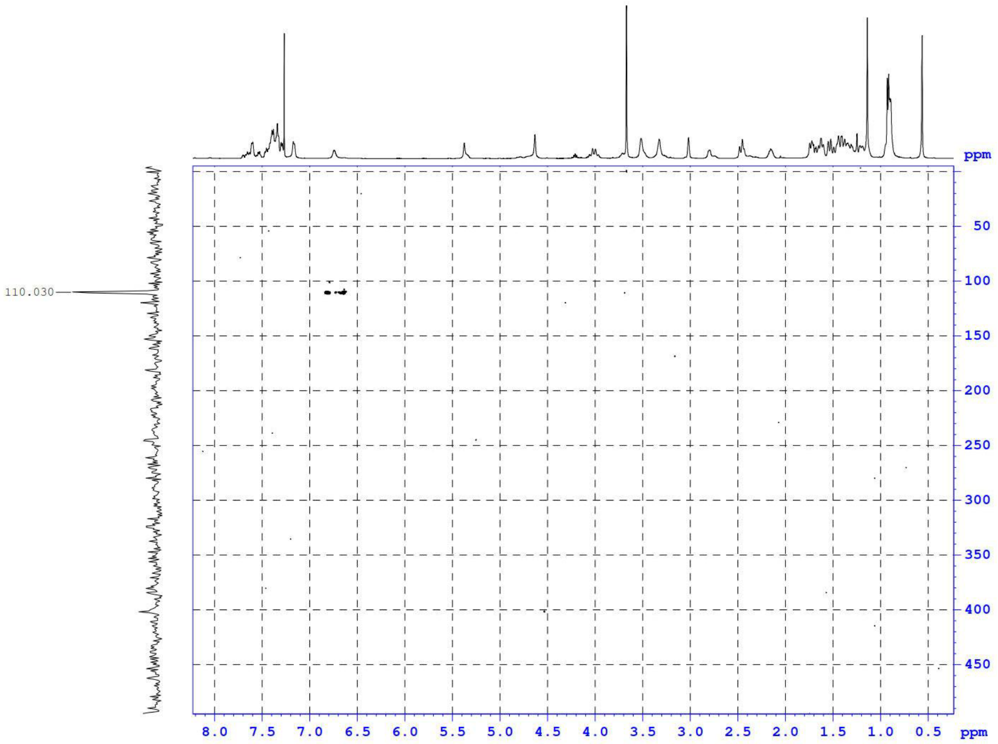

Figure S70. ^{1}H NMR spectrum of compound **14** in $CDCl_3$ (500 MHz)

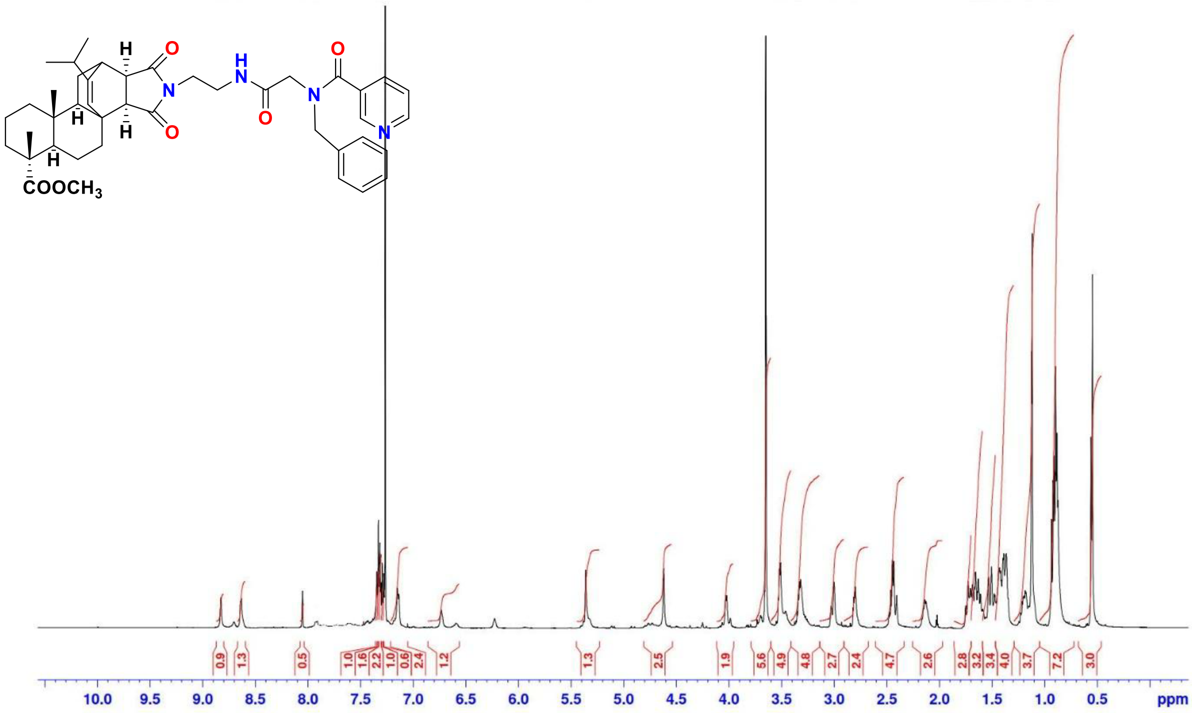

Figure S71. ^{13}C NMR spectrum of compound **14** in $CDCl_3$ (125MHz)

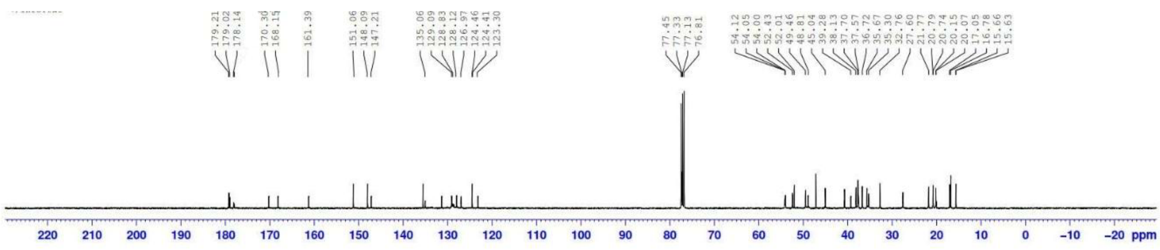

Figure S72. ^1H-^1H COSY spectrum of compound **14** in $CDCl_3$

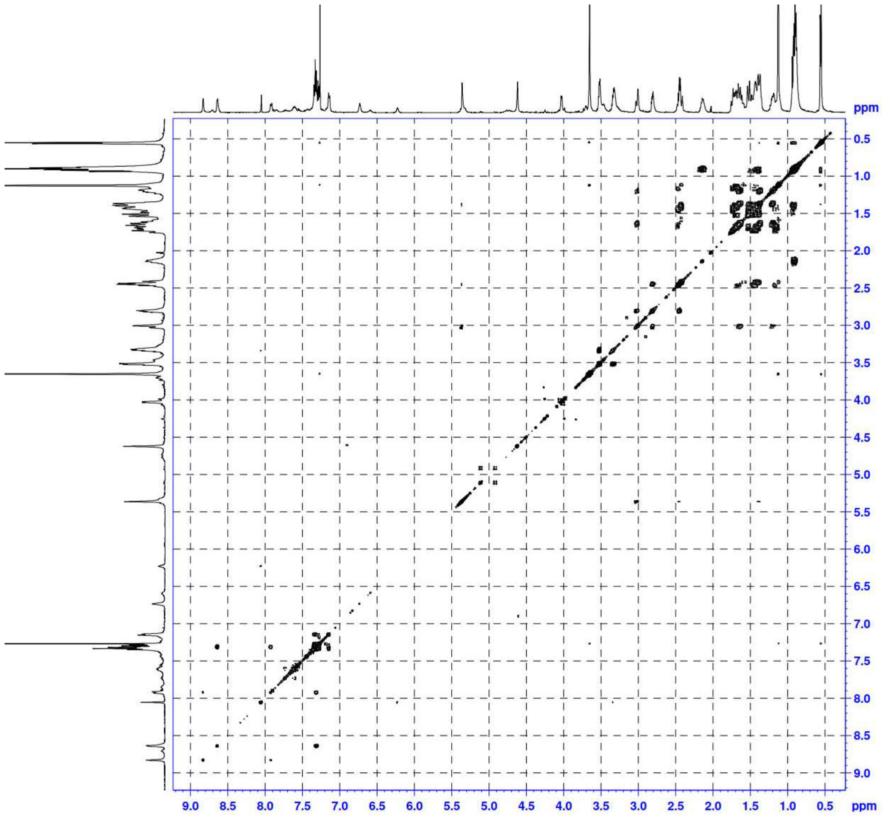

Figure S73. NOESY spectrum of compound **14** in $CDCl_3$

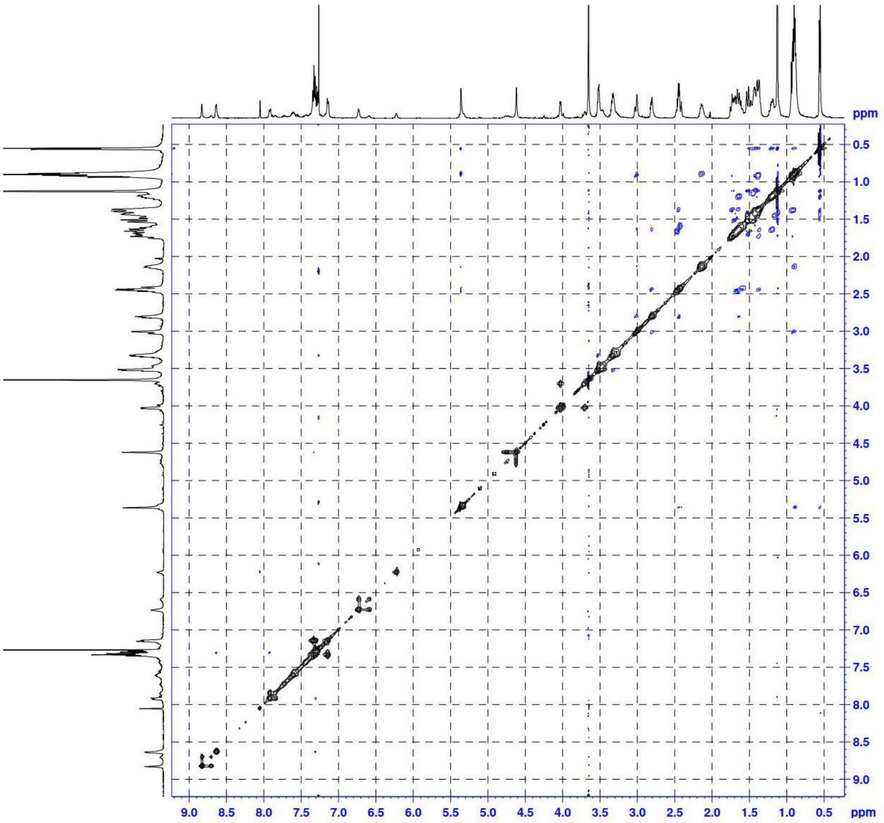

Figure S74. ^{1}H-^{13}C HSQC spectrum of compound **14** in $CDCl_3$

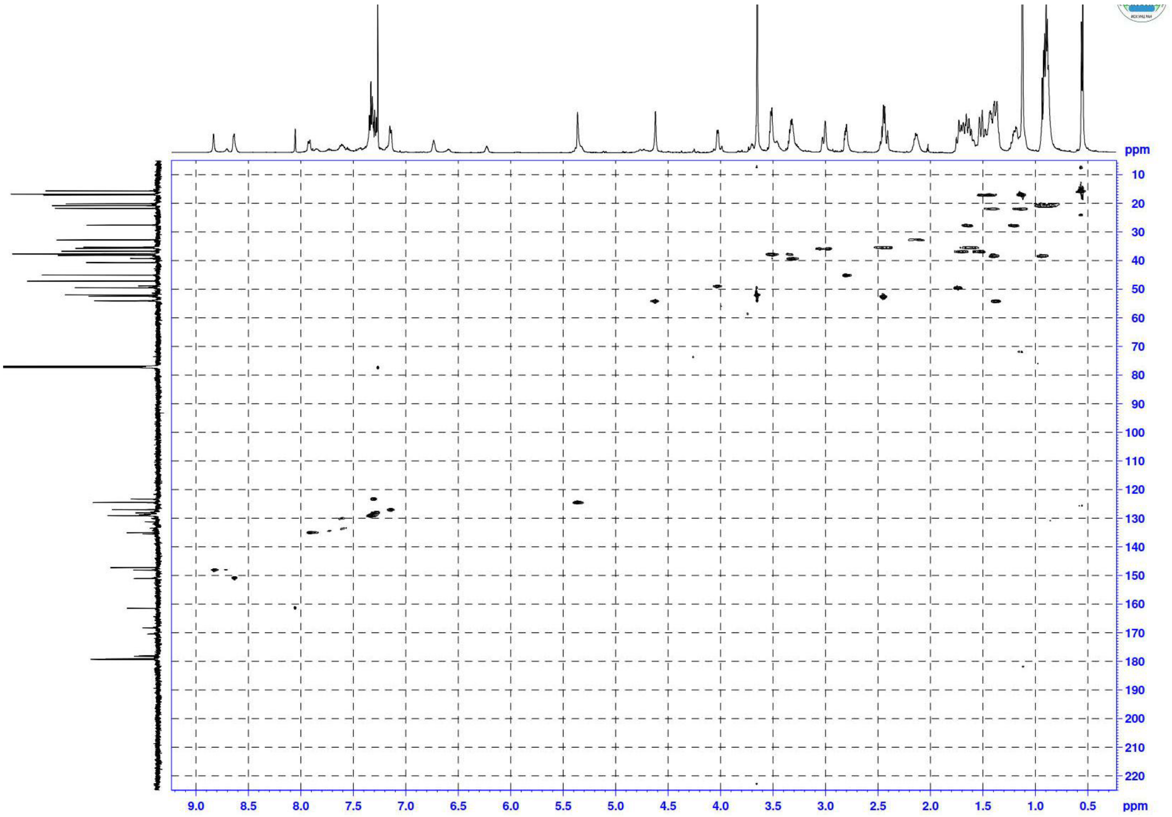

Figure S75. ^{1}H-^{13}C HMBC spectrum of compound **14** in $CDCl_3$

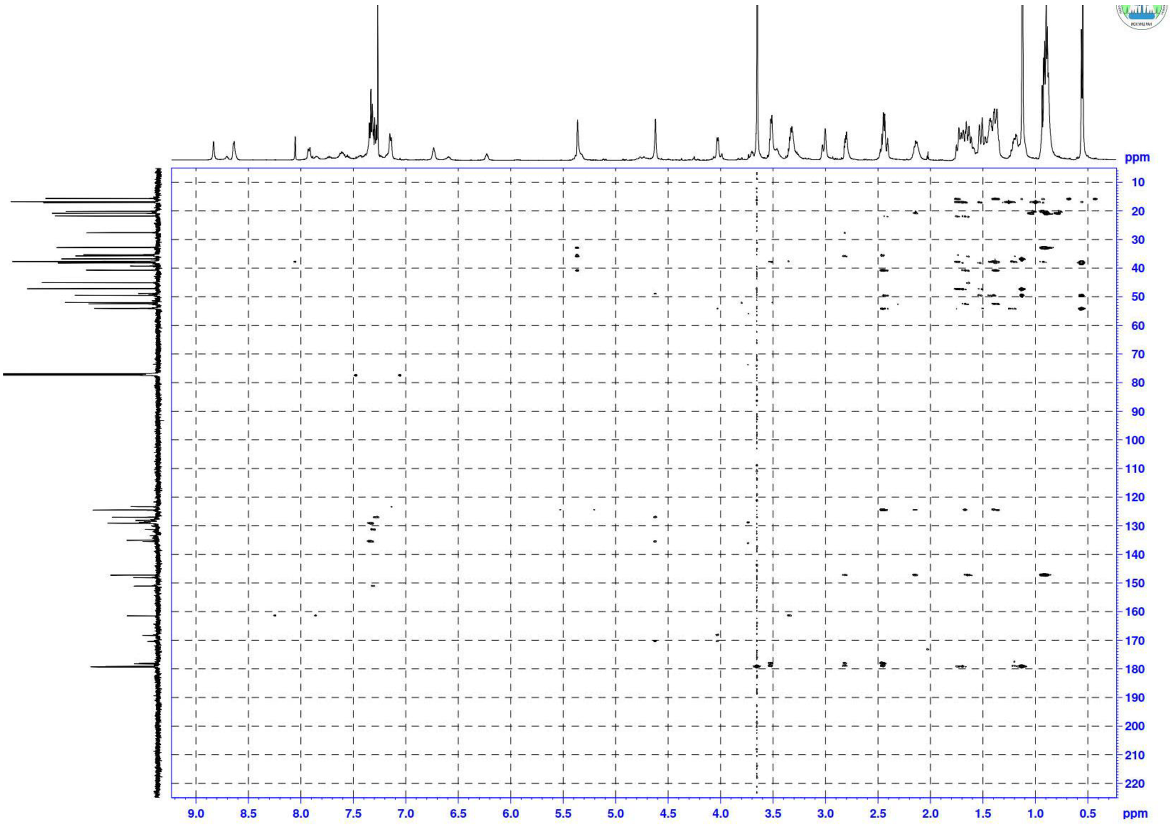

Figure S76. ^{1}H-^{15}N HMBC spectrum of compound **14** in $CDCl_3$

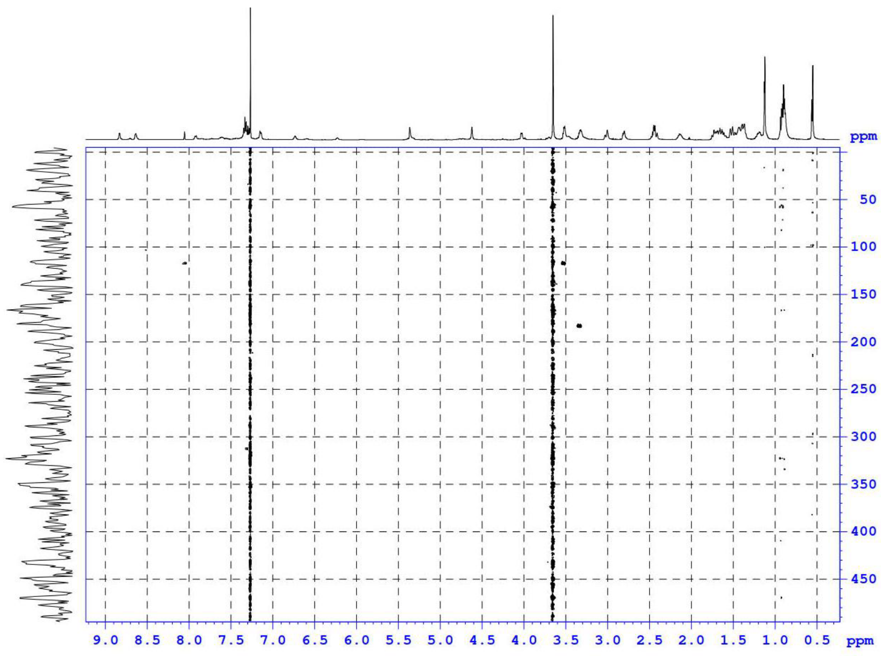

Figure S77. ^{1}H NMR spectrum of compound **15** in $CDCl_3$ (500 MHz)

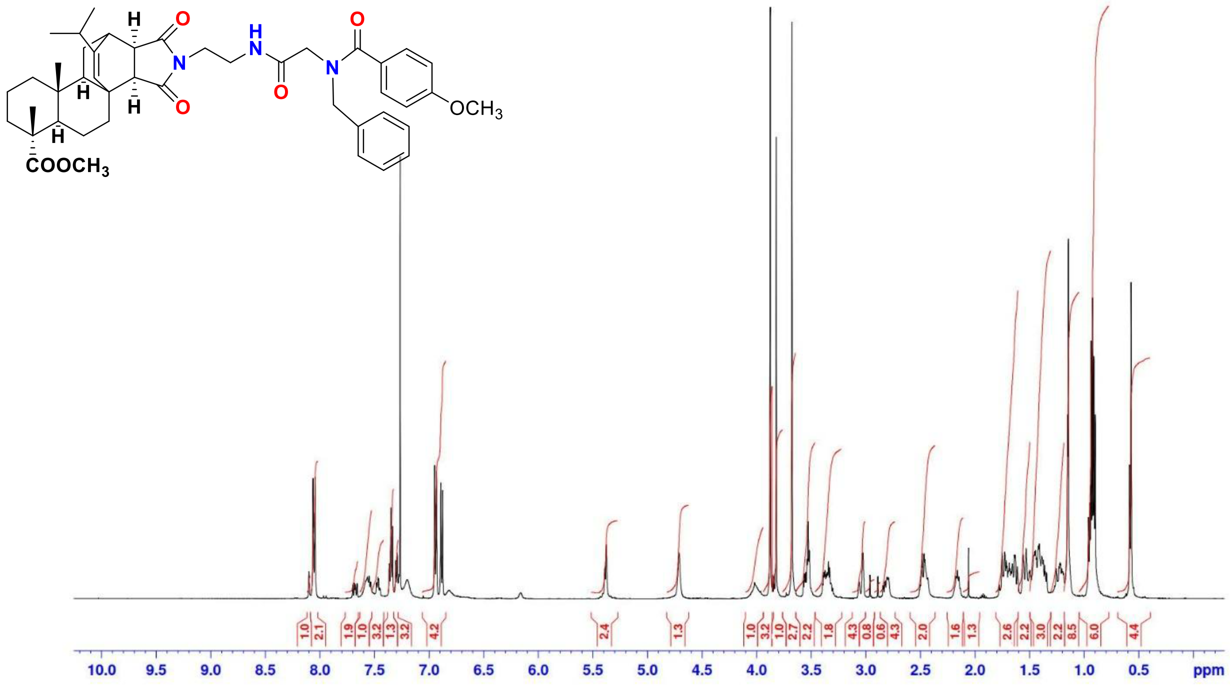

Figure S78. ^{13}C NMR spectrum of compound **15** in $CDCl_3$ (125MHz)

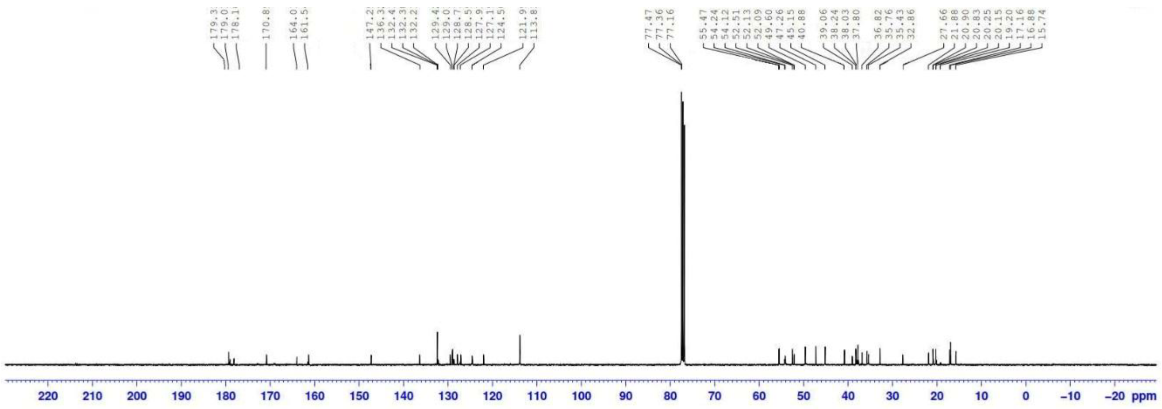

Figure S79. ^1H-^1H COSY spectrum of compound **15** in $CDCl_3$

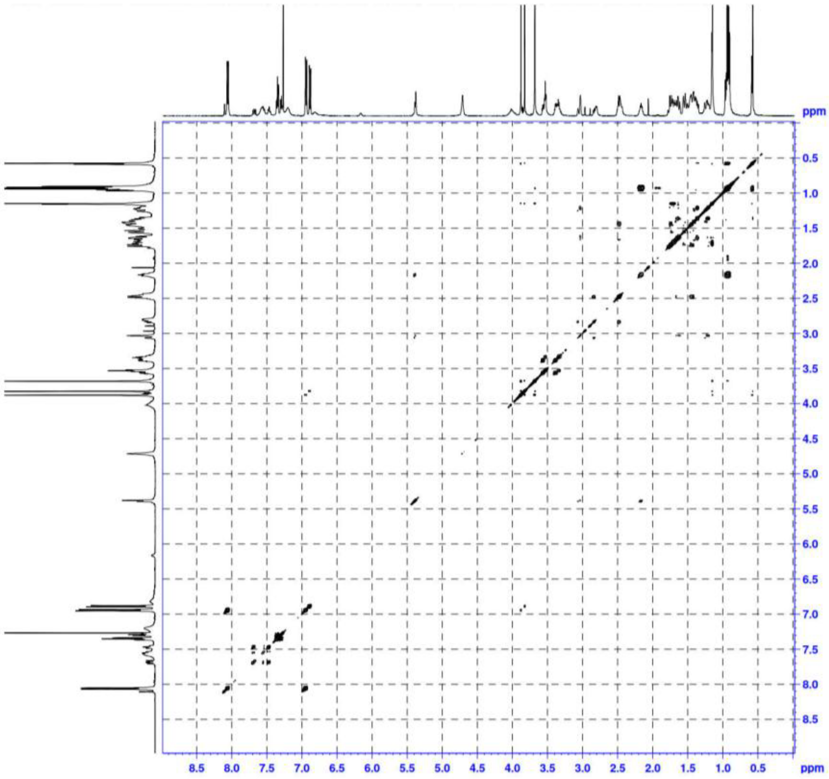

Figure S80. NOESY spectrum of compound **15** in $CDCl_3$

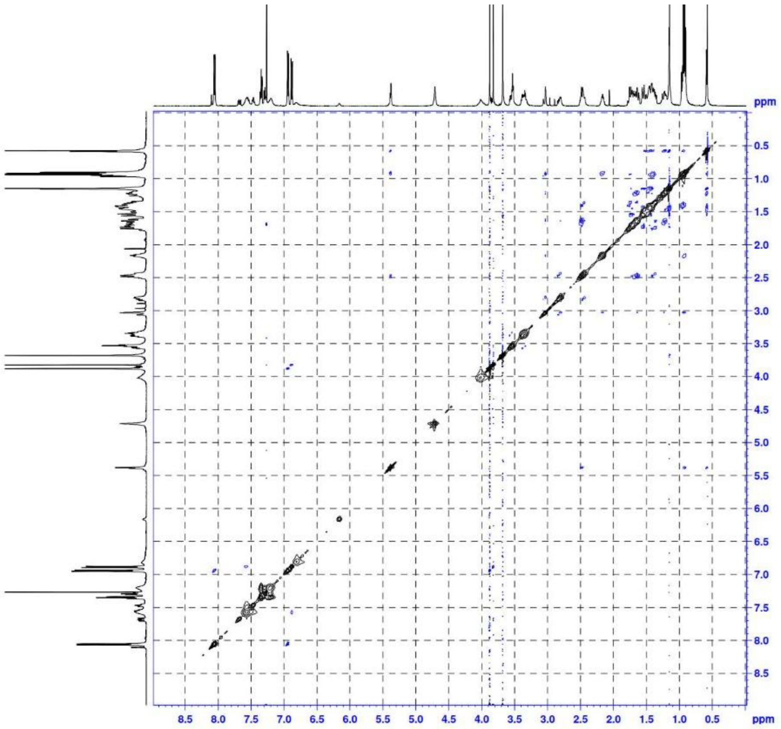

Figure S81. ^{1}H-^{13}C HSQC spectrum of compound **15** in $CDCl_3$

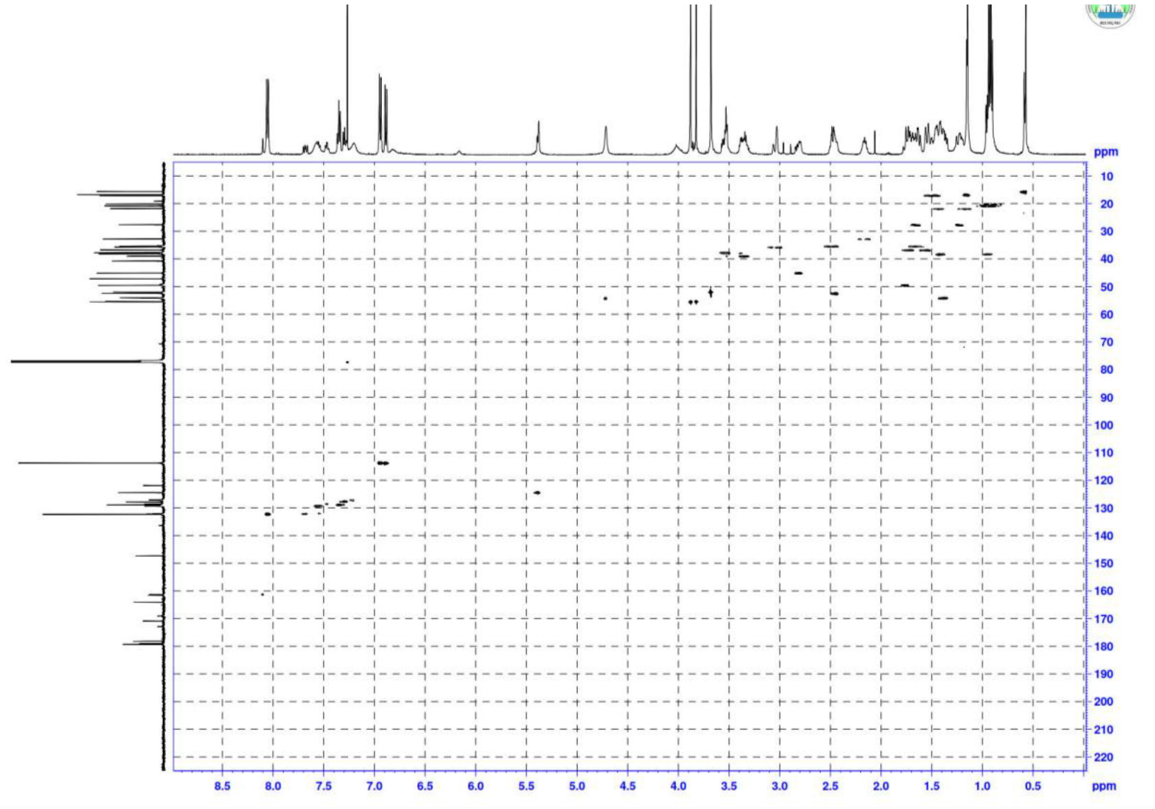

Figure S82. ^{1}H-^{13}C HMBC spectrum of compound **15** in $CDCl_3$

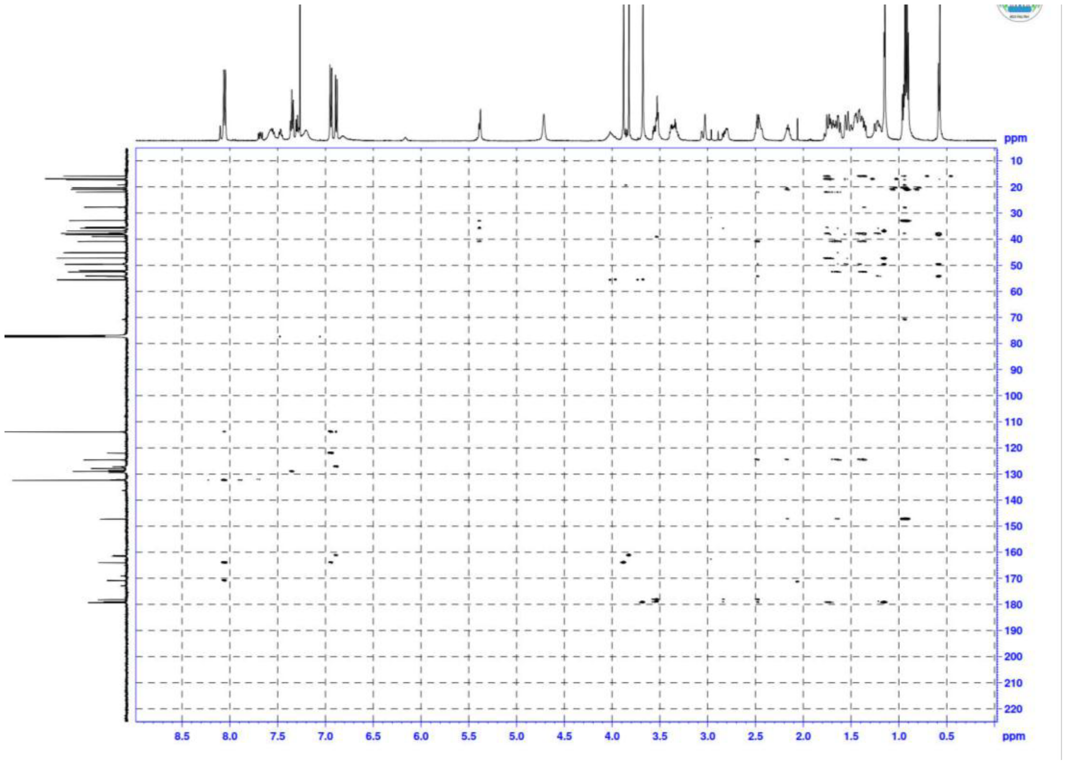

Figure S83. ^{1}H-^{15}N HMBC spectrum of compound **15** in $CDCl_3$

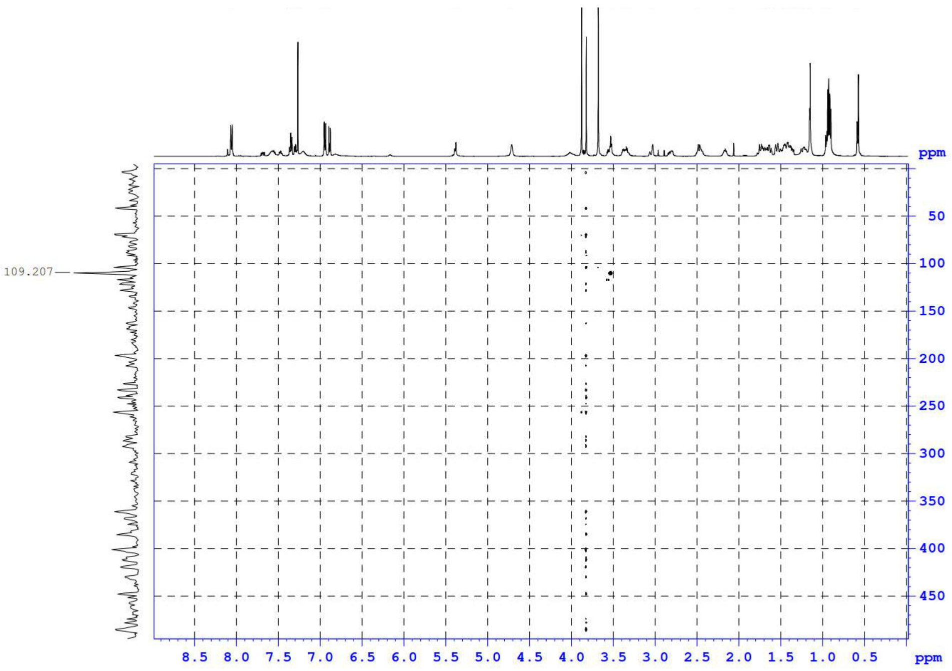

Figure S84. ^{1}H NMR spectrum of compound **16** in $CDCl_3$ (500 MHz)

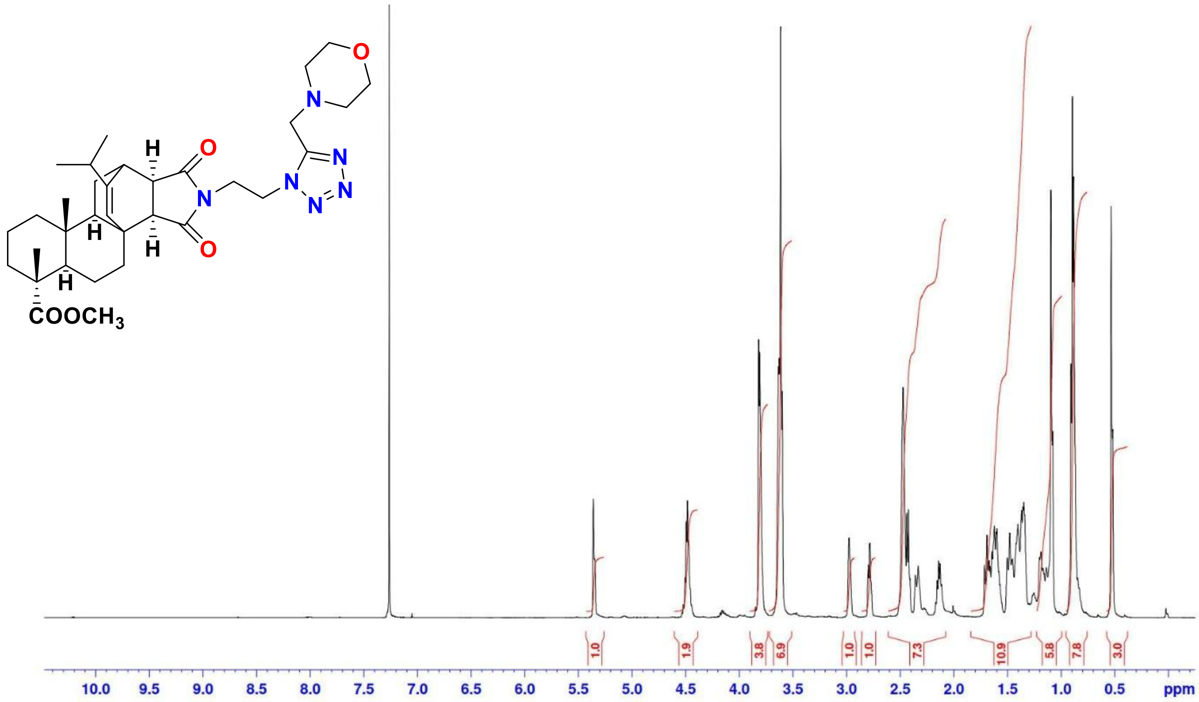

Figure S85. ^{13}C NMR spectrum of compound **16** in $CDCl_3$ (125 MHz)

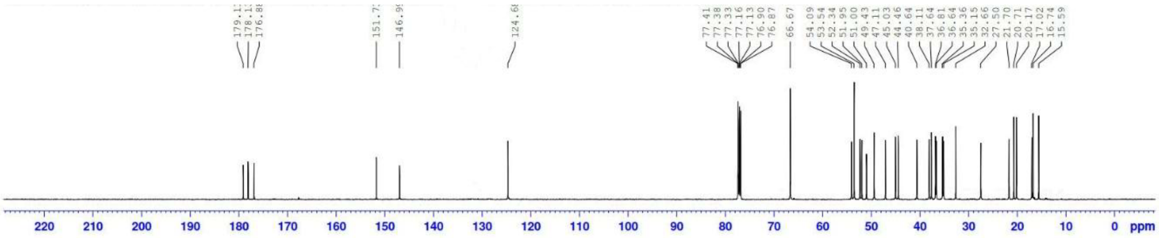

Figure S81. ^1H-^1H COSY spectrum of compound **16** in $CDCl_3$

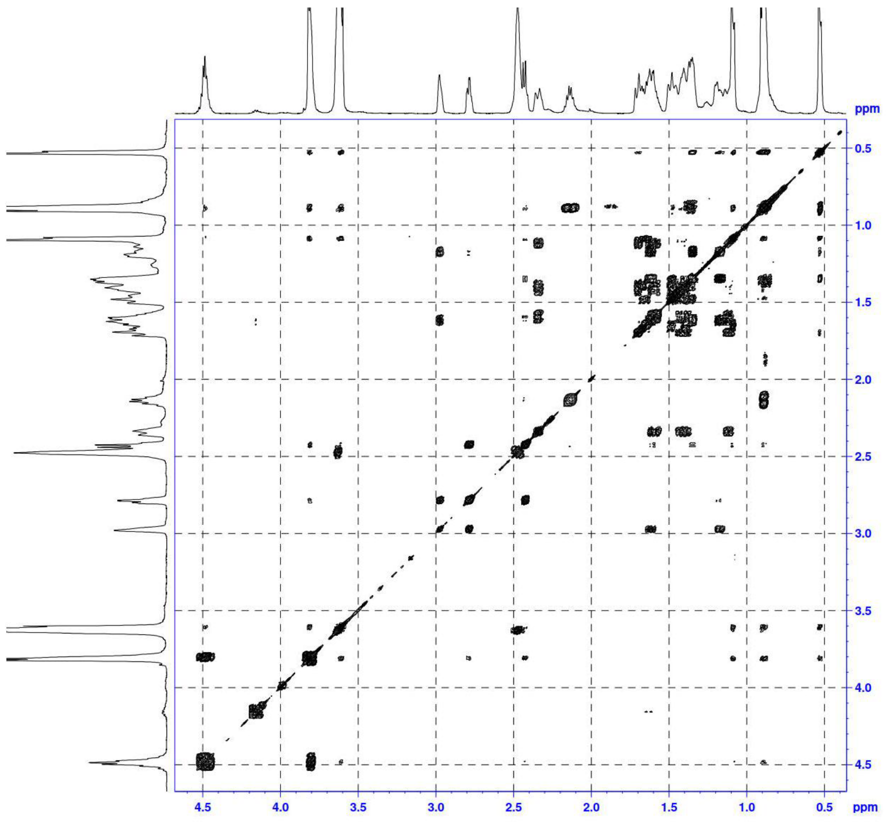

Figure S82. ^1H-^{13}C HSQC spectrum of compound **16** in $CDCl_3$

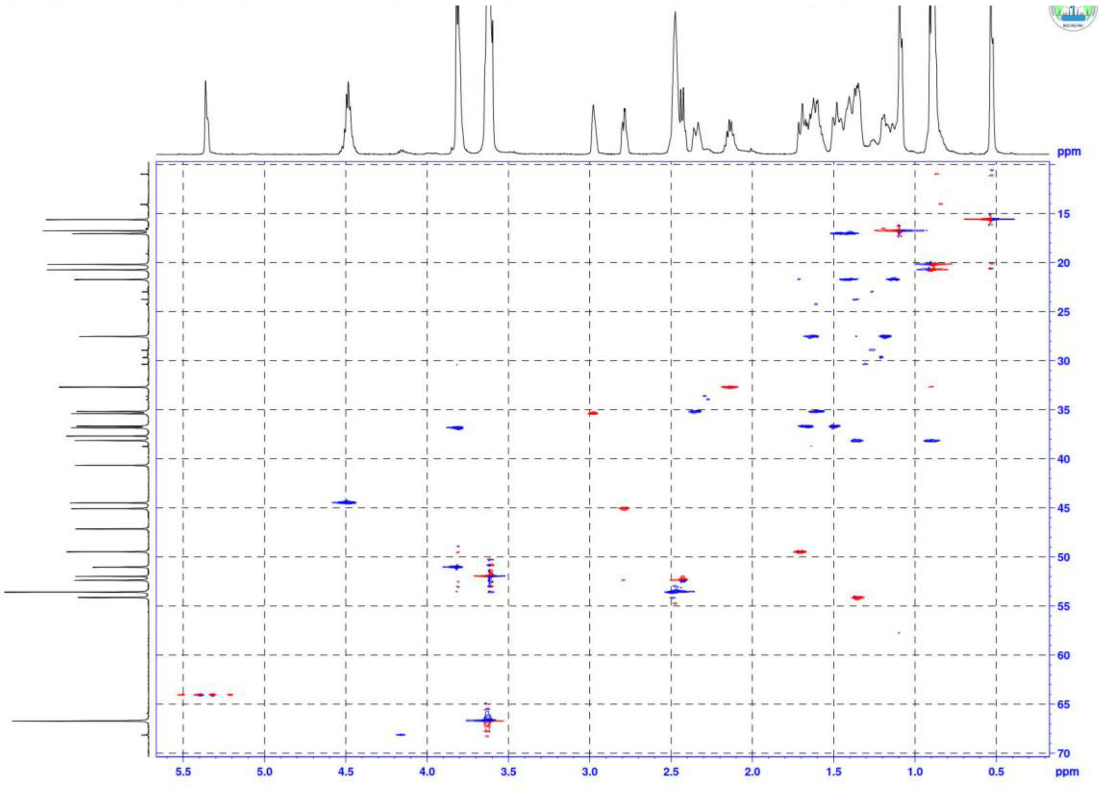

Figure S83. ^{1}H-^{13}C HMBC spectrum of compound **16** in $CDCl_3$

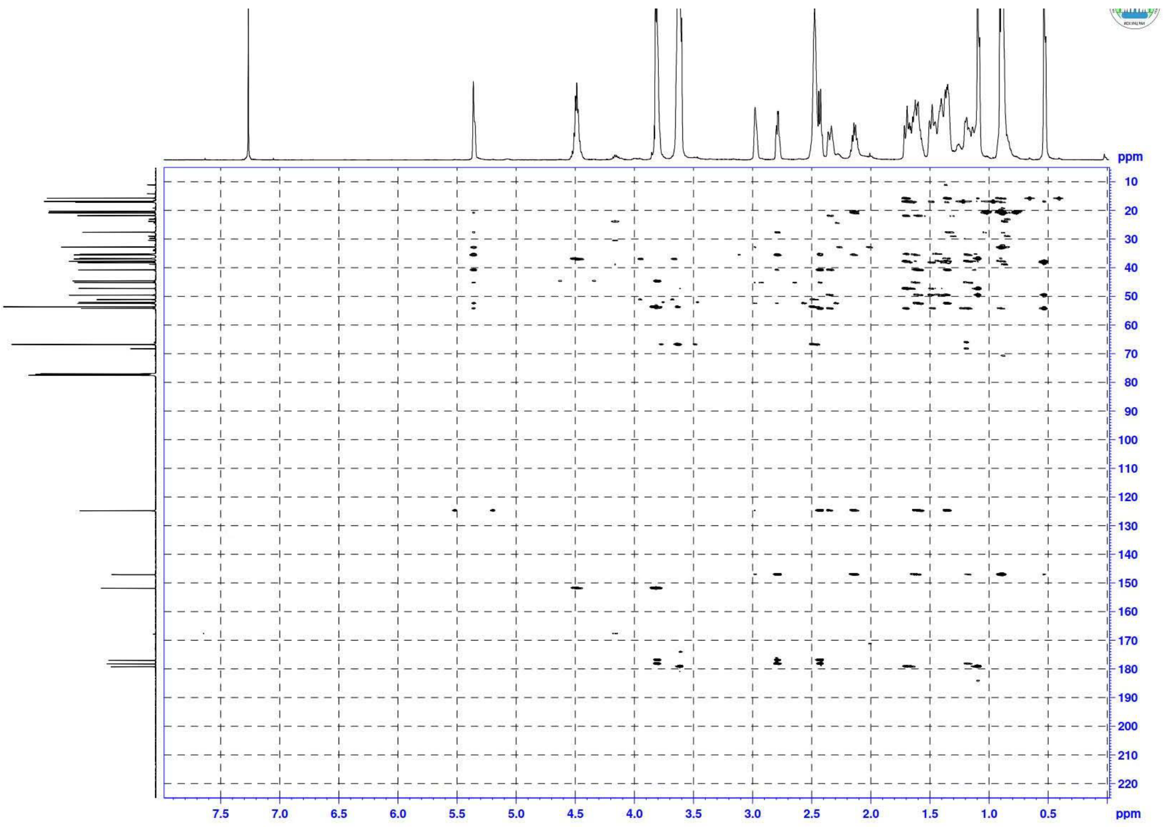

Figure S84. ^{1}H-^{1}H NOESY spectrum of compound **16** in $CDCl_3$

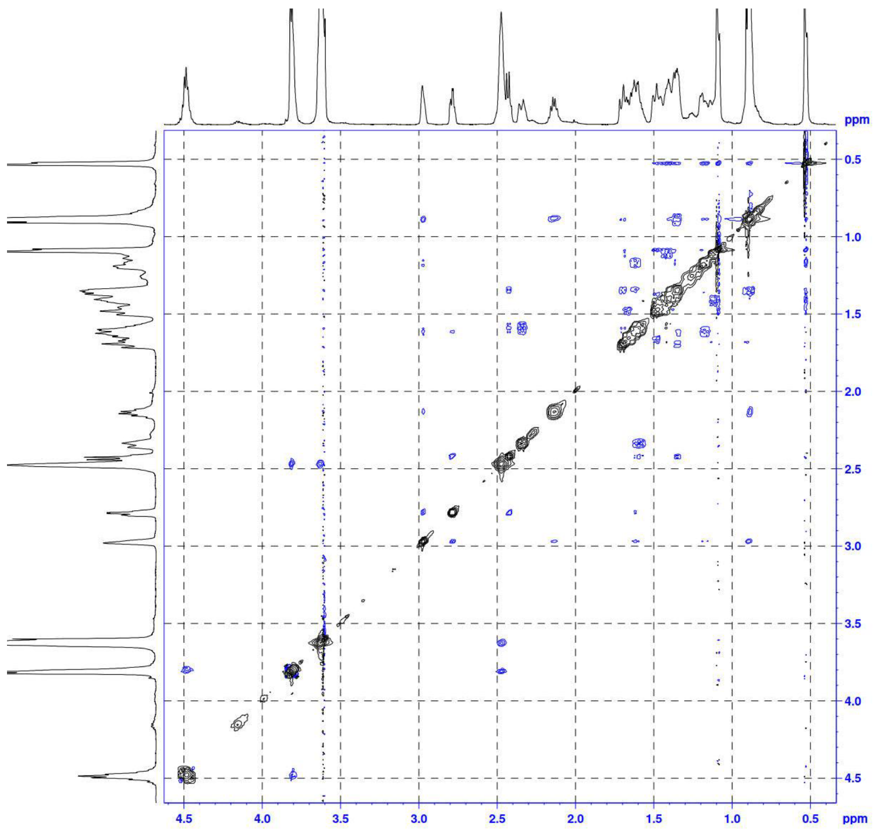

Figure S85. ^{1}H-^{15}N HMBC spectrum of compound **16** in $CDCl_3$

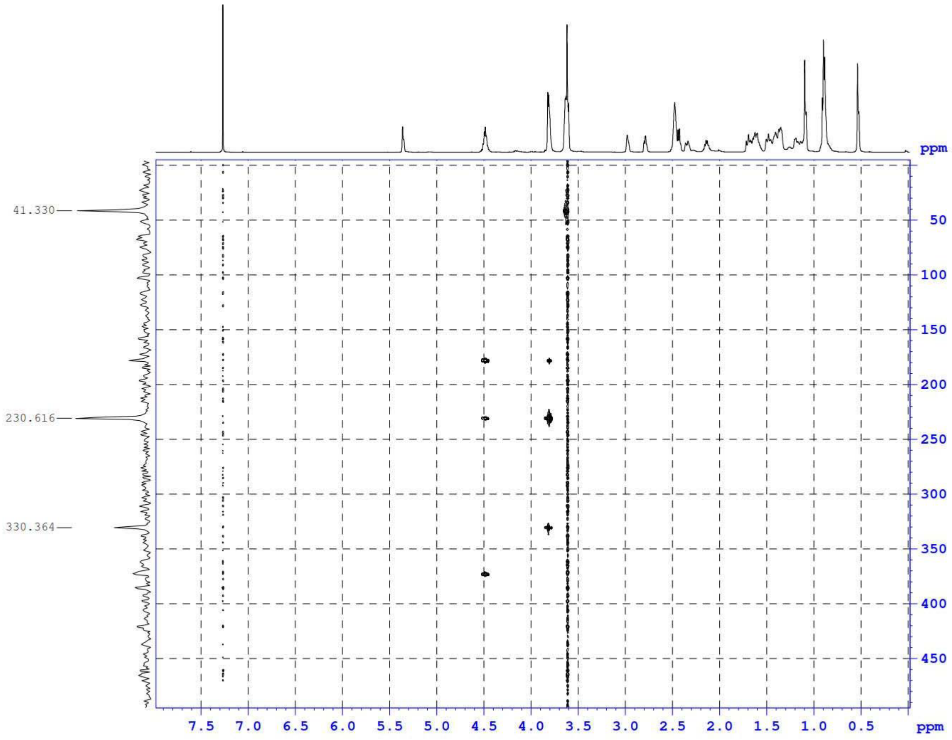

Figure S86. ^{1}H NMR spectrum of compound **17** in $CDCl_3$ (500 MHz)

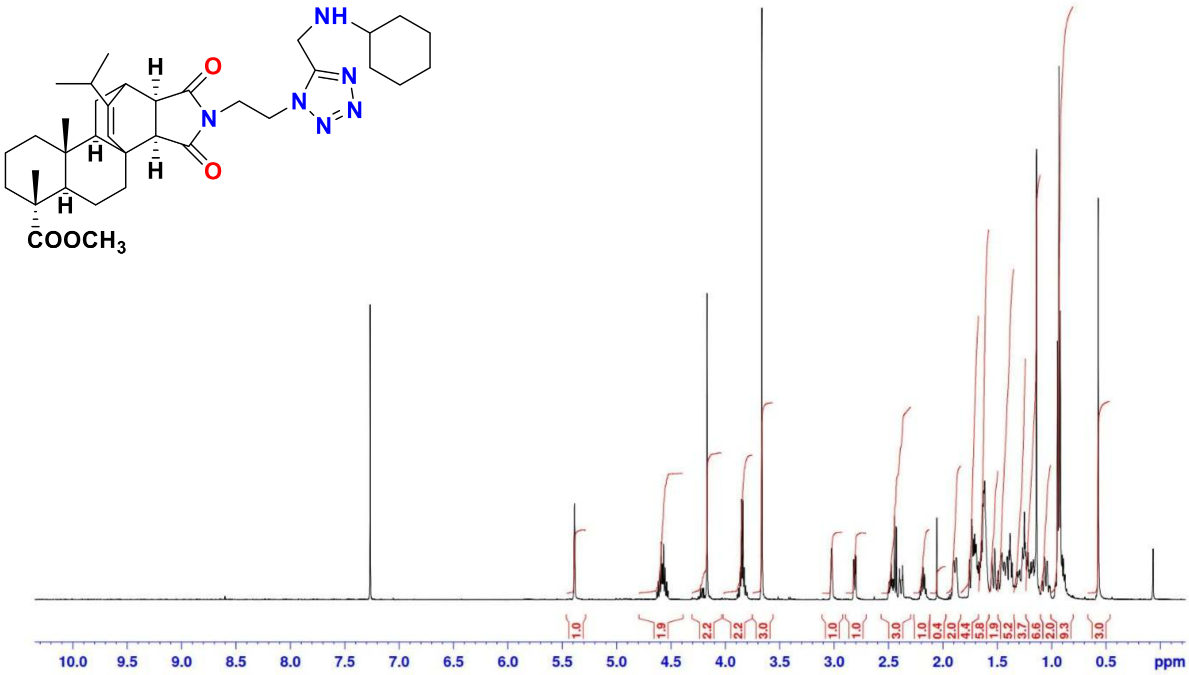

Figure S87. ^{13}C NMR spectrum of compound **17** in $CDCl_3$ (125 MHz)

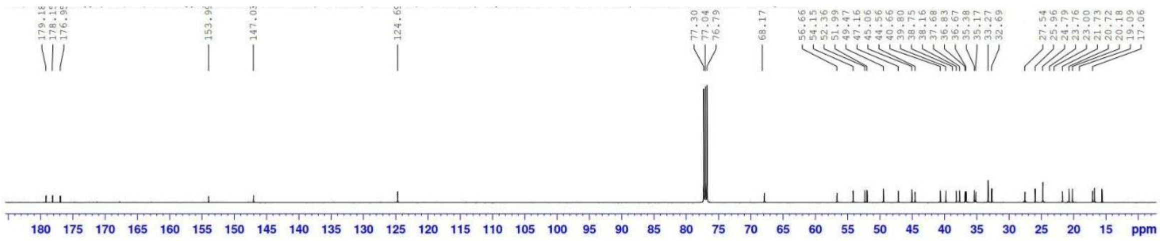

Figure S88. ^{1}H-^{1}H COSY spectrum of compound **17** in $CDCl_3$

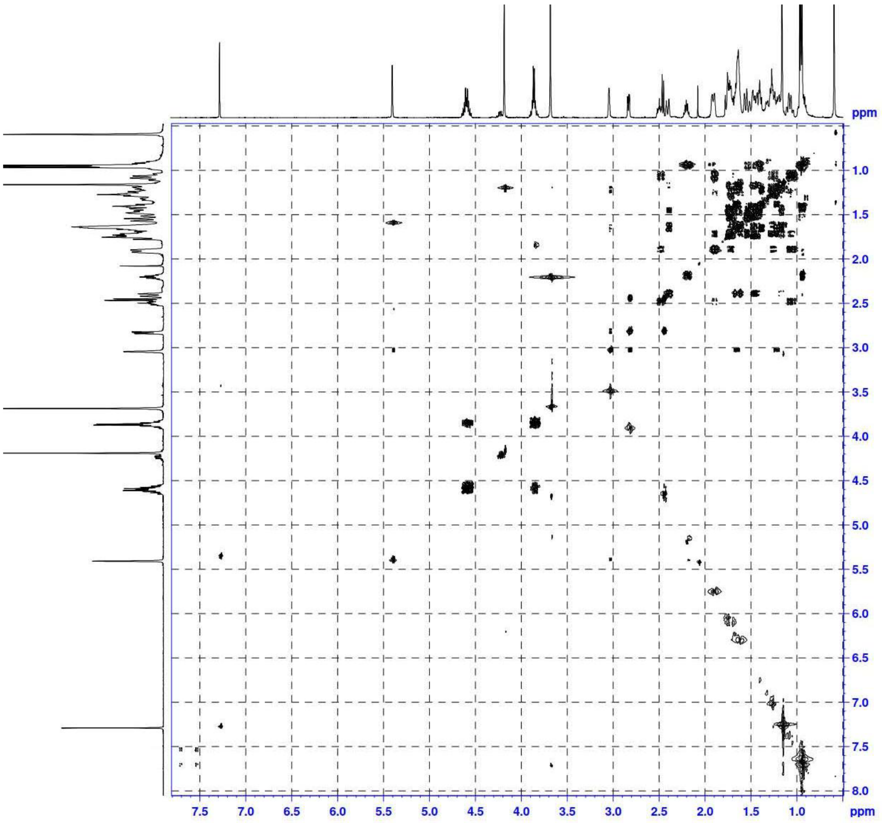

Figure S89. ^{1}H-^{13}C HSQC spectrum of compound **17** in $CDCl_3$

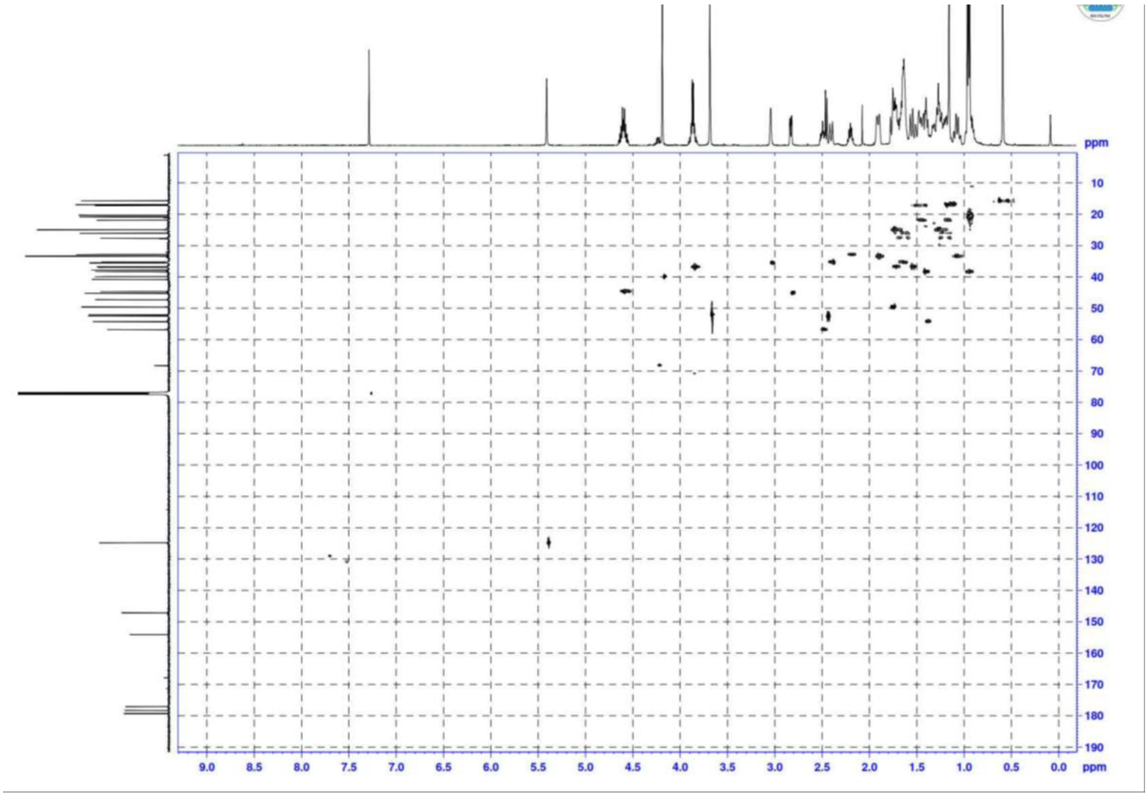

Figure S90. ^{1}H-^{13}C HMBC spectrum of compound **17** in $CDCl_3$

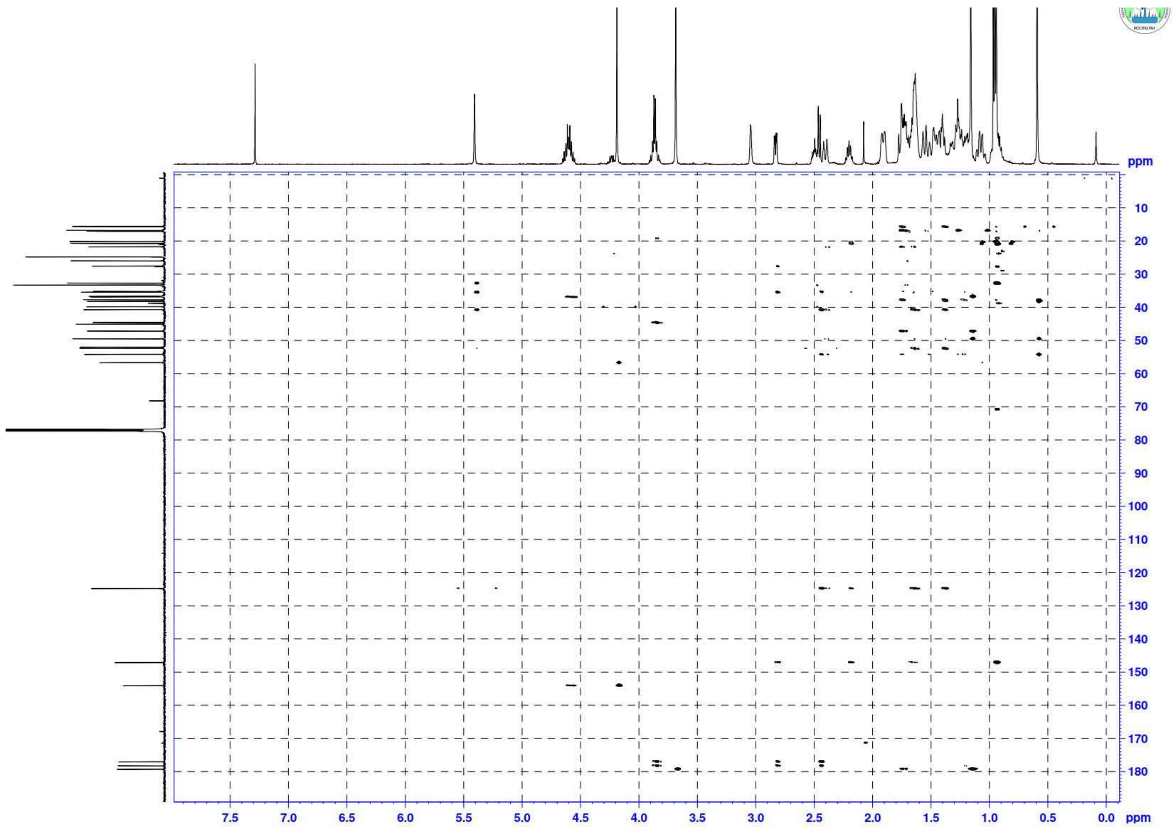

Figure S91. ^{1}H-^{1}H NOESY spectrum of compound **17** in $CDCl_3$

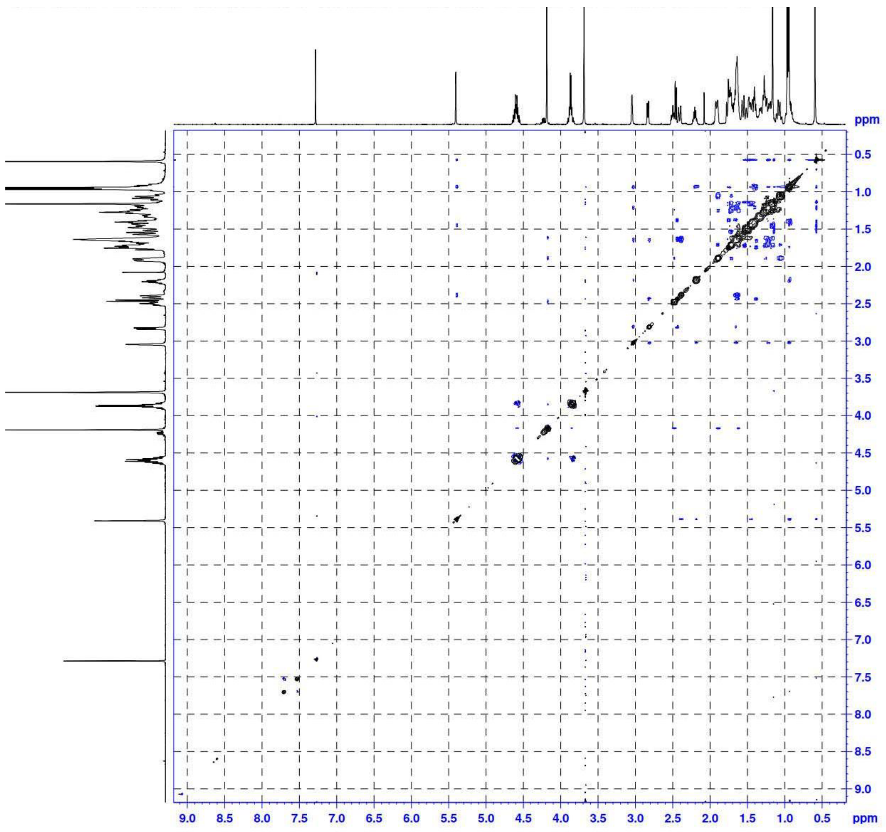

Figure S92. ^{1}H-^{15}N HMBC spectrum of compound **17** in $CDCl_3$

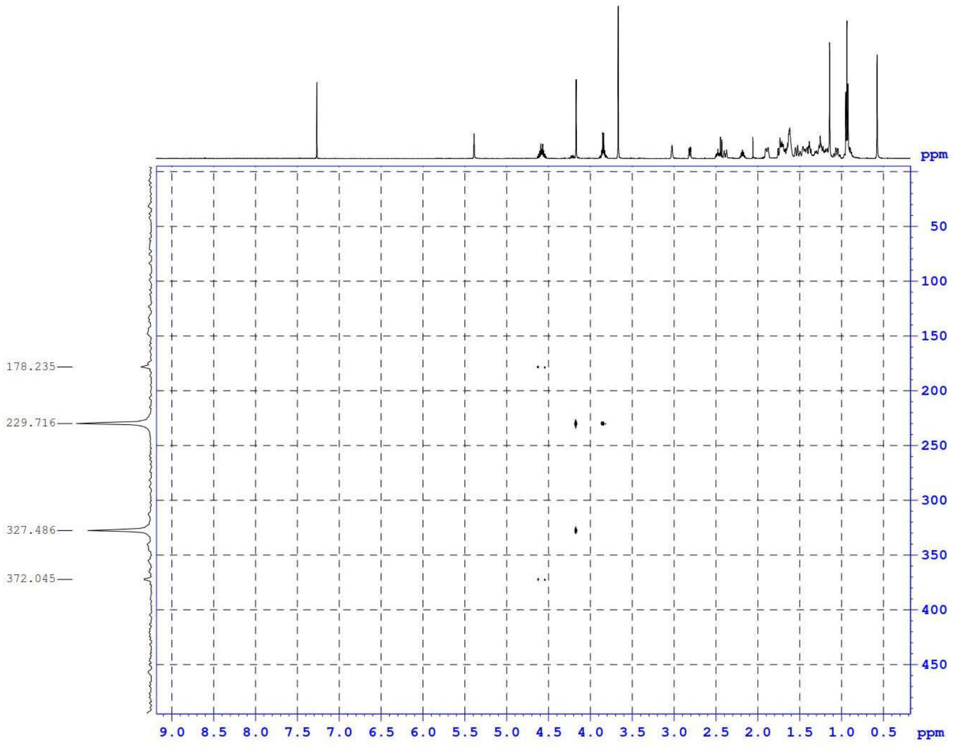

Figure S93. ^{1}H NMR spectrum of compound **18** in $CDCl_3$ (500 MHz)

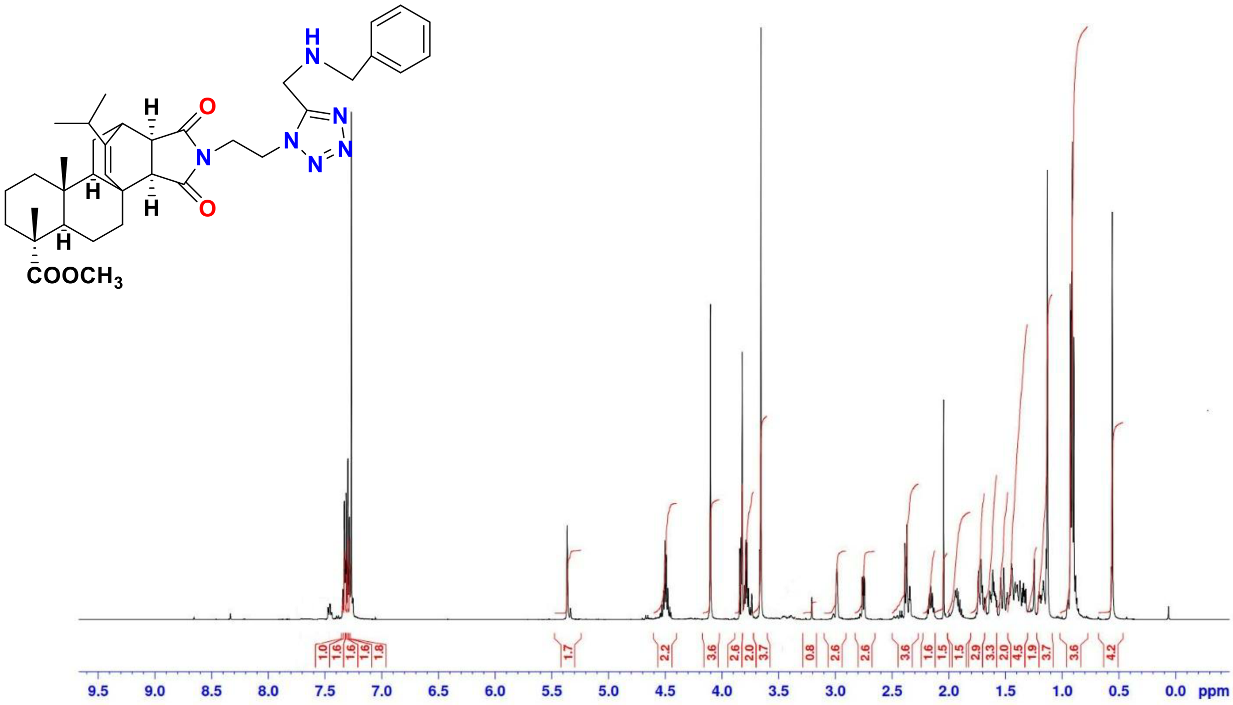

Figure S94. ^{13}C NMR spectrum of compound **18** in $CDCl_3$ (125MHz)

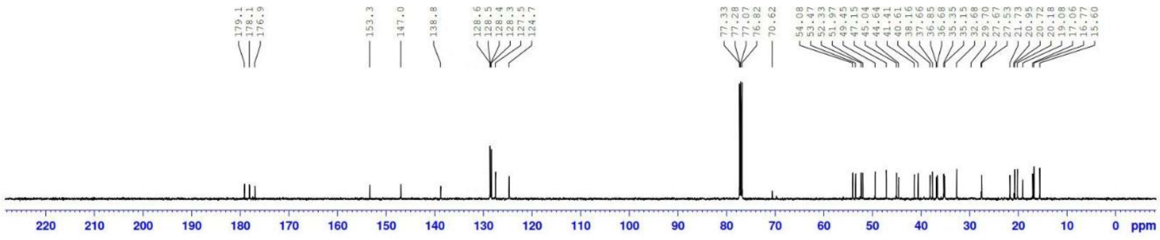

Figure S95. ^{1}H-^{1}H COSY spectrum of compound **18** in $CDCl_3$

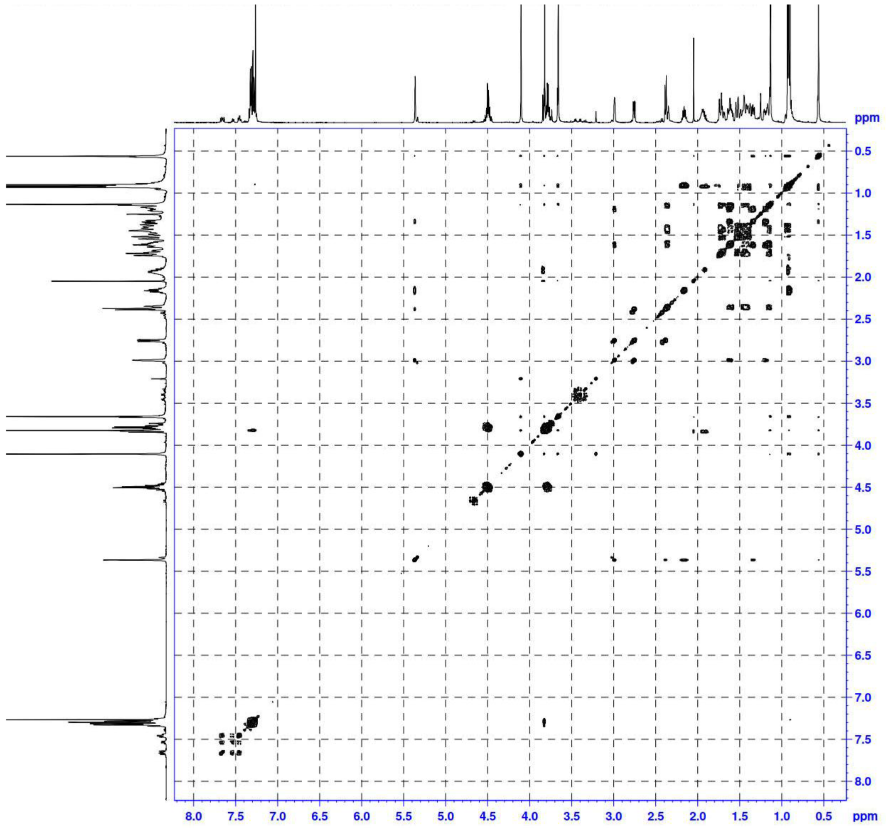

Figure S96. NOESY spectrum of compound **18** in $CDCl_3$

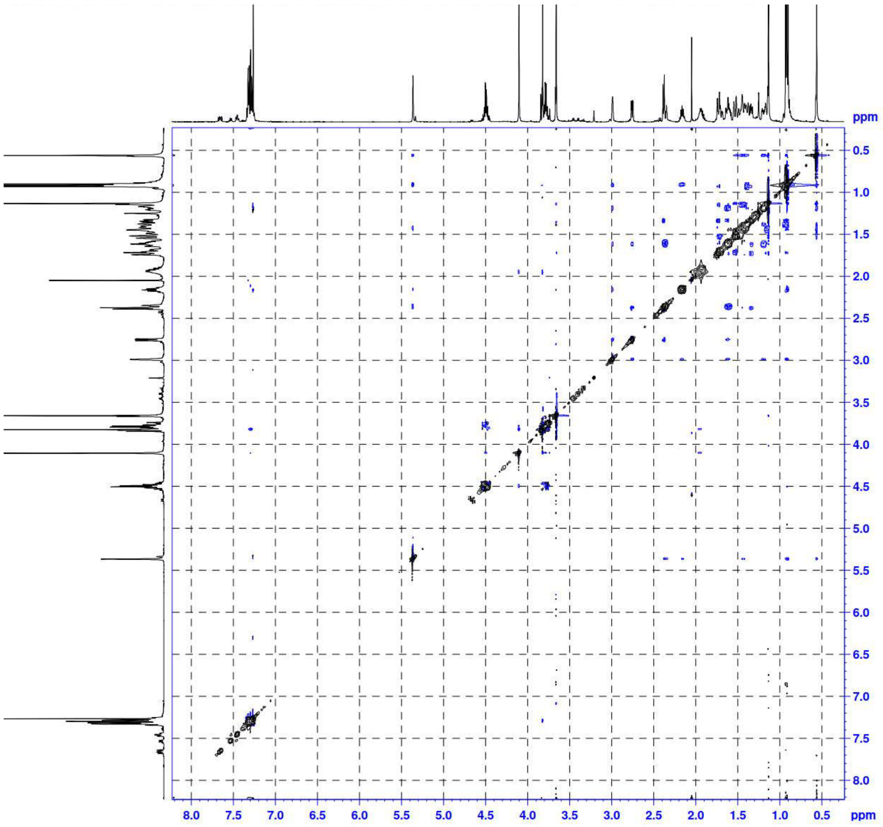

Figure S97. ^{1}H-^{13}C HSQC spectrum of compound **18** in $CDCl_3$

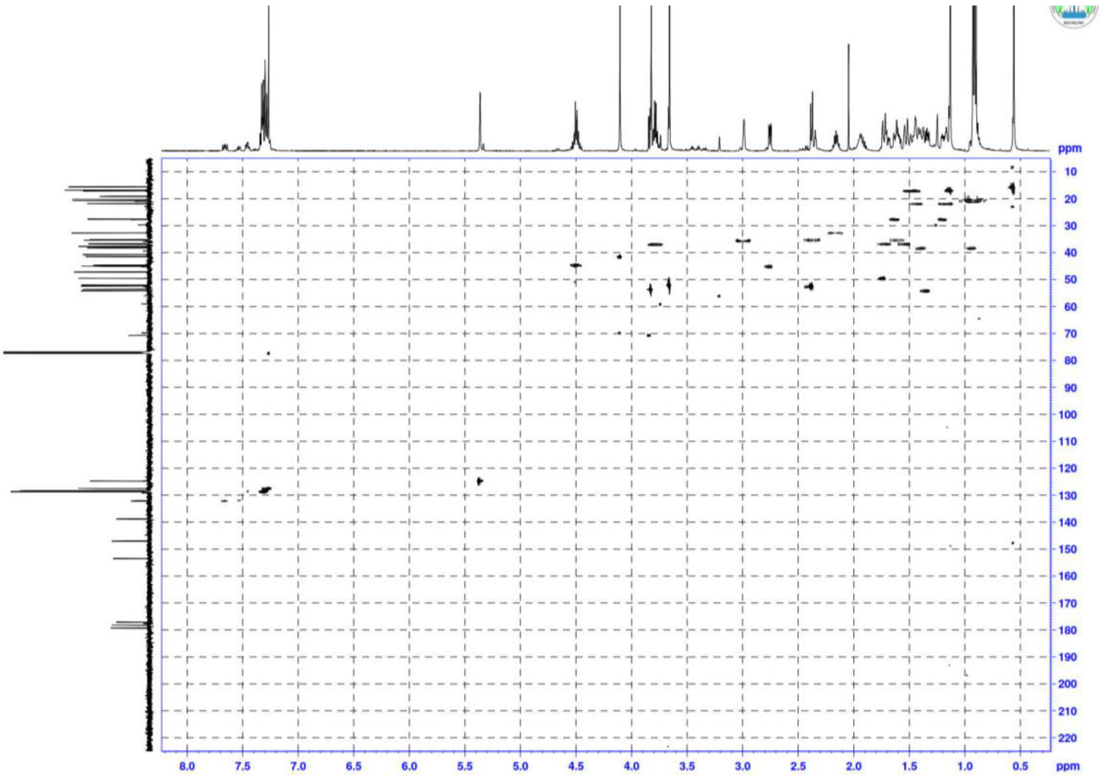

Figure S98. ^{1}H-^{13}C HMBC spectrum of compound **18** in $CDCl_3$

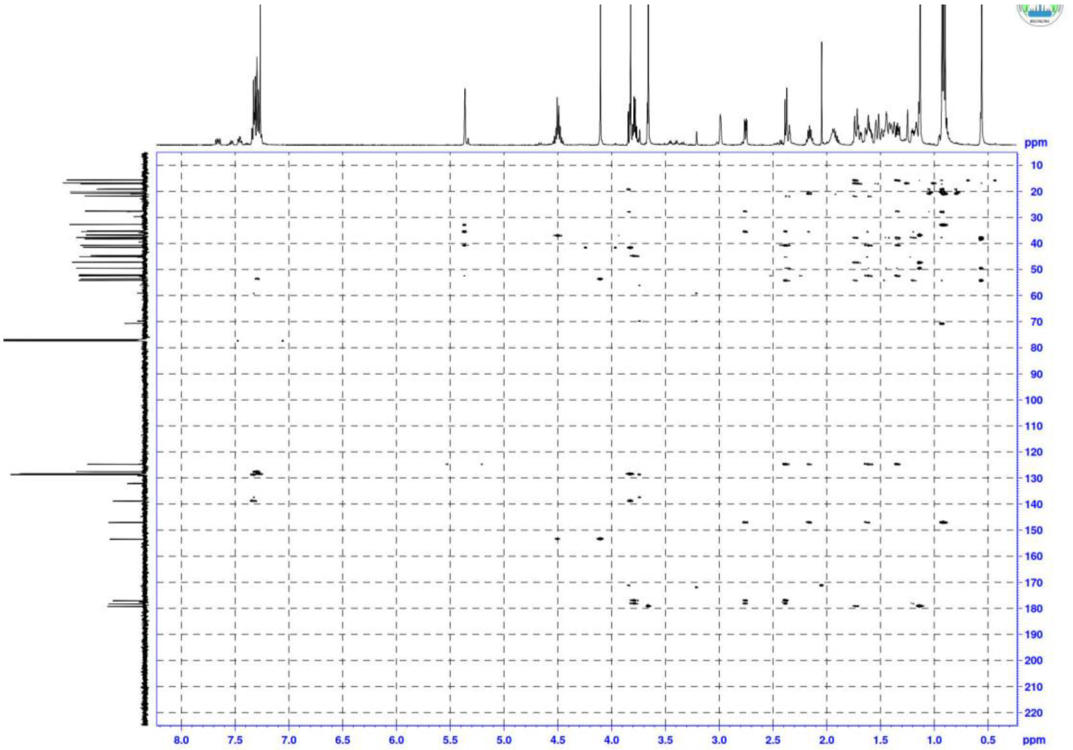

Figure S99. ^1H-^{15}N HMBC spectrum of compound **18** in $CDCl_3$

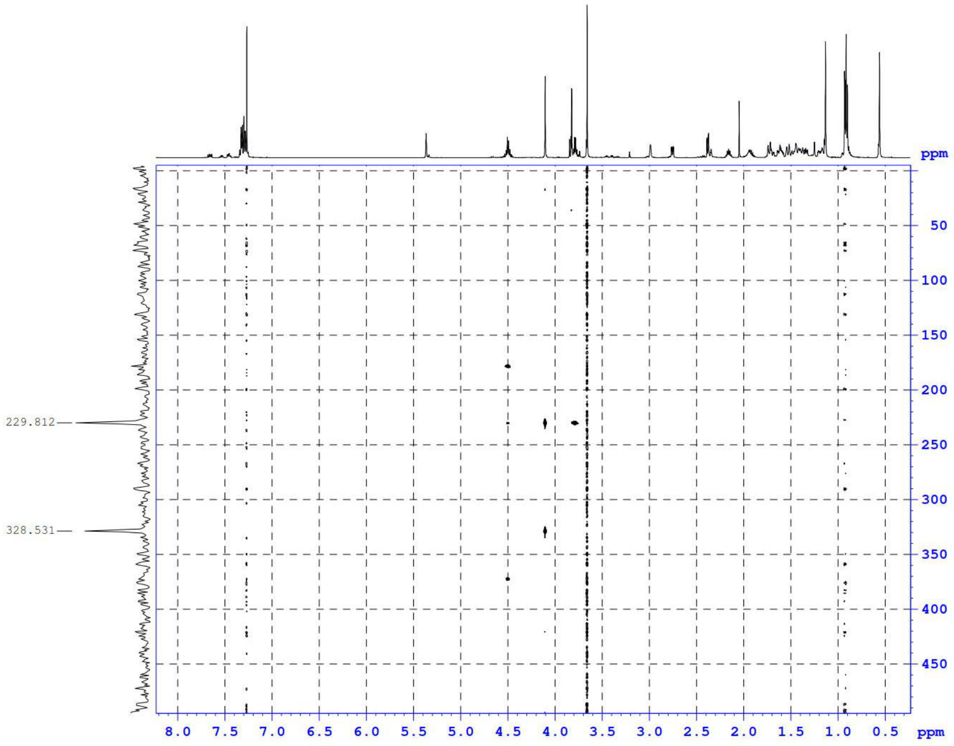

Figure S100. HPLC spectrum of compound **2**

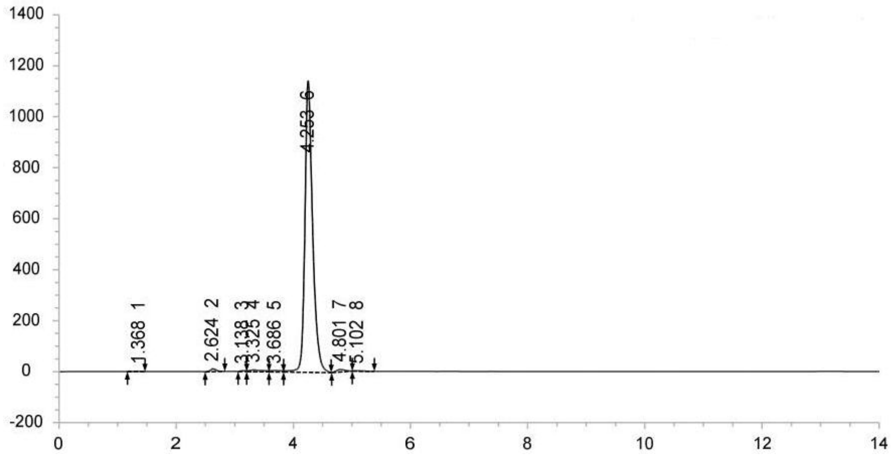

Figure S101. HPLC spectrum of compound **3**

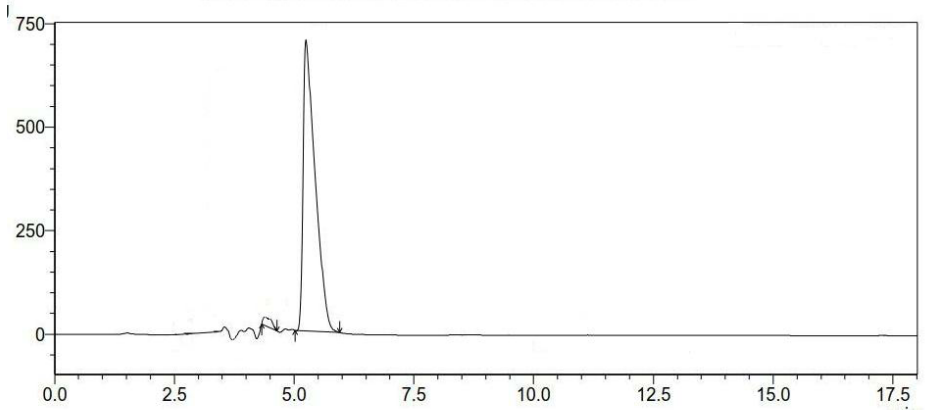

Figure S102. HPLC spectrum of compound **4**

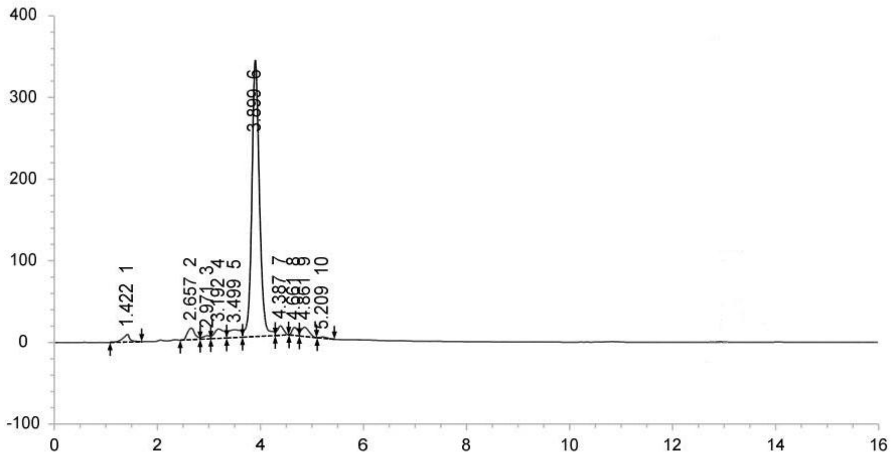

Figure S103. HPLC spectrum of compound **5**

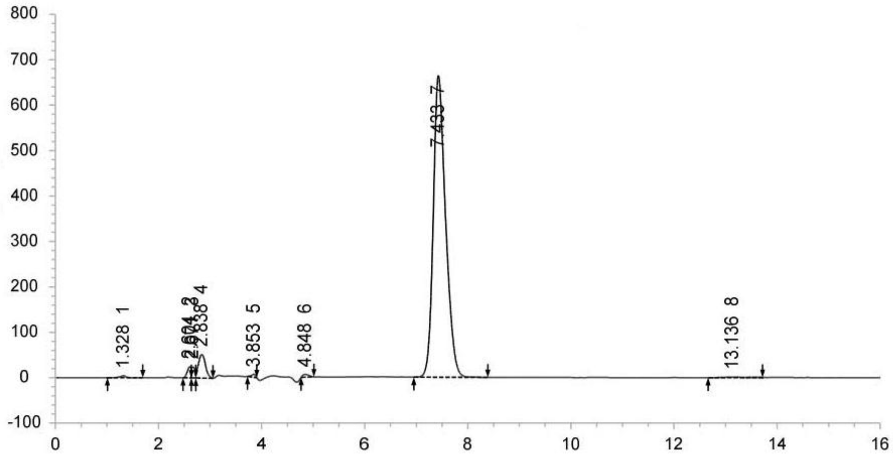

Figure S104. HPLC spectrum of compound **6**

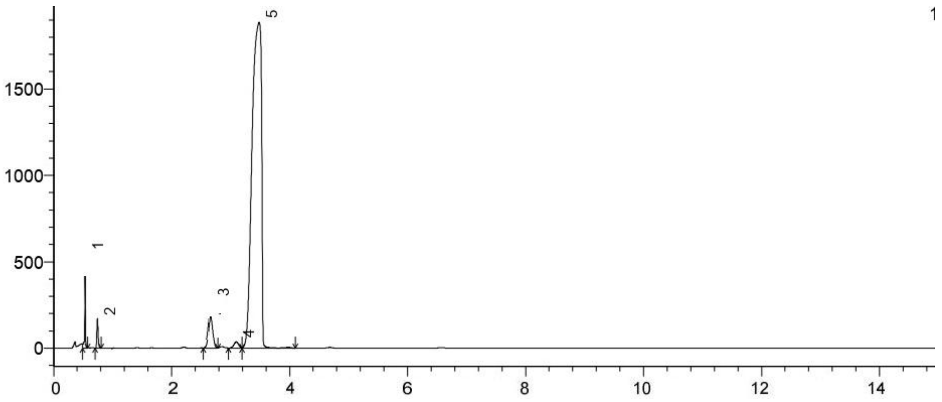

Figure S105. HPLC spectrum of compound **7**

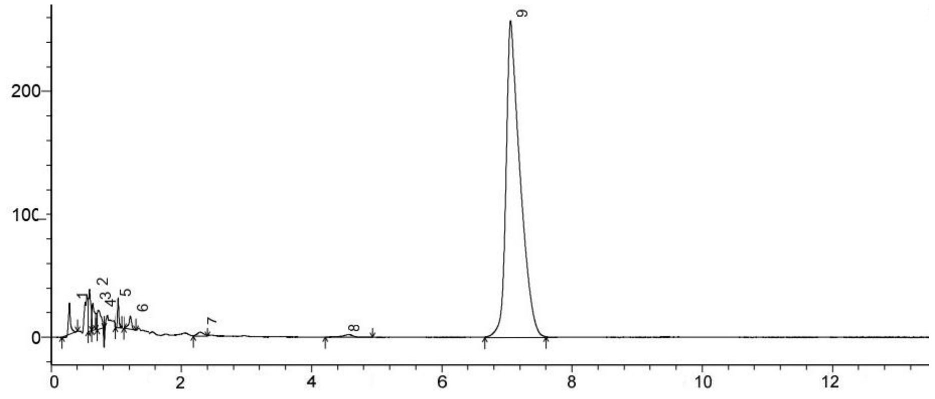

Figure S106. HPLC spectrum of compound **8**

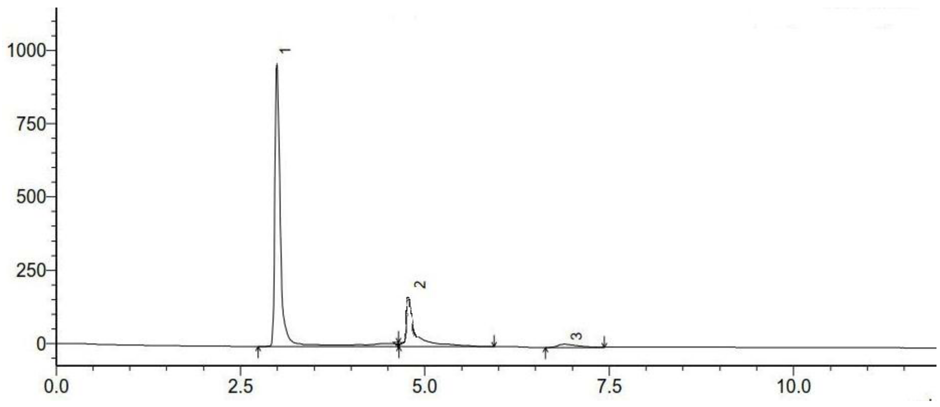

Figure S107. HPLC spectrum of compound **9**

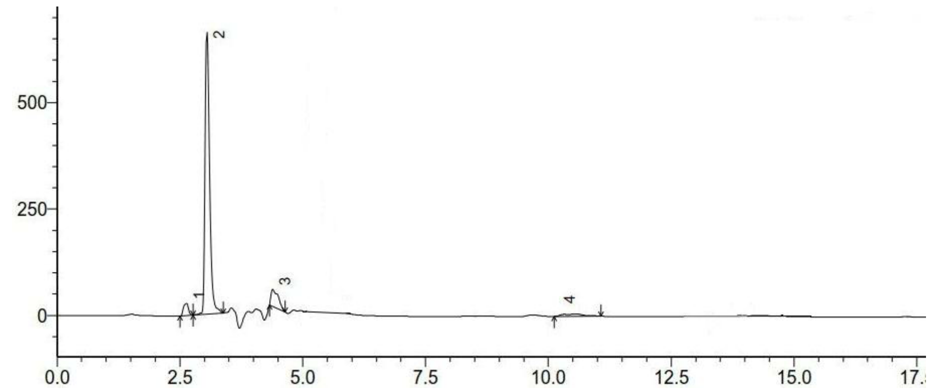

Figure S108. HPLC spectrum of compound **10**

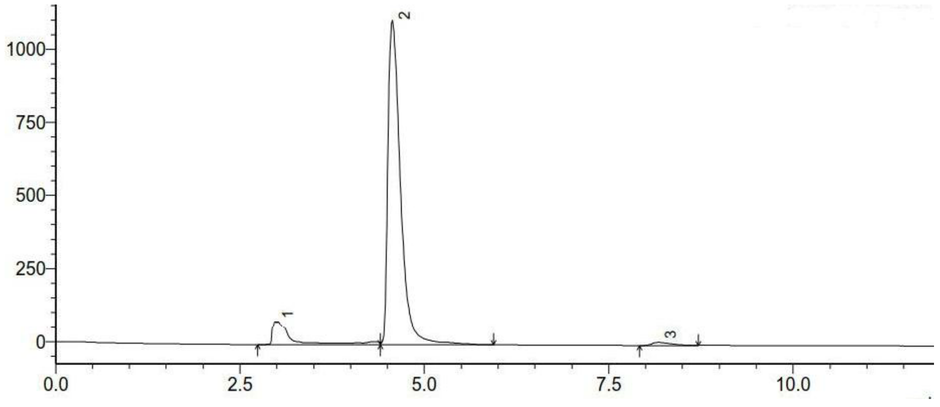

Figure S109. HPLC spectrum of compound **11**

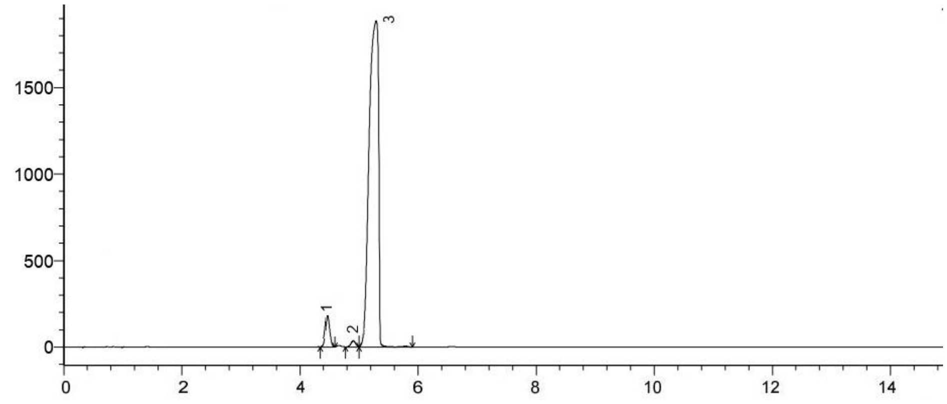

Figure S110. HPLC spectrum of compound **12**

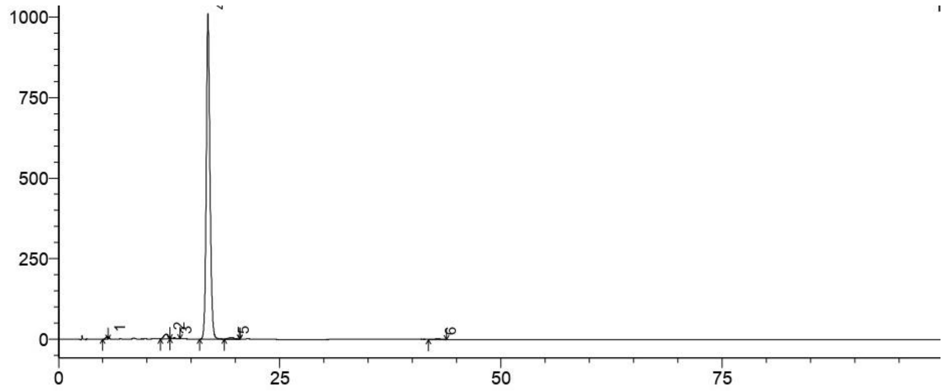

Figure S111. HPLC spectrum of compound **13**

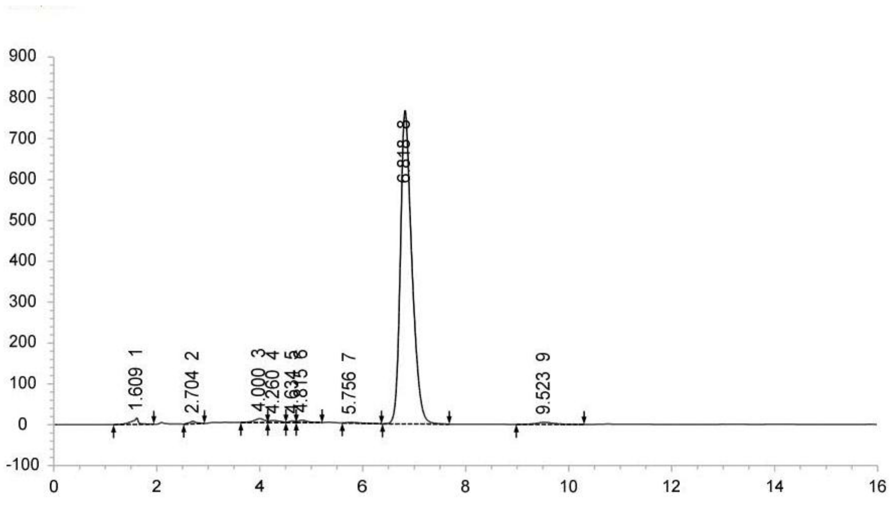

Figure S112. HPLC spectrum of compound **14**

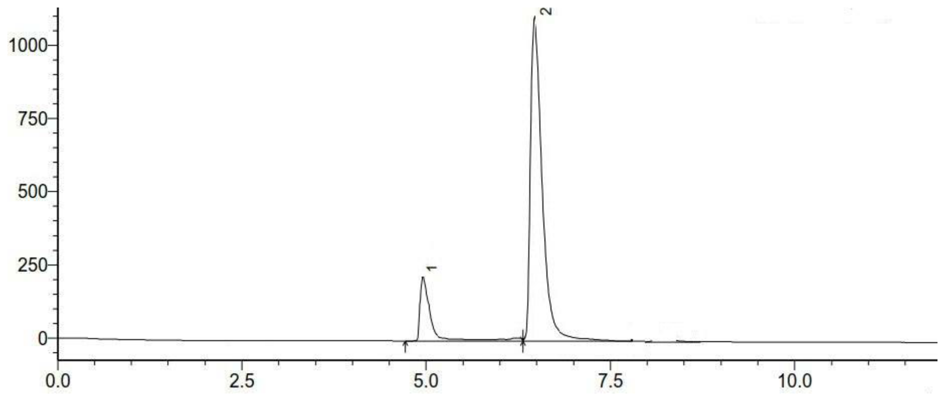

Figure S113. HPLC spectrum of compound **15**

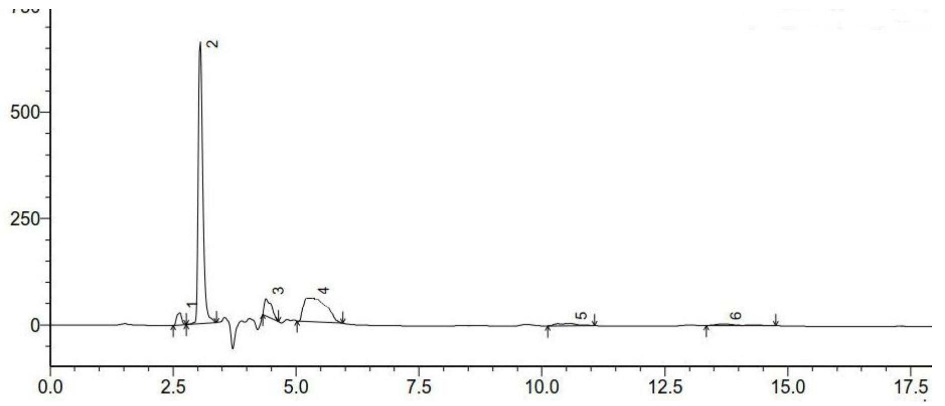

Figure S114. HPLC spectrum of compound **17**

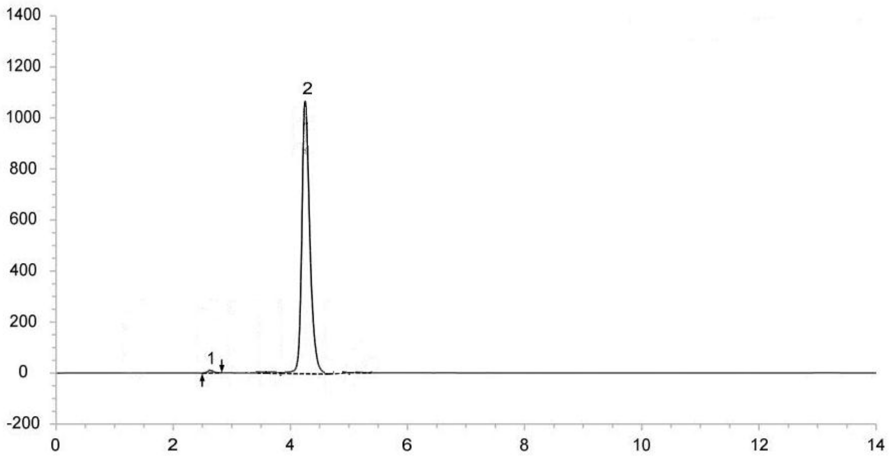

Figure S115. HPLC spectrum of compound **18**

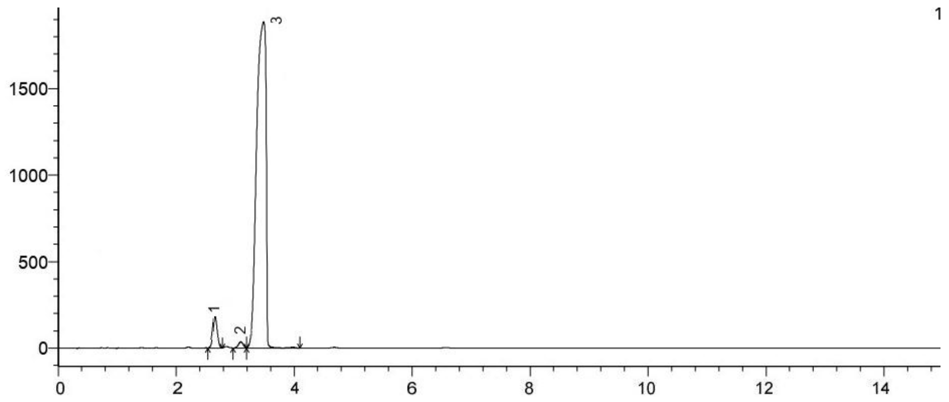

Supplement: Supplementary file 1 [file ijms-27-03494-s001.zip › ijms-4152244-supplementary.pdf]
